# Supplementary material for: Egg and cholesterol consumption and mortality from cardiovascular and different causes in the United States: A population-based cohort study
Source: PLoS Med. 2021 Feb 9;18(2):e1003508. doi: 10.1371/journal.pmed.1003508 (PMC7872242; doi:10.1371/journal.pmed.1003508)
Supplement: S1 Data — (DOCX) [file pmed.1003508.s002.docx]

**Supplementary Data**

**Table of Contents**

[Fig A. Flow chart of participants in current prospective cohort 2](#_Toc54710767)

[Fig B. Associations of whole egg and cholesterol intakes with all-cause mortality 3](#_Toc54710768)

[Table A. Categories for causes of death 4](#_Toc54710769)

[Table B. Baseline characteristics of participants according to cholesterol consumption 5](#_Toc54710770)

[Table C. Baseline characteristics of participants according to egg whites/substitutes consumption 7](#_Toc54710771)

[Table D. Spearman correlations between egg or cholesterol and other dietary factors 9](#_Toc54710772)

[Table E. Associations of egg whites/substitutes consumption with all-cause, CVD, and cancer mortality 10](#_Toc54710773)

[Table F. Data source of Figure 1. Multivariable-adjusted HRs (95% CIs) of all-cause and cause-specific mortality according to egg and cholesterol consumption 12](#_Toc54710774)

[Table G. Multivariable-adjusted HRs (95% CIs) of heart disease and stroke mortality 13](#_Toc54710775)

[Table H. Results of mediation analysis between whole egg and cholesterol for all-cause, CVD, and cancer mortality 14](#_Toc54710776)

[Table I. Multivariable-adjusted HRs (95% CIs) of mortality from non-CVD and non-cancer causes according to whole egg consumption 15](#_Toc54710777)

[Table J. Multivariable-adjusted HRs (95% CIs) of mortality from non-CVD and non-cancer causes according to cholesterol consumption 16](#_Toc54710778)

[Table K. Multivariable-adjusted HRs (95% CIs) of mortality from non-CVD and non-cancer causes according to egg whites/substitutes consumption 17](#_Toc54710779)

[Table L. Multivariable-adjusted HRs (95% CIs) of all-cause and cause-specific mortality according to fried eggs, non-fried eggs, egg whites, and egg substitutes consumption 18](#_Toc54710780)

[Table M. Multivariable-adjusted HRs (95% CIs) of mortality according to whole egg consumption based on convenient cutoffs 19](#_Toc54710781)

[Table N. Multivariable-adjusted HRs (95% CIs) of mortality according to cholesterol intake based on convenient cutoffs 21](#_Toc54710782)

[Table O. Sensitivity analyses for the associations of egg and cholesterol consumption with mortality by excluding participants with extreme BMIs 23](#_Toc54710783)

[Table P. Sensitivity analyses for the associations of egg and cholesterol consumption with mortality by further adjusting for perceived health condition 25](#_Toc54710784)

[Table Q. Sensitivity analyses for the associations of egg and cholesterol consumption with mortality by further adjusting for a propensity score 27](#_Toc54710785)

[Table R. Sensitivity analyses for the associations of egg and cholesterol consumption with mortality by further adjusting for the use of vitamin supplements and aspirin 29](#_Toc54710786)

[Table S. Sensitivity analyses for the associations of egg and cholesterol consumption with mortality by carefully adjusting for processed meat 31](#_Toc54710787)

[Table T. Sensitivity analyses for the associations of egg and cholesterol consumption with mortality by further adjusting for cholesterol-lowering medications (n=293,918) 33](#_Toc54710788)

[Table U. Sensitivity analyses for the associations of egg and cholesterol consumption with mortality by excluding the initial 5 years of follow-up 35](#_Toc54710789)

[Table V. Sensitivity analyses for the associations of egg and cholesterol consumption with mortality by excluding participants with CVD, cancer, or diabetes at baseline 37](#_Toc54710790)

[Table W. Sensitivity analyses for the associations of egg and cholesterol consumption with mortality by censoring participants at 8-y follow-up 39](#_Toc54710791)


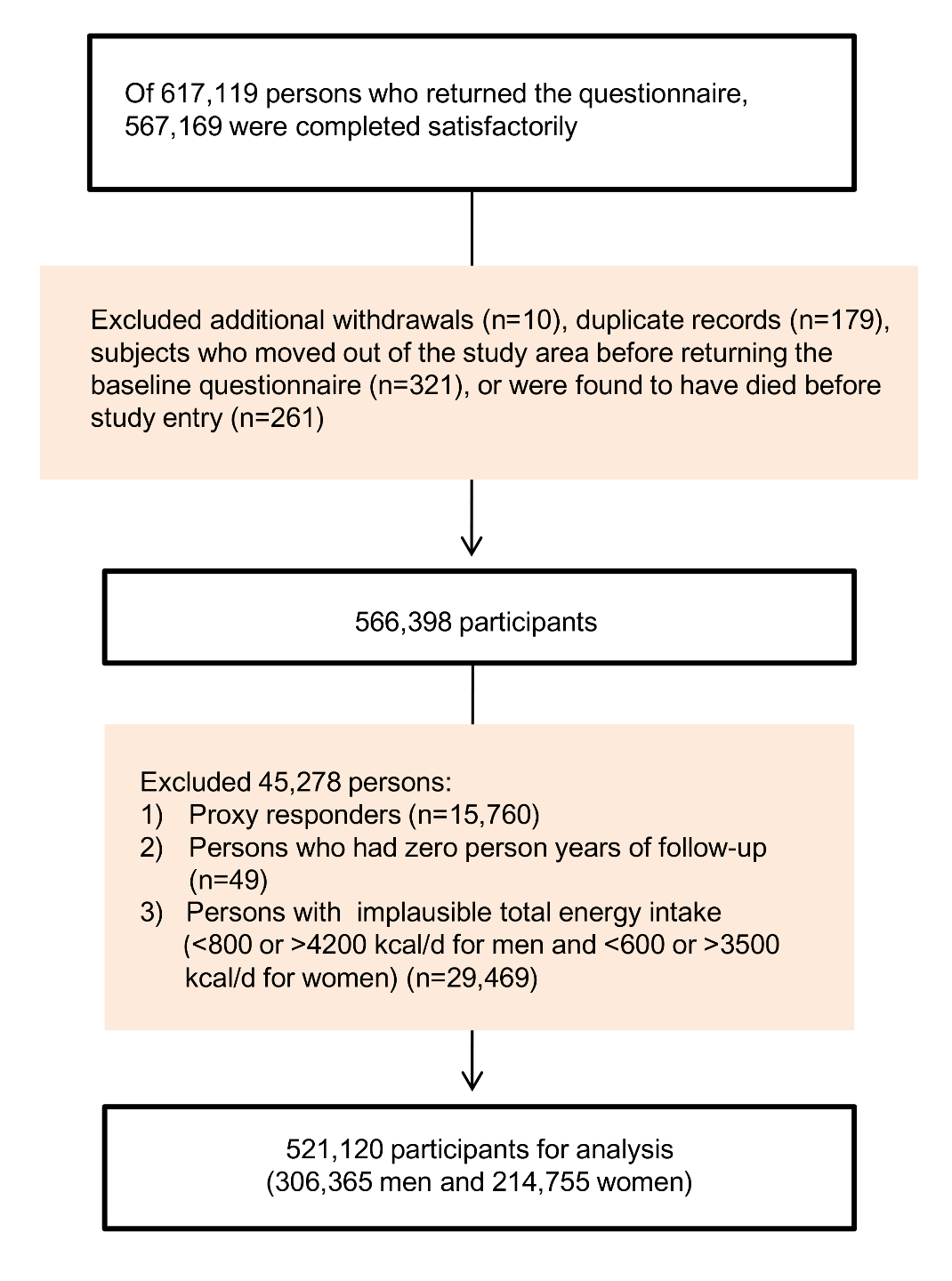


# Fig A. Flow chart of participants in current prospective cohort

# Fig B. Associations of whole egg and cholesterol intakes with all-cause mortality


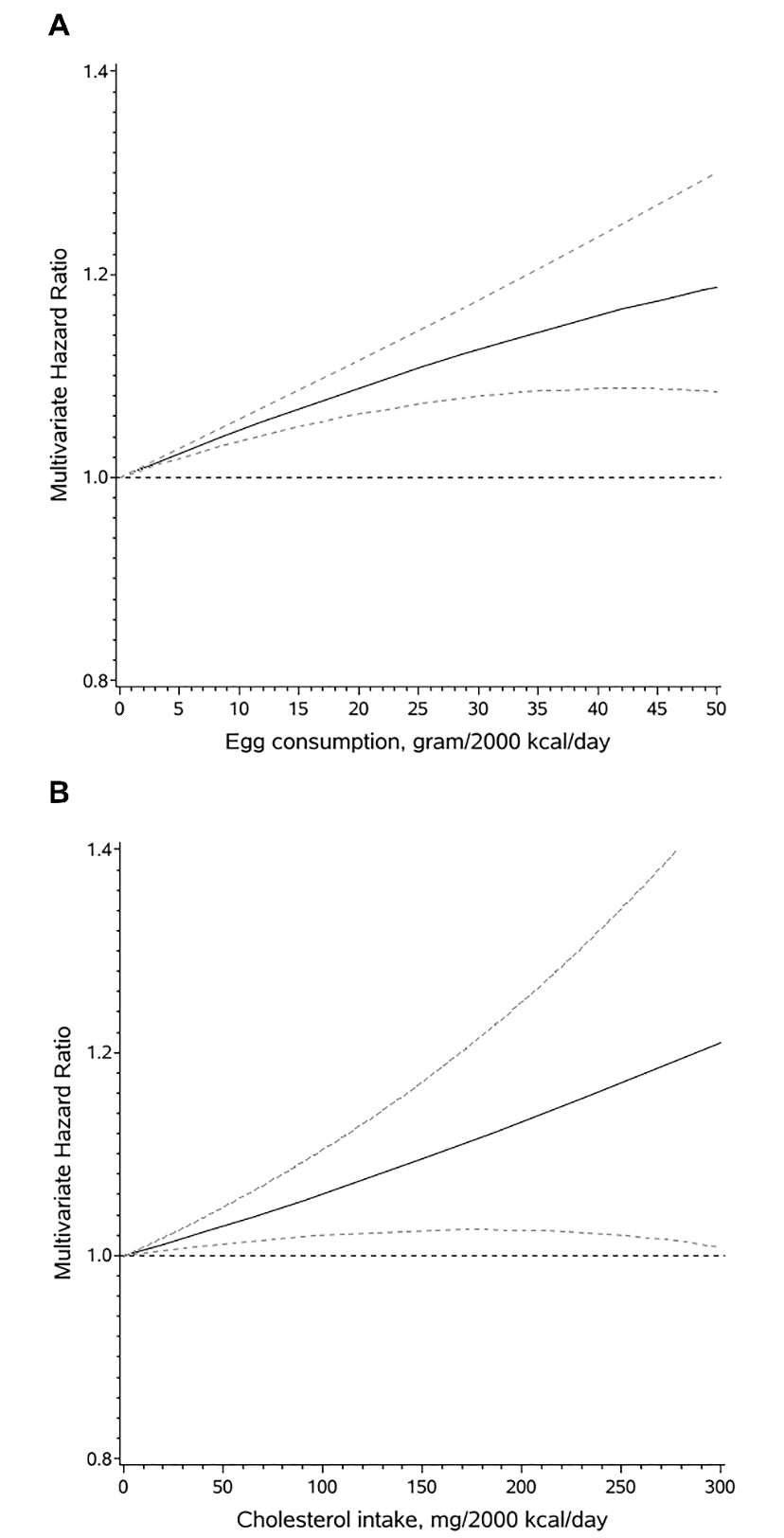


Nonparametric regression curves show the association of (**A**) whole egg and (**B**) cholesterol intakes with all-cause mortality. Multivariate hazard ratios are estimated by restricted-cubic-spline regression adjusted for age, sex, BMI, race, education, marital status, household income, smoking, alcohol, vigorous physical activity, usual activity at work, hypertension, hypercholesteremia, history of heart disease, stroke, diabetes, and cancer at baseline, total energy intake, and egg whites/substitutes and Healthy Eating Index-2015 (for whole egg), or intakes of saturated fat, polyunsaturated fat, monounsaturated fat, trans fat, animal protein, fiber, and sodium (for cholesterol). Grey dotted lines represent 95% confidence intervals (CIs).

# Table A. Categories for causes of death

| **Causes of Death** | **ICD-9 Code** | **ICD-10 Code** |
| --- | --- | --- |
| Cancer | 140-239 | C00-C97 and D00-D48 |
| Alzheimer's disease | 331 | G30 |
| Cardiovascular disease | 390-398, 401-404, 410-429 and 440-448 | I00-I13, I20-I51 and I70-I78 |
| Diabetes | 250 | E10-E14 |
| Respiratory disease | 480-487 and 490-496 | J10-J18 and J40-J47 |
| Infections | 001-139 | A00-B99 |
| Kidney disease | 580-589 | N00-N07, N17-N19 and N25-N27 |
| Chronic liver disease | 571 | K70, K73-K74 |
| Injuries and accidents | 800-978 | V01-X59, Y85-Y86, U03, X60-X84, Y87.0, U01-U02, X85-Y09, Y35, Y87.1, and Y89.0 |

Detailed corresponding ICD codes for these categories are available at https://seer.cancer.gov/codrecode/1969+_d09172004/index.html.

# Table B. Baseline characteristics of participants according to cholesterol consumption

|  | **Quintiles of cholesterol consumption** | | | | |
| --- | --- | --- | --- | --- | --- |
| **Characteristics** | **Q1** | **Q2** | **Q3** | **Q4** | **Q5** |
| Range (mg/2000 kcal/day) | ≤147 | 148-189 | 190-228 | 229-282 | ≥283 |
| N | 104,224 | 104,224 | 104,224 | 104,224 | 104,224 |
| Age (y) | 63.4^a^ | 63.1 | 62.8 | 62.5 | 62.4 |
| Male (%) | 54.5 | 56.7 | 58.3 | 60.6 | 63.9 |
| Race (%) |  |  |  |  |  |
| Non-Hispanic white | 89.9 | 92.1 | 92.5 | 92.7 | 91.6 |
| Non-Hispanic black | 4.2 | 3.5 | 3.3 | 3.3 | 3.9 |
| Other/missing | 5.9 | 4.4 | 4.2 | 4.1 | 4.5 |
| BMI (kg/m^2^) | 25.1 | 25.9 | 26.5 | 26.8 | 27.5 |
| Married (%) | 64.5 | 68.7 | 69.9 | 70.4 | 68.5 |
| College graduate or postgraduate (%) | 42.6 | 40.6 | 39.2 | 37.8 | 34.9 |
| Annual household income (USD)^b^ | 49389.0 | 49116.0 | 49018.0 | 48739.0 | 47368.0 |
| Current smoker (%) | 7.5 | 9.4 | 11.2 | 13.3 | 16.9 |
| Physical activity, ≥5 times/wk (%) | 26.0 | 20.7 | 17.9 | 16.4 | 14.6 |
| Hypertension (%) | 23.9 | 23.3 | 23.1 | 23.5 | 23.3 |
| High cholesterol level (%) | 22.7 | 25.1 | 26.6 | 27.8 | 28.7 |
| Heart disease (%) | 18.0 | 14.7 | 13.0 | 12.3 | 12.0 |
| Stroke (%) | 2.2 | 2.1 | 2.0 | 2.0 | 2.2 |
| Cancer (%) | 9.0 | 8.8 | 9.1 | 8.8 | 9.2 |
| Diabetes (%) | 6.3 | 7.4 | 8.3 | 9.8 | 13.7 |
| Fair or poor health (%) | 11.4 | 11.6 | 12.2 | 13.1 | 16.0 |
| Currently using vitamin supplements (%) | 59.4 | 57.1 | 55.7 | 54.0 | 52.2 |
| Daily use of aspirin (%) | 18.3 | 15.8 | 14.2 | 13.5 | 12.7 |
| Egg whites or substitutes consumer (%) | 35.7 | 18.9 | 9.8 | 4.6 | 1.5 |
| Daily dietary intake^c^ |  |  |  |  |  |
| Total energy (kcal) | 1606.7 | 1668.8 | 1710.3 | 1751.3 | 1708.8 |
| Alcohol from alcoholic drinks (g) | 1.6 | 1.9 | 2.0 | 2.0 | 1.7 |
| Whole egg (g) | 0.8 | 4.2 | 7.6 | 12.2 | 26.3 |
| Cholesterol (mg) | 118.3 | 168.7 | 207.9 | 252.0 | 330.0 |
| Total fat (% of energy) | 22.7 | 27.7 | 30.6 | 33.0 | 36.1 |
| Saturated fat (% of energy) | 6.4 | 8.3 | 9.5 | 10.5 | 11.6 |
| Monounsaturated fat (% of energy) | 8.4 | 10.4 | 11.5 | 12.4 | 13.6 |
| Polyunsaturated fat (% of energy) | 5.8 | 6.6 | 6.9 | 7.1 | 7.4 |
| Trans fat (% of energy) | 1.5 | 1.9 | 2.0 | 2.2 | 2.3 |
| Animal protein (g) | 32.2 | 41.4 | 46.3 | 51.4 | 57.5 |
| Fiber (g) | 25.8 | 22.4 | 20.6 | 19.2 | 17.4 |
| Sodium (mg) | 2864.2 | 2992.6 | 3054.7 | 3118.5 | 3195.4 |
| Red meat (g) | 30.0 | 53.1 | 67.3 | 81.3 | 95.7 |
| Poultry (g) | 23.5 | 33.1 | 36.0 | 38.3 | 38.6 |
| Fish (g) | 11.5 | 14.8 | 16.4 | 18.1 | 19.2 |
| Dairy products (g) | 297.6 | 310.9 | 307.4 | 294.6 | 263.3 |
| Nuts/legumes (g) | 10.8 | 10.2 | 10.1 | 10.0 | 9.3 |
| Fruit (g) | 516.1 | 406.2 | 351.7 | 304.8 | 255.3 |
| Vegetables (g) | 342.6 | 310.9 | 299.6 | 289.9 | 275.3 |
| Potatoes (g) | 40.9 | 45.9 | 48.6 | 50.1 | 50.0 |
| Whole grains (g) | 35.6 | 30.9 | 27.9 | 25.1 | 21.6 |
| Refined grains (g) | 132.5 | 132.7 | 131.0 | 128.0 | 121.6 |
| Coffee (g) | 690.5 | 786.5 | 802.6 | 824.0 | 878.6 |
| Sugar-sweetened beverages (g) | 542.5 | 512.2 | 486.0 | 457.6 | 429.9 |
| Healthy Eating Index score | 73.5 | 71.3 | 69.0 | 66.6 | 63.2 |

BMI, body mass index; Q, quintile.

^a^ Values are medians or percentages unless stated otherwise.

^b^ Household income in 1999.

^c^ Dietary intakes were energy-adjusted using the nutrient density method of 2000 kcal per day unless stated otherwise.

# Table C. Baseline characteristics of participants according to egg whites/substitutes consumption

|  | **Egg whites or substitutes** | |
| --- | --- | --- |
| **Characteristics** | **Non-consumers** | **Consumers** |
| Range (g/2000 kcal/day) | 0 | >0 |
| n | 447,570 | 73,550 |
| Age (y) | 62.7^a^ | 63.7 |
| Male (%) | 58.4 | 60.9 |
| Race (%) |  |  |
| Non-Hispanic white | 91.7 | 92.4 |
| Non-Hispanic black | 3.7 | 3.0 |
| Other/missing | 4.6 | 4.6 |
| BMI (kg/m^2^) | 26.5 | 25.8 |
| Married (%) | 67.8 | 71.8 |
| College graduate or postgraduate (%) | 38.3 | 43.3 |
| Annual household income (USD)^b^ | 48529.0 | 50129.0 |
| Current smoker (%) | 12.7 | 5.6 |
| Physical activity, ≥5 times/wk (%) | 18.1 | 25.4 |
| Hypertension (%) | 22.7 | 27.4 |
| High cholesterol level (%) | 28.0 | 15.0 |
| Heart disease (%) | 11.8 | 27.2 |
| Stroke (%) | 2.0 | 2.8 |
| Cancer (%) | 9.0 | 9.2 |
| Diabetes (%) | 8.7 | 11.2 |
| Fair or poor health (%) | 12.6 | 14.7 |
| Currently using vitamin supplements (%) | 54.8 | 61.2 |
| Daily use of aspirin (%) | 13.6 | 23.1 |
| Daily dietary intake^c^ |  |  |
| Total energy (kcal) | 1705.2 | 1594.7 |
| Alcohol from alcoholic drinks (g) | 1.8 | 1.7 |
| Whole egg (g) | 9.4 | 0.0 |
| Cholesterol (mg) | 218.9 | 145.9 |
| Egg white or substitutes (g) | 0.0 | 7.6 |
| Total fat (% of energy) | 31.2 | 25.3 |
| Saturated fat (% of energy) | 9.6 | 7.1 |
| Monounsaturated fat (% of energy) | 11.7 | 9.4 |
| Polyunsaturated fat (% of energy) | 6.8 | 6.3 |
| Trans fat (% of energy) | 2.0 | 1.7 |
| Animal protein (g) | 45.8 | 46.3 |
| Fiber (g) | 20.0 | 24.8 |
| Sodium (mg) | 3028.4 | 3205.4 |
| Red meat (g) | 66.0 | 40.8 |
| Poultry (g) | 31.9 | 46.8 |
| Fish (g) | 15.4 | 19.1 |
| Dairy products (g) | 290.0 | 319.3 |
| Nuts/legumes (g) | 9.8 | 11.8 |
| Fruit (g) | 341.9 | 437.5 |
| Vegetables (g) | 291.7 | 364.5 |
| Potatoes (g) | 47.0 | 49.0 |
| Whole grains (g) | 26.6 | 35.1 |
| Refined grains (g) | 127.1 | 140.5 |
| Coffee (g) | 806.8 | 756.6 |
| Sugar-sweetened beverages (g) | 480.8 | 497.3 |
| Healthy Eating Index score | 67.9 | 73.3 |

BMI, body mass index.

^a^ Values are medians or percentages unless stated otherwise.

^b^ Household income in 1999.

^c^ Dietary intakes were energy-adjusted using the nutrient density method of 2000 kcal per day unless stated otherwise.

# Table D. Spearman correlations between egg or cholesterol and other dietary factors

|  | **Whole eggs** | **Cholesterol** | **Egg whites or substitutes** |
| --- | --- | --- | --- |
| **Whole eggs** | 1.00^a^ | 0.72 | -0.55 |
| **Cholesterol** | 0.72 | 1.00 | -0.34 |
| **Egg white or substitutes** | -0.55 | -0.34 | 1.00 |
| **Total fat** | 0.34 | 0.58 | -0.24 |
| **Saturated fat** | 0.36 | 0.64 | -0.29 |
| **Monounsaturated fat** | 0.34 | 0.56 | -0.24 |
| **Polyunsaturated fat** | 0.17 | 0.26 | -0.09 |
| **Trans fat** | 0.22 | 0.35 | -0.15 |
| **Animal protein** | 0.12 | 0.55 | 0.01 |
| **Fiber** | -0.25 | -0.37 | 0.21 |
| **Sodium** | 0.01 | 0.22 | 0.12 |
| **Red meat** | 0.26 | 0.58 | -0.21 |
| **Processed red meat** | 0.26 | 0.42 | -0.17 |
| **Unprocessed red meat** | 0.24 | 0.55 | -0.19 |
| **Poultry** | -0.09 | 0.19 | 0.14 |
| **Fish** | -0.01 | 0.19 | 0.08 |
| **Dairy** | -0.06 | -0.04 | 0.04 |
| **Nuts/legumes** | -0.04 | -0.06 | 0.06 |
| **Fruit** | -0.15 | -0.31 | 0.11 |
| **Vegetables** | -0.13 | -0.13 | 0.15 |
| **Potatoes** | 0.03 | 0.08 | 0.02 |
| **Whole grains** | -0.12 | -0.22 | 0.13 |
| **Refined grains** | -0.09 | -0.09 | 0.10 |
| **Coffee** | 0.09 | 0.07 | -0.01 |
| **Sugar-sweetened beverages** | -0.04 | -0.08 | 0.01 |
| **Healthy Eating Index score** | -0.20 | -0.37 | 0.20 |

^a^ Spearman correlations were calculated using energy-adjusted intakes.

# Table E. Associations of egg whites/substitutes consumption with all-cause, CVD, and cancer mortality

|  | **Egg whites/substitutes (g/2000 kcal/day)** | |  |
| --- | --- | --- | --- |
|  | **Non-consumers** | **Consumers** | **P** |
| Median intake (IQR) | 0 | 7.6 (3.4 to 15.7) |  |
| **Total mortality** |  |  |  |
| No. of deaths/participants | 111,191/447,570 | 18,137/73,550 |  |
| Person-years | 6,272,751 | 1,034,347 |  |
| Model 1^a^ | 1.00 | 0.91 (0.90 to 0.93) | <0.001 |
| Model 2^b^ | 1.00 | 0.89 (0.87 to 0.90) | <0.001 |
| Model 3^c^ | 1.00 | 0.91 (0.89 to 0.94) | <0.001 |
| Model 4^d^ | 1.00 | 0.92 (0.90 to 0.94) | <0.001 |
| Model 5^e^ | 1.00 | 0.94 (0.92 to 0.96) | <0.001 |
| Model 6^f^ | 1.00 | 0.93 (0.91 to 0.95) | <0.001 |
| **CVD mortality** |  |  |  |
| No. of deaths/participants | 32,383/447,570 | 6,364/73,550 |  |
| Person-years | 6,272,751 | 1,034,347 |  |
| Model 1^a^ | 1.00 | 1.08 (1.05 to 1.11) | <0.001 |
| Model 2^b^ | 1.00 | 0.94 (0.92 to 0.97) | <0.001 |
| Model 3^c^ | 1.00 | 0.97 (0.93 to 1.01) | 0.16 |
| Model 4^d^ | 1.00 | 0.97 (0.93 to 1.01) | 0.19 |
| Model 5^e^ | 1.00 | 0.99 (0.95 to 1.04) | 0.74 |
| Model 6^f^ | 1.00 | 0.98 (0.94 to 1.02) | 0.40 |
| **Cancer mortality** |  |  |  |
| No. of deaths/participants | 40,077/447,570 | 5,706/73,550 |  |
| Person-years | 6,272,751 | 1,034,347 |  |
| Model 1^a^ | 1.00 | 0.81 (0.79 to 0.83) | <0.001 |
| Model 2^b^ | 1.00 | 0.86 (0.84 to 0.89) | <0.001 |
| Model 3^c^ | 1.00 | 0.92 (0.88 to 0.96) | <0.001 |
| Model 4^d^ | 1.00 | 0.93 (0.89 to 0.96) | <0.001 |
| Model 5^e^ | 1.00 | 0.94 (0.90 to 0.98) | 0.004 |
| Model 6^f^ | 1.00 | 0.94 (0.90 to 0.98) | 0.005 |

CI, confidence interval; CVD, cardiovascular disease; HR, hazard ratio.

^a^ Model 1: adjusted for age and sex.

^b^ Model 2: adjusted for model 1 + BMI (in kg/m^2^; <18.5, 18.5 to 25, 25 to 30, 30 to 35, ≥35, or missing), race (white, black, Hispanic/Asian/Pacific Islander/American Indian/Alaskan native, or unknown/missing), education (less than high school, high school graduate, some college, college graduate, or unknown/missing), marital status (married/living as married or widowed/divorced/separated/never married/unknown), household income (quintiles), smoking (never smoked, quit, ≤20 cigarettes a day, quit, >20 cigarettes a day, currently smoking, ≤20 cigarettes a day, currently smoking, >20 cigarettes a day or unknown), alcohol (0, 0.1-4.9, 5.0-29.9, ≥30 g/day), vigorous physical activity (never/rarely, 1-3 times/mo, 1-2 times/wk, 3-4 times/wk, ≥5 times/wk, or unknown/missing), usual activity at work (sit all day, sit much of the day/walk sometimes, stand/walk often/no lifting, lift/carry light loads, and carry heavy loads), history of hypertension (yes or no), history of high cholesterol level (yes or no), history of heart disease (yes or no), stroke (yes or no), diabetes (yes or no), and cancer (yes or no) at baseline.

^c^ Model 3: adjusted for model 2 + total energy and whole eggs.

^d^ Model 4: adjusted for model 3 + dietary cholesterol.

^e^ Model 5: adjusted for model 3 + red meat, fish, poultry, and dairy products, fruit, vegetables, potatoes, nuts/legumes, whole grains, refined grains, coffee, and sugar sweetened beverages.

^f^ Model 6: adjusted for model 3 + Healthy Eating Index-2015.

# Table F. Data source of Figure 1. Multivariable-adjusted HRs (95% CIs) of all-cause and cause-specific mortality according to egg and cholesterol consumption

|  | **Whole eggs** | |  | **Egg whites or substitutes** | |  | **Cholesterol** | |
| --- | --- | --- | --- | --- | --- | --- | --- | --- |
|  | **HR (95% CI)** | **P** |  | **HR (95% CI)** | **P** |  | **HR (95% CI)** | **P** |
| **All causes of death** | 1.07 (1.06-1.08) | <0.001 |  | 0.93 (0.91-0.95) | <0.001 |  | 1.19 (1.16-1.22) | <0.001 |
| **Cardiovascular disease** | 1.07 (1.06-1.09) | <0.001 |  | 0.98 (0.94-1.02) | 0.40 |  | 1.16 (1.11-1.21) | <0.001 |
| **Heart disease** | 1.07 (1.06-1.09) | <0.001 |  | 1.00 (0.96-1.05) | 0.99 |  | 1.16 (1.11-1.22) | <0.001 |
| **Stroke** | 1.06 (1.02-1.10) | 0.007 |  | 0.88 (0.79-0.99) | 0.03 |  | 1.11 (0.99-1.25) | 0.07 |
| **Cancer** | 1.07 (1.06-1.09) | <0.001 |  | 0.94 (0.90-0.98) | 0.005 |  | 1.24 (1.19-1.29) | <0.001 |
| **Respiratory disease** | 1.14 (1.11-1.17) | <0.001 |  | 0.83 (0.75-0.91) | <0.001 |  | 1.32 (1.23-1.42) | <0.001 |
| **Alzheimer's disease** | 0.91 (0.84-0.99) | 0.02 |  | 0.68 (0.58-0.81) | <0.001 |  | 0.74 (0.59-0.93) | 0.01 |
| **Diabetes** | 1.07 (1.03-1.12) | 0.002 |  | 1.06 (0.92-1.22) | 0.42 |  | 1.16 (1.03-1.31) | 0.02 |
| **Infections** | 1.02 (0.96-1.08) | 0.52 |  | 0.98 (0.83-1.16) | 0.80 |  | 1.11 (0.95-1.31) | 0.19 |
| **Kidney disease** | 1.08 (1.01-1.14) | 0.02 |  | 1.01 (0.83-1.23) | 0.89 |  | 1.20 (1.00-1.43) | 0.05 |
| **Chronic liver disease** | 1.05 (0.96-1.14) | 0.28 |  | 0.69 (0.52-0.93) | 0.01 |  | 1.43 (1.13-1.80) | 0.003 |
| **Other causes** | 1.05 (1.03-1.07) | <0.001 |  | 0.86 (0.82-0.91) | <0.001 |  | 1.13 (1.07-1.20) | <0.001 |

CI, confidence interval; HR, hazard ratio. Values are HRs (95% CIs) of total and cause-specific mortality associated with each additional half a whole egg/day, egg white/substitutes consumption (consumers vs. non-consumers), or each additional 300 mg cholesterol/day. HRs were adjusted for age, sex, BMI, race, education, marital status, household income, smoking, alcohol, vigorous physical activity, usual activity at work, hypertension, hypercholesteremia, history of heart disease, stroke, diabetes, and cancer at baseline, total energy intake, and Healthy Eating Index-2015 (for eggs), or intakes of saturated fat, polyunsaturated fat, monounsaturated fat, trans fat, animal protein, fiber, and sodium (for cholesterol). Whole egg and egg whites/substitutes were mutually adjusted in their respective models.

# Table G. Multivariable-adjusted HRs (95% CIs) of heart disease and stroke mortality

|  | **Categories of egg or cholesterol consumption**^a^ | | | | |  |
| --- | --- | --- | --- | --- | --- | --- |
|  | **C1** | **C2** | **C3** | **C4** | **C5** | **P for trend** |
| **Heart disease mortality** |  |  |  |  |  |  |
| **Whole egg** |  |  |  |  |  |  |
| No of deaths/participants | 7,258/104,224 | 5,649/104,224 | 5,953/104,224 | 6,408/104,224 | 7,874/104,224 |  |
| Multivariable-adjusted HR (95% CI)^b^ | 1.00 | 1.01 (0.97-1.05) | 1.02 (0.98-1.05) | 1.08 (1.04-1.12) | 1.15 (1.11-1.20) | <0.001 |
| **Cholesterol** |  |  |  |  |  |  |
| No of deaths/participants | 6,094/104,224 | 6,112/104,224 | 6,135/104,224 | 6,772/104,224 | 8,029/104,224 |  |
| Multivariable-adjusted HR (95% CI)^c^ | 1.00 | 1.00 (0.96-1.04) | 1.00 (0.96-1.05) | 1.07 (1.02-1.11) | 1.13 (1.08-1.19) | <0.001 |
| **Egg whites or substitutes** |  |  |  |  |  |  |
| No of deaths/participants | 27,616/447,570 | 5,526/73,550 |  |  |  |  |
| Multivariable-adjusted HR (95% CI)^d^ | 1.00 | 1.00 (0.96-1.05) |  |  |  | 0.99 |
| **Stroke mortality** |  |  |  |  |  |  |
| **Whole egg** |  |  |  |  |  |  |
| No of deaths/participants | 1,197/104,224 | 1,034/104,224 | 1,041/104,224 | 1,084/104,224 | 1,249/104,224 |  |
| Multivariable-adjusted HR (95% CI)^b^ | 1.00 | 0.98 (0.90-1.08) | 0.97 (0.89-1.07) | 1.01 (0.92-1.10) | 1.09 (1.00-1.19) | 0.008 |
| **Cholesterol** |  |  |  |  |  |  |
| No of deaths/participants | 1,112/104,224 | 1,111/104,224 | 1,083/104,224 | 1,100/104,224 | 1,199/104,224 |  |
| Multivariable-adjusted HR (95% CI)^c^ | 1.00 | 1.02 (0.93-1.12) | 1.02 (0.92-1.12) | 1.04 (0.93-1.15) | 1.09 (0.97-1.23) | 0.12 |
| **Egg whites or substitutes** |  |  |  |  |  |  |
| No of deaths/participants | 4,767/447,570 | 838/73,550 |  |  |  |  |
| Multivariable-adjusted HR (95% CI)^d^ | 1.00 | 0.88 (0.79-0.99) |  |  |  | 0.03 |

^a^ Categories are quintiles for whole egg and cholesterol and consumers (C2) vs. non-consumers (C1) for egg whites/substitutes.

^b^ Adjusted for age, sex, BMI, race, education, marital status, household income, smoking, alcohol, vigorous physical activity, usual activity at work, hypertension, hypercholesteremia, history of heart disease, stroke, diabetes, and cancer at baseline, total energy, egg whites/substitutes and HEI-2015.

^c^ Adjusted for age, sex, BMI, race, education, marital status, household income, smoking, alcohol, vigorous physical activity, usual activity at work, hypertension, hypercholesteremia, history of heart disease, stroke, diabetes, and cancer at baseline, total energy and intakes of saturated fat, polyunsaturated fat, monounsaturated fat, trans fat, animal protein, fiber, and sodium.

^d^ Adjusted for age, sex, BMI, race, education, marital status, household income, smoking, alcohol, vigorous physical activity, usual activity at work, hypertension, hypercholesteremia, history of heart disease, stroke, diabetes, and cancer at baseline, total energy, whole egg and HEI-2015.

# Table H. Results of mediation analysis between whole egg and cholesterol for all-cause, CVD, and cancer mortality

| **Mediator** | **Proportion (%) of effect due to mediation (95% CI)** | **P value** |
| --- | --- | --- |
| **Cholesterol** |  |  |
| All-cause mortality | 63.2 (49.6 to 75.0) | <0.001 |
| CVD mortality | 62.3 (39.5 to 80.7) | <0.001 |
| Cancer mortality | 49.6 (31.9 to 67.4) | <0.001 |
| **Saturated fat** |  |  |
| Total mortality | 23.5 (19.2-28.4) | <0.001 |
| Cardiovascular mortality | 19.7 (13.2-28.3) | <0.001 |
| Cancer mortality | 16.3 (10.9-23.5) | <0.001 |
| **Animal protein** |  |  |
| Total mortality | 4.6 (3.2-6.5) | <0.001 |
| Cardiovascular mortality | 7.5 (5.0-11.1) | <0.001 |
| Cancer mortality | 1.2 (0.2-8.7) | 0.163 |

CI, confidence interval; CVD, cardiovascular disease.

# Table I. Multivariable-adjusted HRs (95% CIs) of mortality from non-CVD and non-cancer causes according to whole egg consumption

|  | **Quintiles of whole egg consumption** | | | | |  |
| --- | --- | --- | --- | --- | --- | --- |
|  | **Q1** | **Q2** | **Q3** | **Q4** | **Q5** | **P for trend** |
| **Respiratory disease** |  |  |  |  |  |  |
| Death cases/n | 1,487/104,224 | 1,754/104,224 | 1,882/104,224 | 1,962/104,224 | 2,714/104,224 |  |
| Multivariable-adjusted HR (95% CI) | 1.00 | 1.19 (1.10-1.28) | 1.26 (1.16-1.35) | 1.29 (1.20-1.39) | 1.52 (1.42-1.64) | <0.001 |
| **Alzheimer's disease** |  |  |  |  |  |  |
| Death cases/n | 490/104,224 | 419/104,224 | 394/104,224 | 396/104,224 | 347/104,224 |  |
| Multivariable-adjusted HR (95% CI) | 1.00 | 0.87 (0.76-1.00) | 0.83 (0.72-0.96) | 0.84 (0.73-0.98) | 0.75 (0.65-0.88) | 0.001 |
| **Diabetes** |  |  |  |  |  |  |
| Death cases/n | 757/104,224 | 516/104,224 | 581/104,224 | 650/104,224 | 1,008/104,224 |  |
| Multivariable-adjusted HR (95% CI) | 1.00 | 1.00 (0.89-1.13) | 1.03 (0.91-1.15) | 1.08 (0.96-1.21) | 1.20 (1.08-1.34) | <0.001 |
| **Infections** |  |  |  |  |  |  |
| Death cases/n | 505/104,224 | 477/104,224 | 491/104,224 | 507/104,224 | 589/104,224 |  |
| Multivariable-adjusted HR (95% CI) | 1.00 | 1.03 (0.90-1.18) | 1.03 (0.90-1.18) | 1.08 (0.94-1.24) | 1.06 (0.93-1.21) | 0.36 |
| **Kidney disease** |  |  |  |  |  |  |
| Death cases/n | 378/104,224 | 307/104,224 | 336/104,224 | 368/104,224 | 458/104,224 |  |
| Multivariable-adjusted HR (95% CI) | 1.00 | 1.04 (0.88-1.22) | 1.06 (0.91-1.25) | 1.14 (0.97-1.33) | 1.15 (0.99-1.35) | 0.04 |
| **Chronic liver disease** |  |  |  |  |  |  |
| Death cases/n | 157/104,224 | 209/104,224 | 200/104,224 | 223/104,224 | 258/104,224 |  |
| Multivariable-adjusted HR (95% CI) | 1.00 | 1.16 (0.92-1.46) | 1.03 (0.82-1.30) | 1.17 (0.94-1.47) | 1.13 (0.90-1.41) | 0.52 |
| **Other causes** |  |  |  |  |  |  |
| Death cases/n | 5,013/104,224 | 4,441/104,224 | 4,764/104,224 | 4,565/104,224 | 5,195/104,224 |  |
| Multivariable-adjusted HR (95% CI) | 1.00 | 0.96 (0.92-1.00) | 1.01 (0.97-1.05) | 0.97 (0.92-1.01) | 1.02 (0.98-1.07) | 0.06 |

CI, confidence interval; HR, hazard ratio. HRs were adjusted for age, sex, BMI, race, education, marital status, household income, smoking, alcohol, vigorous physical activity, usual activity at work, hypertension, hypercholesteremia, history of heart disease, stroke, diabetes, and cancer at baseline, total energy, egg whites/substitutes and HEI-2015.

# Table J. Multivariable-adjusted HRs (95% CIs) of mortality from non-CVD and non-cancer causes according to cholesterol consumption

|  | **Quintiles of cholesterol consumption** | | | | |  |
| --- | --- | --- | --- | --- | --- | --- |
|  | **Q1** | **Q2** | **Q3** | **Q4** | **Q5** | **P for trend** |
| **Respiratory disease** |  |  |  |  |  |  |
| Death cases/n | 1,463/104,224 | 1,653/104,224 | 1,830/104,224 | 2,109/104,224 | 2,744/104,224 |  |
| Multivariable-adjusted HR (95% CI) | 1.00 | 1.04 (0.96-1.12) | 1.10 (1.02-1.19) | 1.19 (1.09-1.29) | 1.33 (1.22-1.46) | <0.001 |
| **Alzheimer's disease** |  |  |  |  |  |  |
| Death cases/n | 511/104,224 | 479/104,224 | 378/104,224 | 355/104,224 | 323/104,224 |  |
| Multivariable-adjusted HR (95% CI) | 1.00 | 0.96 (0.84-1.10) | 0.78 (0.67-0.91) | 0.76 (0.64-0.90) | 0.71 (0.58-0.87) | <0.001 |
| **Diabetes** |  |  |  |  |  |  |
| Death cases/n | 497/104,224 | 537/104,224 | 628/104,224 | 769/104,224 | 1,081/104,224 |  |
| Multivariable-adjusted HR (95% CI) | 1.00 | 0.97 (0.85-1.10) | 1.09 (0.96-1.24) | 1.17 (1.02-1.34) | 1.28 (1.10-1.48) | <0.001 |
| **Infections** |  |  |  |  |  |  |
| Death cases/n | 480/104,224 | 442/104,224 | 512/104,224 | 499/104,224 | 636/104,224 |  |
| Multivariable-adjusted HR (95% CI) | 1.00 | 0.90 (0.78-1.03) | 1.01 (0.88-1.17) | 0.94 (0.81-1.10) | 1.07 (0.90-1.27) | 0.17 |
| **Kidney disease** |  |  |  |  |  |  |
| Death cases/n | 308/104,224 | 352/104,224 | 341/104,224 | 368/104,224 | 478/104,224 |  |
| Multivariable-adjusted HR (95% CI) | 1.00 | 1.09 (0.93-1.29) | 1.04 (0.87-1.24) | 1.06 (0.88-1.28) | 1.19 (0.97-1.46) | 0.12 |
| **Chronic liver disease** |  |  |  |  |  |  |
| Death cases/n | 191/104,224 | 196/104,224 | 177/104,224 | 228/104,224 | 255/104,224 |  |
| Multivariable-adjusted HR (95% CI) | 1.00 | 1.10 (0.89-1.36) | 1.01 (0.80-1.27) | 1.26 (0.98-1.60) | 1.28 (0.97-1.68) | 0.04 |
| **Other causes** |  |  |  |  |  |  |
| Death cases/n | 4,684/104,224 | 4,593/104,224 | 4,682/104,224 | 4,758/104,224 | 5,261/104,224 |  |
| Multivariable-adjusted HR (95% CI) | 1.00 | 0.97 (0.93-1.02) | 0.99 (0.95-1.04) | 0.99 (0.94-1.04) | 1.04 (0.98-1.10) | 0.07 |

CI, confidence interval; HR, hazard ratio. HRs were adjusted for age, sex, BMI, race, education, marital status, household income, smoking, alcohol, vigorous physical activity, usual activity at work, hypertension, hypercholesteremia, history of heart disease, stroke, diabetes, and cancer at baseline, total energy and intakes of saturated fat, polyunsaturated fat, monounsaturated fat, trans fat, animal protein, fiber, and sodium.

# Table K. Multivariable-adjusted HRs (95% CIs) of mortality from non-CVD and non-cancer causes according to egg whites/substitutes consumption

|  | **Egg whites or substitutes consumption** | |  |
| --- | --- | --- | --- |
|  | **Non-consumers** | **Consumers** | **P** |
| **Respiratory disease** |  |  |  |
| Death cases/n | 8,813/447,570 | 986/73,550 |  |
| Multivariable-adjusted HR (95% CI) | 1.00 | 0.83 (0.75-0.91) | <0.001 |
| **Alzheimer's disease** |  |  |  |
| Death cases/n | 1,745/447,570 | 301/73,550 |  |
| Multivariable-adjusted HR (95% CI) | 1.00 | 0.68 (0.58-0.81) | <0.001 |
| **Diabetes** |  |  |  |
| Death cases/n | 2,908/447,570 | 604/73,550 |  |
| Multivariable-adjusted HR (95% CI) | 1.00 | 1.06 (0.92-1.22) | 0.42 |
| **Infections** |  |  |  |
| Death cases/n | 2,205/447,570 | 364/73,550 |  |
| Multivariable-adjusted HR (95% CI) | 1.00 | 0.98 (0.83-1.16) | 0.80 |
| **Kidney disease** |  |  |  |
| Death cases/n | 1,561/447,570 | 286/73,550 |  |
| Multivariable-adjusted HR (95% CI) | 1.00 | 1.01 (0.83-1.23) | 0.89 |
| **Chronic liver disease** |  |  |  |
| Death cases/n | 952/447,570 | 95/73,550 |  |
| Multivariable-adjusted HR (95% CI) | 1.00 | 0.69 (0.52-0.93) | 0.01 |
| **Other causes** |  |  |  |
| Death cases/n | 20,547/447,570 | 3,431/73,550 |  |
| Multivariable-adjusted HR (95% CI) | 1.00 | 0.86 (0.82-0.91) | <0.001 |

CI, confidence interval; HR, hazard ratio. HRs were adjusted for age, sex, BMI, race, education, marital status, household income, smoking, alcohol, vigorous physical activity, usual activity at work, hypertension, hypercholesteremia, history of heart disease, stroke, diabetes, and cancer at baseline, total energy, whole egg and HEI-2015.

# Table L. Multivariable-adjusted HRs (95% CIs) of all-cause and cause-specific mortality according to fried eggs, non-fried eggs, egg whites, and egg substitutes consumption^a^

|  | **Fried eggs** | |  | **Non-fried eggs** | | **Egg whites** | |  | **Egg substitutes** | |
| --- | --- | --- | --- | --- | --- | --- | --- | --- | --- | --- |
|  | **HR (95% CI)^b^** | **P** |  | **HR (95% CI)^b^** | **P** | **HR (95% CI)**^c^ | **P** |  | **HR (95% CI)**^c^ | **P** |
| **All-cause** | 1.06 (1.05-1.07) | <0.001 |  | 1.06 (1.05-1.07) | <0.001 | 0.88 (0.85-0.91) | <0.001 |  | 0.96 (0.93-0.98) | <0.001 |
| **Cardiovascular disease** | 1.05 (1.03-1.07) | <0.001 |  | 1.08 (1.06-1.10) | <0.001 | 0.92 (0.87-0.98) | 0.007 |  | 1.01 (0.97-1.05) | 0.73 |
| **Cancer** | 1.08 (1.06-1.09) | <0.001 |  | 1.05 (1.03-1.07) | <0.001 | 0.89 (0.83-0.94) | <0.001 |  | 0.96 (0.92-1.00) | 0.05 |
| **Respiratory disease** | 1.12 (1.09-1.16) | <0.001 |  | 1.10 (1.07-1.14) | <0.001 | 0.73 (0.62-0.85) | <0.001 |  | 0.89 (0.81-0.98) | 0.02 |
| **Alzheimer's disease** | 0.86 (0.77-0.95) | 0.005 |  | 0.93 (0.85-1.01) | 0.10 | 0.71 (0.55-0.91) | 0.008 |  | 0.72 (0.60-0.86) | <0.001 |
| **Diabetes** | 1.05 (0.99-1.10) | 0.12 |  | 1.10 (1.05-1.15) | <0.001 | 1.00 (0.81-1.22) | 0.99 |  | 1.07 (0.93-1.23) | 0.32 |
| **Infections** | 1.00 (0.93-1.08) | 0.94 |  | 1.03 (0.96-1.10) | 0.45 | 0.84 (0.65-1.09) | 0.19 |  | 1.06 (0.90-1.25) | 0.51 |
| **Kidney disease** | 1.08 (1.00-1.16) | 0.05 |  | 1.07 (1.00-1.15) | 0.06 | 0.89 (0.66-1.19) | 0.42 |  | 1.06 (0.87-1.28) | 0.58 |
| **Chronic liver disease** | 1.01 (0.91-1.13) | 0.81 |  | 1.02 (0.92-1.13) | 0.75 | 0.66 (0.41-1.08) | 0.10 |  | 0.71 (0.52-0.96) | 0.03 |
| **Other causes** | 1.02 (1.00-1.05) | 0.07 |  | 1.05 (1.03-1.08) | <0.001 | 0.85 (0.78-0.92) | <0.001 |  | 0.88 (0.83-0.93) | <0.001 |

CI, confidence interval; HR, hazard ratio.

^a^ Values are multivariable HRs (CIs) of total and cause-specific mortality for fried egg, non-fried egg (each increment of half an egg), egg whites, and egg substitutes (consumers vs. non-consumers).

^b^ HRs were adjusted for age, sex, BMI, race, education, marital status, household income, smoking, alcohol, vigorous physical activity, usual activity at work, hypertension, hypercholesteremia, history of heart disease, stroke, diabetes, and cancer at baseline, total energy, egg whites/substitutes, HEI-2015, and non-fried egg (for fried egg), or fried egg (for non-fried egg).

^c^ HRs were adjusted for age, sex, BMI, race, education, marital status, household income, smoking, alcohol, vigorous physical activity, usual activity at work, hypertension, hypercholesteremia, history of heart disease, stroke, diabetes, and cancer at baseline, total energy, whole egg, HEI-2015, and egg substitutes (for egg whites), or egg whites (for egg substitutes).

# Table M. Multivariable-adjusted HRs (95% CIs) of mortality according to whole egg consumption based on convenient cutoffs

|  | **Whole egg consumption** | | | | | |  |
| --- | --- | --- | --- | --- | --- | --- | --- |
|  | **<1 egg/month** | **1-3 eggs/month** | **1-2 eggs/week** | **3-4 eggs/week** | **5-6 eggs/week** | **≥1 egg/day** | **P for trend** |
| **All-cause** |  |  |  |  |  |  |  |
| Death cases/n | 36,078/154,805 | 25,503/111,897 | 37,242/152,573 | 20,955/73,584 | 5,911/18,134 | 3,639/10,127 |  |
| Multivariable-adjusted HR (95% CI) | 1.00 | 1.04 (1.02-1.06) | 1.07 (1.05-1.09) | 1.12 (1.10-1.14) | 1.19 (1.16-1.22) | 1.25 (1.20-1.29) | <0.001 |
| **Cardiovascular disease** |  |  |  |  |  |  |  |
| Death cases/n | 11,506/154,805 | 7,195/111,897 | 10,873/152,573 | 6,315/73,584 | 1,764/18,134 | 1,094/10,127 |  |
| Multivariable-adjusted HR (95% CI) | 1.00 | 1.01 (0.98-1.04) | 1.06 (1.03-1.09) | 1.13 (1.09-1.17) | 1.19 (1.13-1.25) | 1.25 (1.17-1.33) | <0.001 |
| **Cancer** |  |  |  |  |  |  |  |
| Death cases/n | 11,506/154,805 | 7,195/111,897 | 10,873/152,573 | 6,315/73,584 | 1,764/18,134 | 1,094/10,127 |  |
| Multivariable-adjusted HR (95% CI) | 1.00 | 1.09 (1.06-1.12) | 1.10 (1.08-1.13) | 1.15 (1.11-1.18) | 1.18 (1.13-1.24) | 1.27 (1.19-1.35) | <0.001 |
| **Respiratory disease** |  |  |  |  |  |  |  |
| Death cases/n | 2,295/154,805 | 1,961/111,897 | 2,814/152,573 | 1,843/73,584 | 553/18,134 | 333/10,127 |  |
| Multivariable-adjusted HR (95% CI) | 1.00 | 1.16 (1.09-1.24) | 1.21 (1.14-1.29) | 1.43 (1.34-1.53) | 1.54 (1.40-1.70) | 1.49 (1.33-1.68) | <0.001 |
| **Alzheimer's disease** |  |  |  |  |  |  |  |
| Death cases/n | 741/154,805 | 377/111,897 | 588/152,573 | 238/73,584 | 56/18,134 | 46/10,127 |  |
| Multivariable-adjusted HR (95% CI) | 1.00 | 0.73 (0.64-0.83) | 0.83 (0.74-0.93) | 0.73 (0.62-0.85) | 0.74 (0.56-0.97) | 0.98 (0.72-1.33) | 0.002 |
| **Diabetes** |  |  |  |  |  |  |  |
| Death cases/n | 975/154,805 | 577/111,897 | 957/152,573 | 662/73,584 | 212/18,134 | 129/10,127 |  |
| Multivariable-adjusted HR (95% CI) | 1.00 | 1.04 (0.94-1.16) | 1.13 (1.02-1.24) | 1.22 (1.10-1.36) | 1.33 (1.14-1.56) | 1.22 (1.01-1.48) | <0.001 |
| **Infections** |  |  |  |  |  |  |  |
| Death cases/n | 733/154,805 | 498/111,897 | 747/152,573 | 401/73,584 | 115/18,134 | 75/10,127 |  |
| Multivariable-adjusted HR (95% CI) | 1.00 | 0.99 (0.88-1.12) | 1.06 (0.95-1.18) | 1.02 (0.90-1.17) | 1.08 (0.88-1.33) | 1.16 (0.91-1.48) | 0.20 |
| **Kidney disease** |  |  |  |  |  |  |  |
| Death cases/n | 523/154,805 | 338/111,897 | 517/152,573 | 317/73,584 | 93/18,134 | 59/10,127 |  |
| Multivariable-adjusted HR (95% CI) | 1.00 | 1.03 (0.89-1.19) | 1.09 (0.95-1.24) | 1.17 (1.01-1.36) | 1.25 (0.99-1.57) | 1.25 (0.94-1.65) | 0.008 |
|  |  |  |  |  |  |  |  |
| **Chronic liver disease** |  |  |  |  |  |  |  |
| Death cases/n | 248/154,805 | 216/111,897 | 308/152,573 | 185/73,584 | 59/18,134 | 31/10,127 |  |
| Multivariable-adjusted HR (95% CI) | 1.00 | 1.06 (0.87-1.28) | 1.05 (0.88-1.25) | 1.06 (0.87-1.31) | 1.18 (0.87-1.59) | 1.06 (0.72-1.56) | 0.42 |
| **Other causes** |  |  |  |  |  |  |  |
| Death cases/n | 7,155/154,805 | 4,795/111,897 | 6,816/152,573 | 3,544/73,584 | 1029/18,134 | 639/10,127 |  |
| Multivariable-adjusted HR (95% CI) | 1.00 | 0.98 (0.94-1.02) | 0.99 (0.96-1.03) | 0.99 (0.95-1.04) | 1.12 (1.04-1.19) | 1.17 (1.08-1.27) | 0.005 |

CI, confidence interval; HR, hazard ratio. HRs were adjusted for age, sex, BMI, race, education, marital status, household income, smoking, alcohol, vigorous physical activity, usual activity at work, hypertension, hypercholesteremia, history of heart disease, stroke, diabetes, and cancer at baseline, total energy, egg whites/substitutes and HEI-2015.

# Table N. Multivariable-adjusted HRs (95% CIs) of mortality according to cholesterol intake based on convenient cutoffs

|  | **Cholesterol consumption (mg/2000 kcal/day)** | | | | |  |
| --- | --- | --- | --- | --- | --- | --- |
|  | **<100** | **≥100 and <200** | **≥200 and <300** | **≥300 and <400** | **≥400** | **P for trend** |
| **All-cause** |  |  |  |  |  |  |
| Death cases/n | 6,565/29,564 | 48,087/210,170 | 50,198/200,698 | 1,7902/61,581 | 6,576/19,107 |  |
| Multivariable-adjusted HR (95% CI) | 1.00 | 1.02 (0.99-1.05) | 1.07 (1.04-1.10) | 1.15 (1.11-1.19) | 1.25 (1.20-1.30) | <0.001 |
| **Cardiovascular disease** |  |  |  |  |  |  |
| Death cases/n | 2,024/29,564 | 14,533/210,170 | 14,815/200,698 | 5,409/61,581 | 1,966/19,107 |  |
| Multivariable-adjusted HR (95% CI) | 1.00 | 1.02 (0.97-1.07) | 1.06 (1.00-1.12) | 1.16 (1.08-1.23) | 1.21 (1.12-1.30) | <0.001 |
| **Cancer** |  |  |  |  |  |  |
| Death cases/n | 2,219/29,564 | 16,997/210,170 | 18,035/200,698 | 6,299/61,581 | 2,233/19,107 |  |
| Multivariable-adjusted HR (95% CI) | 1.00 | 1.05 (1.00-1.10) | 1.13 (1.07-1.19) | 1.22 (1.15-1.30) | 1.34 (1.24-1.44) | <0.001 |
| **Respiratory disease** |  |  |  |  |  |  |
| Death cases/n | 371/29,564 | 3,277/210,170 | 3,953/200,698 | 1,584/61,581 | 614/19,107 |  |
| Multivariable-adjusted HR (95% CI) | 1.00 | 1.11 (0.99-1.25) | 1.24 (1.10-1.41) | 1.41 (1.23-1.62) | 1.52 (1.30-1.78) | <0.001 |
| **Alzheimer's disease** |  |  |  |  |  |  |
| Death cases/n | 166/29,564 | 932/210,170 | 694/200,698 | 192/61,581 | 62/19,107 |  |
| Multivariable-adjusted HR (95% CI) | 1.00 | 0.81 (0.68-0.98) | 0.68 (0.55-0.85) | 0.64 (0.49-0.85) | 0.66 (0.46-0.94) | 0.001 |
| **Diabetes** |  |  |  |  |  |  |
| Death cases/n | 130/29,564 | 1,080/210,170 | 1,435/200,698 | 620/61,581 | 247/19,107 |  |
| Multivariable-adjusted HR (95% CI) | 1.00 | 1.05 (0.86-1.27) | 1.22 (0.99-1.49) | 1.34 (1.07-1.68) | 1.32 (1.01-1.71) | <0.001 |
| **Infections** |  |  |  |  |  |  |
| Death cases/n | 126/29,564 | 948/210,170 | 984/200,698 | 372/61,581 | 139/19,107 |  |
| Multivariable-adjusted HR (95% CI) | 1.00 | 1.06 (0.87-1.30) | 1.10 (0.88-1.37) | 1.24 (0.96-1.60) | 1.33 (0.99-1.80) | 0.02 |
| **Kidney disease** |  |  |  |  |  |  |
| Death cases/n | 74/29,564 | 689/210,170 | 698/200,698 | 275/61,581 | 111/19,107 |  |
| Multivariable-adjusted HR (95% CI) | 1.00 | 1.28 (0.99-1.65) | 1.27 (0.96-1.68) | 1.44 (1.05-1.96) | 1.59 (1.11-2.27) | 0.03 |
|  |  |  |  |  |  |  |
| **Chronic liver disease** |  |  |  |  |  |  |
| Death cases/n | 56/29,564 | 381/210,170 | 400/200,698 | 149/61,581 | 61/19,107 |  |
| Multivariable-adjusted HR (95% CI) | 1.00 | 1.12 (0.83-1.52) | 1.24 (0.88-1.73) | 1.39 (0.94-2.05) | 1.67 (1.06-2.64) | 0.01 |
| **Other causes** |  |  |  |  |  |  |
| Death cases/n | 1,399/29,564 | 9,250/210,170 | 9,184/200,698 | 3,002/61,581 | 1,143/19,107 |  |
| Multivariable-adjusted HR (95% CI) | 1.00 | 0.93 (0.87-0.98) | 0.95 (0.88-1.01) | 0.96 (0.89-1.04) | 1.11 (1.01-1.23) | 0.003 |

CI, confidence interval; HR, hazard ratio. HRs were adjusted for age, sex, BMI, race, education, marital status, household income, smoking, alcohol, vigorous physical activity, usual activity at work, hypertension, hypercholesteremia, history of heart disease, stroke, diabetes, and cancer at baseline, total energy and intakes of saturated fat, polyunsaturated fat, monounsaturated fat, trans fat, animal protein, fiber, and sodium.

# Table O. Sensitivity analyses for the associations of egg and cholesterol consumption with mortality by excluding participants with extreme BMIs

|  | **Categories of egg or cholesterol consumption** | | | | |  |
| --- | --- | --- | --- | --- | --- | --- |
|  | **C1** | **C2** | **C3** | **C4** | **C5** | **P for trend** |
| **All-cause** |  |  |  |  |  |  |
| Whole egg^a^ | 1.00 | 1.02 (1.00-1.04) | 1.06 (1.04-1.08) | 1.08 (1.06-1.10) | 1.14 (1.12-1.16) | <0.001 |
| Cholesterol^b^ | 1.00 | 1.01 (0.99-1.03) | 1.03 (1.01-1.05) | 1.06 (1.04-1.09) | 1.14 (1.11-1.17) | <0.001 |
| Egg whites/substitutes^c^ | 1.00 | 0.93 (0.90-0.95) |  |  |  | <0.001 |
| **Cardiovascular disease** |  |  |  |  |  |  |
| Whole egg^a^ | 1.00 | 1.00 (0.97-1.04) | 1.00 (0.97-1.04) | 1.07 (1.03-1.11) | 1.14 (1.10-1.18) | <0.001 |
| Cholesterol^b^ | 1.00 | 1.01 (0.98-1.05) | 1.01 (0.97-1.05) | 1.07 (1.02-1.11) | 1.13 (1.08-1.18) | <0.001 |
| Egg whites/substitutes^c^ | 1.00 | 0.98 (0.93-1.02) |  |  |  | 0.26 |
| **Cancer** |  |  |  |  |  |  |
| Whole egg^a^ | 1.00 | 1.05 (1.02-1.09) | 1.12 (1.08-1.15) | 1.12 (1.08-1.16) | 1.16 (1.13-1.20) | <0.001 |
| Cholesterol^b^ | 1.00 | 1.03 (0.99-1.06) | 1.08 (1.05-1.12) | 1.10 (1.06-1.14) | 1.19 (1.14-1.24) | <0.001 |
| Egg whites/substitutes^c^ | 1.00 | 0.95 (0.91-0.99) |  |  |  | 0.01 |
| **Respiratory disease** |  |  |  |  |  |  |
| Whole egg^a^ | 1.00 | 1.20 (1.11-1.30) | 1.24 (1.15-1.35) | 1.31 (1.21-1.41) | 1.50 (1.39-1.62) | <0.001 |
| Cholesterol^b^ | 1.00 | 1.03 (0.95-1.11) | 1.09 (1.00-1.18) | 1.15 (1.06-1.26) | 1.30 (1.18-1.43) | <0.001 |
| Egg whites/substitutes^c^ | 1.00 | 0.83 (0.75-0.91) |  |  |  | <0.001 |
| **Alzheimer's disease** |  |  |  |  |  |  |
| Whole egg^a^ | 1.00 | 0.84 (0.72-0.97) | 0.77 (0.66-0.89) | 0.83 (0.72-0.97) | 0.70 (0.60-0.82) | <0.001 |
| Cholesterol^b^ | 1.00 | 0.95 (0.82-1.09) | 0.78 (0.67-0.92) | 0.75 (0.63-0.90) | 0.69 (0.56-0.85) | <0.001 |
| Egg whites/substitutes^c^ | 1.00 | 0.69 (0.58-0.82) |  |  |  | <0.001 |
| **Diabetes** |  |  |  |  |  |  |
| Whole egg^a^ | 1.00 | 0.99 (0.87-1.13) | 1.03 (0.91-1.17) | 1.10 (0.97-1.24) | 1.21 (1.08-1.36) | <0.001 |
| Cholesterol^b^ | 1.00 | 1.00 (0.87-1.14) | 1.10 (0.96-1.26) | 1.21 (1.04-1.39) | 1.32 (1.13-1.55) | <0.001 |
| Egg whites/substitutes^c^ | 1.00 | 1.08 (0.93-1.25) |  |  |  | 0.32 |
|  |  |  |  |  |  |  |
| **Infections** |  |  |  |  |  |  |
| Whole egg^a^ | 1.00 | 1.06 (0.92-1.22) | 1.06 (0.92-1.22) | 1.09 (0.94-1.26) | 1.07 (0.93-1.24) | 0.45 |
| Cholesterol^b^ | 1.00 | 0.89 (0.77-1.03) | 1.01 (0.87-1.17) | 0.93 (0.79-1.09) | 1.06 (0.88-1.27) | 0.25 |
| Egg whites/substitutes^c^ | 1.00 | 0.97 (0.82-1.16) |  |  |  | 0.77 |
| **Kidney disease** |  |  |  |  |  |  |
| Whole egg^a^ | 1.00 | 1.02 (0.86-1.21) | 1.10 (0.93-1.31) | 1.17 (0.99-1.39) | 1.19 (1.02-1.40) | 0.01 |
| Cholesterol^b^ | 1.00 | 1.11 (0.94-1.32) | 1.07 (0.89-1.28) | 1.14 (0.94-1.38) | 1.28 (1.03-1.59) | 0.02 |
| Egg whites/substitutes^c^ | 1.00 | 1.08 (0.88-1.32) |  |  |  | 0.49 |
| **Chronic liver disease** |  |  |  |  |  |  |
| Whole egg^a^ | 1.00 | 1.13 (0.89-1.43) | 1.06 (0.84-1.35) | 1.15 (0.91-1.46) | 1.14 (0.90-1.43) | 0.42 |
| Cholesterol^b^ | 1.00 | 1.07 (0.86-1.33) | 0.99 (0.78-1.26) | 1.24 (0.97-1.60) | 1.25 (0.94-1.65) | 0.07 |
| Egg whites/substitutes^c^ | 1.00 | 0.68 (0.50-0.92) |  |  |  | 0.01 |
| **Other causes** |  |  |  |  |  |  |
| Whole egg^a^ | 1.00 | 0.95 (0.91-1.00) | 1.01 (0.96-1.05) | 0.97 (0.92-1.01) | 1.01 (0.96-1.05) | 0.20 |
| Cholesterol^b^ | 1.00 | 0.98 (0.93-1.02) | 0.98 (0.94-1.03) | 0.99 (0.93-1.04) | 1.03 (0.97-1.09) | 0.11 |
| Egg whites/substitutes^c^ | 1.00 | 0.85 (0.80-0.90) |  |  |  | <0.001 |

Data are multivariable HRs (95% CIs) for whole egg and cholesterol intakes (quintiles) and egg whites/substitutes consumption (consumers vs. non-consumers).

^a^ HRs were adjusted for age, sex, BMI, race, education, marital status, household income, smoking, alcohol, vigorous physical activity, usual activity at work, hypertension, hypercholesteremia, history of heart disease, stroke, diabetes, and cancer at baseline, total energy, egg whites/substitutes and HEI-2015.

^b^ HRs were adjusted for age, sex, BMI, race, education, marital status, household income, smoking, alcohol, vigorous physical activity, usual activity at work, hypertension, hypercholesteremia, history of heart disease, stroke, diabetes, and cancer at baseline, total energy and intakes of saturated fat, polyunsaturated fat, monounsaturated fat, trans fat, animal protein, fiber, and sodium.

^c^ HRs were adjusted for age, sex, BMI, race, education, marital status, household income, smoking, alcohol, vigorous physical activity, usual activity at work, hypertension, hypercholesteremia, history of heart disease, stroke, diabetes, and cancer at baseline, total energy, whole egg and HEI-2015.

# Table P. Sensitivity analyses for the associations of egg and cholesterol consumption with mortality by further adjusting for perceived health condition

|  | **Categories of egg or cholesterol consumption** | | | | |  |
| --- | --- | --- | --- | --- | --- | --- |
|  | **C1** | **C2** | **C3** | **C4** | **C5** | **P for trend** |
| **All-cause** |  |  |  |  |  |  |
| Whole egg^a^ | 1.00 | 1.02 (1.00-1.04) | 1.05 (1.03-1.07) | 1.06 (1.04-1.08) | 1.13 (1.11-1.15) | <0.001 |
| Cholesterol^b^ | 1.00 | 1.00 (0.98-1.02) | 1.02 (1.00-1.04) | 1.05 (1.03-1.07) | 1.12 (1.09-1.15) | <0.001 |
| Egg whites/substitutes^c^ | 1.00 | 0.94 (0.92-0.96) |  |  |  | <0.001 |
| **Cardiovascular disease** |  |  |  |  |  |  |
| Whole egg^a^ | 1.00 | 1.00 (0.97-1.04) | 1.00 (0.97-1.04) | 1.06 (1.03-1.10) | 1.13 (1.10-1.17) | <0.001 |
| Cholesterol^b^ | 1.00 | 1.00 (0.96-1.03) | 1.00 (0.96-1.04) | 1.05 (1.01-1.09) | 1.11 (1.06-1.16) | <0.001 |
| Egg whites/substitutes^c^ | 1.00 | 0.99 (0.95-1.03) |  |  |  | 0.49 |
| **Cancer** |  |  |  |  |  |  |
| Whole egg^a^ | 1.00 | 1.05 (1.01-1.08) | 1.10 (1.07-1.14) | 1.10 (1.07-1.14) | 1.15 (1.11-1.19) | <0.001 |
| Cholesterol^b^ | 1.00 | 1.02 (0.99-1.06) | 1.07 (1.04-1.11) | 1.08 (1.04-1.12) | 1.17 (1.12-1.22) | <0.001 |
| Egg whites/substitutes^c^ | 1.00 | 0.95 (0.91-0.99) |  |  |  | 0.02 |
| **Respiratory disease** |  |  |  |  |  |  |
| Whole egg^a^ | 1.00 | 1.17 (1.08-1.26) | 1.23 (1.14-1.32) | 1.25 (1.16-1.35) | 1.47 (1.37-1.58) | <0.001 |
| Cholesterol^b^ | 1.00 | 1.02 (0.95-1.10) | 1.07 (0.99-1.16) | 1.15 (1.06-1.25) | 1.28 (1.17-1.41) | <0.001 |
| Egg whites/substitutes^c^ | 1.00 | 0.85 (0.78-0.94) |  |  |  | 0.001 |
| **Alzheimer's disease** |  |  |  |  |  |  |
| Whole egg^a^ | 1.00 | 0.87 (0.75-1.00) | 0.83 (0.72-0.96) | 0.84 (0.73-0.97) | 0.75 (0.65-0.88) | 0.002 |
| Cholesterol^b^ | 1.00 | 0.96 (0.84-1.10) | 0.78 (0.67-0.91) | 0.75 (0.63-0.90) | 0.71 (0.58-0.86) | <0.001 |
| Egg whites/substitutes^c^ | 1.00 | 0.68 (0.58-0.81) |  |  |  | <0.001 |
| **Diabetes** |  |  |  |  |  |  |
| Whole egg^a^ | 1.00 | 0.99 (0.88-1.12) | 1.02 (0.90-1.15) | 1.07 (0.96-1.21) | 1.19 (1.07-1.32) | <0.001 |
| Cholesterol^b^ | 1.00 | 0.97 (0.85-1.10) | 1.08 (0.95-1.23) | 1.16 (1.02-1.33) | 1.26 (1.09-1.47) | <0.001 |
| Egg whites/substitutes^c^ | 1.00 | 1.07 (0.93-1.23) |  |  |  | 0.37 |
|  |  |  |  |  |  |  |
| **Infections** |  |  |  |  |  |  |
| Whole egg^a^ | 1.00 | 1.02 (0.89-1.16) | 1.01 (0.88-1.16) | 1.06 (0.92-1.21) | 1.04 (0.91-1.18) | 0.55 |
| Cholesterol^b^ | 1.00 | 0.89 (0.77-1.02) | 1.00 (0.87-1.15) | 0.92 (0.79-1.08) | 1.04 (0.88-1.24) | 0.28 |
| Egg whites/substitutes^c^ | 1.00 | 1.00 (0.84-1.18) |  |  |  | 0.96 |
| **Kidney disease** |  |  |  |  |  |  |
| Whole egg^a^ | 1.00 | 1.03 (0.87-1.21) | 1.06 (0.90-1.24) | 1.13 (0.96-1.32) | 1.14 (0.98-1.33) | 0.07 |
| Cholesterol^b^ | 1.00 | 1.09 (0.92-1.28) | 1.03 (0.86-1.22) | 1.04 (0.86-1.25) | 1.16 (0.95-1.43) | 0.18 |
| Egg whites/substitutes^c^ | 1.00 | 1.02 (0.84-1.24) |  |  |  | 0.83 |
| **Chronic liver disease** |  |  |  |  |  |  |
| Whole egg^a^ | 1.00 | 1.12 (0.89-1.41) | 0.99 (0.78-1.24) | 1.12 (0.89-1.40) | 1.06 (0.85-1.32) | 0.86 |
| Cholesterol^b^ | 1.00 | 1.07 (0.87-1.33) | 0.97 (0.77-1.23) | 1.19 (0.93-1.52) | 1.20 (0.91-1.58) | 0.12 |
| Egg whites/substitutes^c^ | 1.00 | 0.73 (0.54-0.98) |  |  |  | 0.03 |
| **Other causes** |  |  |  |  |  |  |
| Whole egg^a^ | 1.00 | 0.95 (0.91-0.99) | 1.00 (0.95-1.04) | 0.95 (0.91-1.00) | 1.00 (0.96-1.05) | 0.21 |
| Cholesterol^b^ | 1.00 | 0.97 (0.93-1.01) | 0.98 (0.94-1.03) | 0.98 (0.93-1.03) | 1.02 (0.96-1.08) | 0.23 |
| Egg whites/substitutes^c^ | 1.00 | 0.87 (0.83-0.92) |  |  |  | <0.001 |

Data are multivariable HRs (95% CIs) for whole egg and cholesterol intakes (quintiles) and egg whites/substitutes consumption (consumers vs. non-consumers).

^a^ HRs were adjusted for age, sex, BMI, race, education, marital status, household income, smoking, alcohol, vigorous physical activity, usual activity at work, hypertension, hypercholesteremia, perceived health condition (excellent, very good, good, fair or poor), history of heart disease, stroke, diabetes, and cancer at baseline, total energy, egg whites/substitutes and HEI-2015.

^b^ HRs were adjusted for age, sex, BMI, race, education, marital status, household income, smoking, alcohol, vigorous physical activity, usual activity at work, hypertension, hypercholesteremia, perceived health condition (excellent, very good, good, fair or poor), history of heart disease, stroke, diabetes, and cancer at baseline, total energy and intakes of saturated fat, polyunsaturated fat, monounsaturated fat, trans fat, animal protein, fiber, and sodium.

^c^ HRs were adjusted for age, sex, BMI, race, education, marital status, household income, smoking, alcohol, vigorous physical activity, usual activity at work, hypertension, hypercholesteremia, perceived health condition (excellent, very good, good, fair or poor), history of heart disease, stroke, diabetes, and cancer at baseline, total energy, whole egg and HEI-2015.

# Table Q. Sensitivity analyses for the associations of egg and cholesterol consumption with mortality by further adjusting for a propensity score

|  | **Categories of egg or cholesterol consumption** | | | | |  |
| --- | --- | --- | --- | --- | --- | --- |
|  | **C1** | **C2** | **C3** | **C4** | **C5** | **P for trend** |
| **All-cause** |  |  |  |  |  |  |
| Whole egg^a^ | 1.00 | 0.99 (0.97-1.01) | 1.03 (1.00-1.05) | 1.04 (1.02-1.06) | 1.11 (1.09-1.13) | <0.001 |
| Cholesterol^b^ | 1.00 | 1.02 (1.00-1.05) | 1.05 (1.03-1.08) | 1.08 (1.05-1.11) | 1.14 (1.12-1.17) | <0.001 |
| Egg whites/substitutes^c^ | 1.00 | 0.93 (0.91-0.95) |  |  |  | <0.001 |
| **Cardiovascular disease** |  |  |  |  |  |  |
| Whole egg^a^ | 1.00 | 1.00 (0.96-1.04) | 1.00 (0.96-1.04) | 1.06 (1.02-1.10) | 1.14 (1.09-1.18) | <0.001 |
| Cholesterol^b^ | 1.00 | 1.02 (0.98-1.06) | 1.03 (0.99-1.07) | 1.08 (1.03-1.12) | 1.13 (1.08-1.18) | <0.001 |
| Egg whites/substitutes^c^ | 1.00 | 0.99 (0.95-1.04) |  |  |  | 0.68 |
| **Cancer** |  |  |  |  |  |  |
| Whole egg^a^ | 1.00 | 1.03 (0.99-1.07) | 1.08 (1.04-1.13) | 1.08 (1.04-1.12) | 1.14 (1.09-1.18) | <0.001 |
| Cholesterol^b^ | 1.00 | 1.05 (1.01-1.09) | 1.11 (1.07-1.15) | 1.12 (1.08-1.17) | 1.20 (1.15-1.25) | <0.001 |
| Egg whites/substitutes^c^ | 1.00 | 0.95 (0.91-0.99) |  |  |  | 0.01 |
| **Respiratory disease** |  |  |  |  |  |  |
| Whole egg^a^ | 1.00 | 1.09 (1.00-1.19) | 1.16 (1.06-1.26) | 1.18 (1.09-1.29) | 1.40 (1.29-1.52) | <0.001 |
| Cholesterol^b^ | 1.00 | 1.04 (0.96-1.13) | 1.10 (1.01-1.20) | 1.19 (1.09-1.30) | 1.33 (1.21-1.46) | <0.001 |
| Egg whites/substitutes^c^ | 1.00 | 0.81 (0.73-0.89) |  |  |  | <0.001 |
| **Alzheimer's disease** |  |  |  |  |  |  |
| Whole egg^a^ | 1.00 | 0.76 (0.65-0.89) | 0.72 (0.61-0.84) | 0.73 (0.62-0.86) | 0.65 (0.55-0.77) | <0.001 |
| Cholesterol^b^ | 1.00 | 0.98 (0.85-1.13) | 0.80 (0.68-0.94) | 0.76 (0.64-0.91) | 0.70 (0.57-0.86) | <0.001 |
| Egg whites/substitutes^c^ | 1.00 | 0.71 (0.60-0.85) |  |  |  | <0.001 |
| **Diabetes** |  |  |  |  |  |  |
| Whole egg^a^ | 1.00 | 1.04 (0.90-1.20) | 1.05 (0.91-1.20) | 1.12 (0.97-1.28) | 1.25 (1.09-1.43) | <0.001 |
| Cholesterol^b^ | 1.00 | 0.96 (0.84-1.11) | 1.07 (0.93-1.24) | 1.15 (0.99-1.34) | 1.25 (1.07-1.46) | <0.001 |
| Egg whites/substitutes^c^ | 1.00 | 1.06 (0.92-1.22) |  |  |  | 0.43 |
|  |  |  |  |  |  |  |
| **Infections** |  |  |  |  |  |  |
| Whole egg^a^ | 1.00 | 1.01 (0.87-1.19) | 1.01 (0.87-1.19) | 1.06 (0.91-1.24) | 1.05 (0.90-1.23) | 0.40 |
| Cholesterol^b^ | 1.00 | 0.93 (0.80-1.07) | 1.05 (0.90-1.23) | 0.98 (0.83-1.16) | 1.09 (0.92-1.30) | 0.16 |
| Egg whites/substitutes^c^ | 1.00 | 1.00 (0.84-1.19) |  |  |  | 0.99 |
| **Kidney disease** |  |  |  |  |  |  |
| Whole egg^a^ | 1.00 | 1.07 (0.88-1.30) | 1.11 (0.92-1.34) | 1.18 (0.97-1.42) | 1.21 (1.00-1.46) | 0.03 |
| Cholesterol^b^ | 1.00 | 1.08 (0.91-1.29) | 1.03 (0.85-1.24) | 1.05 (0.86-1.28) | 1.18 (0.96-1.45) | 0.13 |
| Egg whites/substitutes^c^ | 1.00 | 1.07 (0.87-1.30) |  |  |  | 0.53 |
| **Chronic liver disease** |  |  |  |  |  |  |
| Whole egg^a^ | 1.00 | 1.13 (0.87-1.46) | 1.01 (0.78-1.31) | 1.14 (0.88-1.48) | 1.10 (0.85-1.42) | 0.64 |
| Cholesterol^b^ | 1.00 | 1.19 (0.95-1.50) | 1.10 (0.85-1.42) | 1.34 (1.03-1.74) | 1.30 (0.98-1.73) | 0.08 |
| Egg whites/substitutes^c^ | 1.00 | 0.66 (0.48-0.89) |  |  |  | 0.007 |
| **Other causes** |  |  |  |  |  |  |
| Whole egg^a^ | 1.00 | 0.90 (0.86-0.95) | 0.95 (0.91-1.00) | 0.91 (0.87-0.96) | 0.97 (0.92-1.02) | 0.26 |
| Cholesterol^b^ | 1.00 | 0.99 (0.94-1.03) | 1.00 (0.95-1.05) | 1.00 (0.95-1.05) | 1.04 (0.98-1.10) | 0.11 |
| Egg whites/substitutes^c^ | 1.00 | 0.86 (0.81-0.91) |  |  |  | <0.001 |

Data are multivariable HRs (95% CIs) for whole egg and cholesterol intakes (quintiles) and egg whites/substitutes consumption (consumers vs. non-consumers).

^a^ HRs were adjusted for propensity score, age, sex, BMI, race, education, marital status, household income, smoking, alcohol, vigorous physical activity, usual activity at work, hypertension, hypercholesteremia, history of heart disease, stroke, diabetes, and cancer at baseline, total energy, egg whites/substitutes and HEI-2015.

^b^ HRs were adjusted for propensity score, age, sex, BMI, race, education, marital status, household income, smoking, alcohol, vigorous physical activity, usual activity at work, hypertension, hypercholesteremia, history of heart disease, stroke, diabetes, and cancer at baseline, total energy and intakes of saturated fat, polyunsaturated fat, monounsaturated fat, trans fat, animal protein, fiber, and sodium.

^c^ HRs were adjusted for propensity score, age, sex, BMI, race, education, marital status, household income, smoking, alcohol, vigorous physical activity, usual activity at work, hypertension, hypercholesteremia, history of heart disease, stroke, diabetes, and cancer at baseline, total energy, whole egg and HEI-2015.

# Table R. Sensitivity analyses for the associations of egg and cholesterol consumption with mortality by further adjusting for the use of vitamin supplements and aspirin

|  | **Categories of egg or cholesterol consumption** | | | | |  |
| --- | --- | --- | --- | --- | --- | --- |
|  | **C1** | **C2** | **C3** | **C4** | **C5** | **P for trend** |
| **All-cause** |  |  |  |  |  |  |
| Whole egg^a^ | 1.00 | 1.03 (1.01-1.05) | 1.07 (1.05-1.09) | 1.08 (1.06-1.10) | 1.15 (1.13-1.17) | <0.001 |
| Cholesterol^b^ | 1.00 | 1.01 (0.99-1.03) | 1.04 (1.01-1.06) | 1.07 (1.04-1.09) | 1.14 (1.12-1.17) | <0.001 |
| Egg whites/substitutes^c^ | 1.00 | 0.93 (0.91-0.95) |  |  |  | <0.001 |
| **Cardiovascular disease** |  |  |  |  |  |  |
| Whole egg^a^ | 1.00 | 1.01 (0.98-1.05) | 1.02 (0.98-1.06) | 1.08 (1.04-1.12) | 1.16 (1.12-1.20) | <0.001 |
| Cholesterol^b^ | 1.00 | 1.01 (0.97-1.04) | 1.01 (0.97-1.05) | 1.07 (1.03-1.11) | 1.13 (1.08-1.19) | <0.001 |
| Egg whites/substitutes^c^ | 1.00 | 0.98 (0.94-1.02) |  |  |  | 0.26 |
| **Cancer** |  |  |  |  |  |  |
| Whole egg^a^ | 1.00 | 1.06 (1.02-1.09) | 1.12 (1.08-1.15) | 1.12 (1.08-1.15) | 1.17 (1.13-1.21) | <0.001 |
| Cholesterol^b^ | 1.00 | 1.03 (0.99-1.06) | 1.08 (1.05-1.12) | 1.10 (1.06-1.14) | 1.19 (1.14-1.24) | <0.001 |
| Egg whites/substitutes^c^ | 1.00 | 0.94 (0.90-0.98) |  |  |  | 0.006 |
| **Respiratory disease** |  |  |  |  |  |  |
| Whole egg^a^ | 1.00 | 1.20 (1.11-1.29) | 1.26 (1.17-1.36) | 1.30 (1.20-1.40) | 1.53 (1.43-1.64) | <0.001 |
| Cholesterol^b^ | 1.00 | 1.04 (0.97-1.12) | 1.10 (1.02-1.19) | 1.19 (1.10-1.29) | 1.34 (1.22-1.46) | <0.001 |
| Egg whites/substitutes^c^ | 1.00 | 0.83 (0.75-0.91) |  |  |  | <0.001 |
| **Alzheimer's disease** |  |  |  |  |  |  |
| Whole egg^a^ | 1.00 | 0.87 (0.76-1.01) | 0.84 (0.73-0.97) | 0.85 (0.73-0.98) | 0.76 (0.65-0.88) | 0.002 |
| Cholesterol^b^ | 1.00 | 0.96 (0.84-1.10) | 0.78 (0.67-0.91) | 0.76 (0.64-0.90) | 0.71 (0.58-0.87) | <0.001 |
| Egg whites/substitutes^c^ | 1.00 | 0.68 (0.57-0.81) |  |  |  | <0.001 |
| **Diabetes** |  |  |  |  |  |  |
| Whole egg^a^ | 1.00 | 1.00 (0.89-1.13) | 1.03 (0.91-1.16) | 1.08 (0.96-1.22) | 1.20 (1.08-1.34) | <0.001 |
| Cholesterol^b^ | 1.00 | 0.97 (0.85-1.10) | 1.09 (0.96-1.24) | 1.18 (1.03-1.35) | 1.28 (1.10-1.48) | <0.001 |
| Egg whites/substitutes^c^ | 1.00 | 1.06 (0.92-1.22) |  |  |  | 0.43 |
|  |  |  |  |  |  |  |
| **Infections** |  |  |  |  |  |  |
| Whole egg^a^ | 1.00 | 1.04 (0.90-1.19) | 1.04 (0.90-1.19) | 1.09 (0.95-1.25) | 1.07 (0.94-1.23) | 0.31 |
| Cholesterol^b^ | 1.00 | 0.90 (0.78-1.03) | 1.02 (0.88-1.18) | 0.95 (0.81-1.11) | 1.08 (0.91-1.28) | 0.14 |
| Egg whites/substitutes^c^ | 1.00 | 0.97 (0.83-1.15) |  |  |  | 0.76 |
| **Kidney disease** |  |  |  |  |  |  |
| Whole egg^a^ | 1.00 | 1.04 (0.88-1.23) | 1.07 (0.91-1.26) | 1.15 (0.98-1.35) | 1.16 (1.00-1.36) | 0.04 |
| Cholesterol^b^ | 1.00 | 1.09 (0.93-1.29) | 1.04 (0.88-1.24) | 1.07 (0.88-1.28) | 1.19 (0.97-1.46) | 0.11 |
| Egg whites/substitutes^c^ | 1.00 | 1.01 (0.83-1.23) |  |  |  | 0.90 |
| **Chronic liver disease** |  |  |  |  |  |  |
| Whole egg^a^ | 1.00 | 1.16 (0.92-1.45) | 1.02 (0.81-1.29) | 1.17 (0.93-1.46) | 1.11 (0.89-1.39) | 0.57 |
| Cholesterol^b^ | 1.00 | 1.09 (0.88-1.35) | 1.00 (0.79-1.26) | 1.24 (0.98-1.59) | 1.26 (0.96-1.66) | 0.05 |
| Egg whites/substitutes^c^ | 1.00 | 0.70 (0.52-0.94) |  |  |  | 0.02 |
| **Other causes** |  |  |  |  |  |  |
| Whole egg^a^ | 1.00 | 0.96 (0.92-1.00) | 1.01 (0.97-1.06) | 0.97 (0.93-1.02) | 1.03 (0.98-1.07) | 0.05 |
| Cholesterol^b^ | 1.00 | 0.98 (0.93-1.02) | 0.99 (0.95-1.04) | 0.99 (0.94-1.05) | 1.04 (0.98-1.10) | 0.07 |
| Egg whites/substitutes^c^ | 1.00 | 0.86 (0.82-0.91) |  |  |  | <0.001 |

Data are multivariable HRs (95% CIs) for whole egg and cholesterol intakes (quintiles) and egg whites/substitutes consumption (consumers vs. non-consumers).

^a^ HRs were adjusted for age, sex, BMI, race, education, marital status, household income, smoking, alcohol, vigorous physical activity, usual activity at work, vitamin supplements use (yes or no), aspirin use (yes or no), hypertension, hypercholesteremia, history of heart disease, stroke, diabetes, and cancer at baseline, total energy, egg whites/substitutes and HEI-2015.

^b^ HRs were adjusted for age, sex, BMI, race, education, marital status, household income, smoking, alcohol, vigorous physical activity, usual activity at work, vitamin supplements use (yes or no), aspirin use (yes or no), hypertension, hypercholesteremia, history of heart disease, stroke, diabetes, and cancer at baseline, total energy and intakes of saturated fat, polyunsaturated fat, monounsaturated fat, trans fat, animal protein, fiber, and sodium.

^c^ HRs were adjusted for age, sex, BMI, race, education, marital status, household income, smoking, alcohol, vigorous physical activity, usual activity at work, vitamin supplements use (yes or no), aspirin use (yes or no), hypertension, hypercholesteremia, history of heart disease, stroke, diabetes, and cancer at baseline, total energy, whole egg and HEI-2015.

# Table S. Sensitivity analyses for the associations of egg and cholesterol consumption with mortality by carefully adjusting for processed meat

|  | **Categories of egg or cholesterol consumption** | | | | |  |
| --- | --- | --- | --- | --- | --- | --- |
|  | **C1** | **C2** | **C3** | **C4** | **C5** | **P for trend** |
| **All-cause** |  |  |  |  |  |  |
| Whole egg^a^ | 1.00 | 1.02 (1.00-1.04) | 1.05 (1.03-1.07) | 1.07 (1.05-1.09) | 1.14 (1.12-1.16) | <0.001 |
| Cholesterol^b^ | 1.00 | 1.04 (1.02-1.06) | 1.09 (1.06-1.11) | 1.13 (1.11-1.16) | 1.23 (1.20-1.26) | <0.001 |
| Egg whites/substitutes^c^ | 1.00 | 0.94 (0.92-0.96) |  |  |  | <0.001 |
| **Cardiovascular disease** |  |  |  |  |  |  |
| Whole egg^a^ | 1.00 | 1.00 (0.97-1.04) | 1.00 (0.97-1.04) | 1.07 (1.03-1.10) | 1.15 (1.11-1.19) | <0.001 |
| Cholesterol^b^ | 1.00 | 1.03 (1.00-1.07) | 1.05 (1.01-1.09) | 1.12 (1.08-1.16) | 1.20 (1.16-1.25) | <0.001 |
| Egg whites/substitutes^c^ | 1.00 | 0.99 (0.95-1.04) |  |  |  | 0.73 |
| **Cancer** |  |  |  |  |  |  |
| Whole egg^a^ | 1.00 | 1.05 (1.01-1.08) | 1.10 (1.06-1.14) | 1.10 (1.06-1.14) | 1.15 (1.11-1.19) | <0.001 |
| Cholesterol^b^ | 1.00 | 1.05 (1.02-1.08) | 1.12 (1.08-1.15) | 1.14 (1.10-1.18) | 1.25 (1.20-1.29) | <0.001 |
| Egg whites/substitutes^c^ | 1.00 | 0.94 (0.90-0.98) |  |  |  | 0.004 |
| **Respiratory disease** |  |  |  |  |  |  |
| Whole egg^a^ | 1.00 | 1.16 (1.07-1.25) | 1.21 (1.12-1.30) | 1.24 (1.15-1.34) | 1.47 (1.37-1.59) | <0.001 |
| Cholesterol^b^ | 1.00 | 1.15 (1.07-1.24) | 1.28 (1.19-1.38) | 1.45 (1.35-1.56) | 1.69 (1.57-1.83) | <0.001 |
| Egg whites/substitutes^c^ | 1.00 | 0.85 (0.77-0.93) |  |  |  | <0.001 |
| **Alzheimer's disease** |  |  |  |  |  |  |
| Whole egg^a^ | 1.00 | 0.88 (0.76-1.01) | 0.84 (0.73-0.98) | 0.86 (0.74-1.00) | 0.78 (0.67-0.91) | 0.008 |
| Cholesterol^b^ | 1.00 | 1.01 (0.89-1.16) | 0.86 (0.74-0.99) | 0.86 (0.73-1.00) | 0.84 (0.70-0.99) | 0.01 |
| Egg whites/substitutes^c^ | 1.00 | 0.68 (0.57-0.81) |  |  |  | <0.001 |
| **Diabetes** |  |  |  |  |  |  |
| Whole egg^a^ | 1.00 | 0.99 (0.88-1.12) | 1.03 (0.91-1.16) | 1.09 (0.97-1.23) | 1.23 (1.10-1.37) | <0.001 |
| Cholesterol^b^ | 1.00 | 0.98 (0.87-1.11) | 1.10 (0.97-1.24) | 1.18 (1.04-1.34) | 1.27 (1.12-1.44) | <0.001 |
| Egg whites/substitutes^c^ | 1.00 | 1.10 (0.95-1.26) |  |  |  | 0.21 |
|  |  |  |  |  |  |  |
| **Infections** |  |  |  |  |  |  |
| Whole egg^a^ | 1.00 | 1.01 (0.88-1.16) | 1.01 (0.88-1.16) | 1.06 (0.93-1.22) | 1.06 (0.92-1.21) | 0.31 |
| Cholesterol^b^ | 1.00 | 0.93 (0.82-1.06) | 1.08 (0.94-1.23) | 1.02 (0.89-1.18) | 1.18 (1.02-1.37) | 0.005 |
| Egg whites/substitutes^c^ | 1.00 | 1.01 (0.86-1.20) |  |  |  | 0.89 |
| **Kidney disease** |  |  |  |  |  |  |
| Whole egg^a^ | 1.00 | 1.01 (0.86-1.19) | 1.04 (0.88-1.22) | 1.11 (0.95-1.30) | 1.14 (0.97-1.33) | 0.05 |
| Cholesterol^b^ | 1.00 | 1.11 (0.95-1.30) | 1.06 (0.90-1.26) | 1.09 (0.92-1.29) | 1.21 (1.02-1.45) | 0.05 |
| Egg whites/substitutes^c^ | 1.00 | 1.05 (0.86-1.27) |  |  |  | 0.64 |
| **Chronic liver disease** |  |  |  |  |  |  |
| Whole egg^a^ | 1.00 | 1.12 (0.89-1.40) | 1.00 (0.79-1.25) | 1.14 (0.91-1.43) | 1.12 (0.89-1.40) | 0.39 |
| Cholesterol^b^ | 1.00 | 1.06 (0.86-1.29) | 0.95 (0.76-1.18) | 1.16 (0.93-1.44) | 1.14 (0.90-1.43) | 0.18 |
| Egg whites/substitutes^c^ | 1.00 | 0.79 (0.59-1.05) |  |  |  | 0.10 |
| **Other causes** |  |  |  |  |  |  |
| Whole egg^a^ | 1.00 | 0.95 (0.91-1.00) | 1.01 (0.96-1.05) | 0.97 (0.93-1.01) | 1.03 (0.99-1.08) | 0.007 |
| Cholesterol^b^ | 1.00 | 1.01 (0.97-1.05) | 1.04 (1.00-1.09) | 1.06 (1.02-1.11) | 1.13 (1.08-1.19) | <0.001 |
| Egg whites/substitutes^c^ | 1.00 | 0.87 (0.83-0.92) |  |  |  | <0.001 |

Data are multivariable HRs (95% CIs) for whole egg and cholesterol intakes (quintiles) and egg whites/substitutes consumption (consumers vs. non-consumers).

^a^ HRs were adjusted for age, sex, BMI, race, education, marital status, household income, smoking, alcohol, vigorous physical activity, usual activity at work, hypertension, hypercholesteremia, history of heart disease, stroke, diabetes, and cancer at baseline, total energy, egg whites/substitutes, processed red meat, unprocessed red meat, processed white meat, unprocessed white meat, dairy products, fruit, vegetables, potatoes, nuts/legumes, whole grains, refined grains, coffee, and sugar sweetened beverages.

^b^ HRs were adjusted for age, sex, BMI, race, education, marital status, household income, smoking, alcohol, vigorous physical activity, usual activity at work, hypertension, hypercholesteremia, history of heart disease, stroke, diabetes, and cancer at baseline, total energy and intakes of processed red meat, unprocessed red meat, processed white meat, and unprocessed white meat.

^c^ HRs were adjusted for age, sex, BMI, race, education, marital status, household income, smoking, alcohol, vigorous physical activity, usual activity at work, hypertension, hypercholesteremia, history of heart disease, stroke, diabetes, and cancer at baseline, total energy, whole egg, processed red meat, unprocessed red meat, processed white meat, unprocessed white meat, dairy products, fruit, vegetables, potatoes, nuts/legumes, whole grains, refined grains, coffee, and sugar sweetened beverages.

# Table T. Sensitivity analyses for the associations of egg and cholesterol consumption with mortality by further adjusting for cholesterol-lowering medications (n=293,918)

|  | **Categories of egg or cholesterol consumption** | | | | |  |
| --- | --- | --- | --- | --- | --- | --- |
|  | **C1** | **C2** | **C3** | **C4** | **C5** | **P for trend** |
| **All-cause** |  |  |  |  |  |  |
| Whole egg^a^ | 1.00 | 1.00 (0.97-1.04) | 1.03 (1.00-1.07) | 1.03 (1.00-1.07) | 1.08 (1.04-1.11) | <0.001 |
| Cholesterol^b^ | 1.00 | 1.01 (0.98-1.05) | 1.01 (0.97-1.05) | 1.04 (1.00-1.08) | 1.09 (1.04-1.13) | <0.001 |
| Egg whites/substitutes^c^ | 1.00 | 0.94 (0.90-0.99) |  |  |  | 0.008 |
| **Cardiovascular disease** |  |  |  |  |  |  |
| Whole egg^a^ | 1.00 | 0.98 (0.92-1.04) | 1.00 (0.94-1.06) | 1.00 (0.94-1.07) | 1.08 (1.02-1.15) | <0.001 |
| Cholesterol^b^ | 1.00 | 0.99 (0.93-1.06) | 0.98 (0.92-1.05) | 1.01 (0.94-1.09) | 1.07 (0.98-1.16) | 0.048 |
| Egg whites/substitutes^c^ | 1.00 | 0.99 (0.91-1.07) |  |  |  | 0.76 |
| **Cancer** |  |  |  |  |  |  |
| Whole egg^a^ | 1.00 | 1.04 (0.98-1.10) | 1.06 (1.00-1.13) | 1.07 (1.01-1.13) | 1.09 (1.03-1.16) | 0.008 |
| Cholesterol^b^ | 1.00 | 1.03 (0.98-1.10) | 1.06 (0.99-1.12) | 1.10 (1.03-1.18) | 1.15 (1.07-1.24) | <0.001 |
| Egg whites/substitutes^c^ | 1.00 | 0.97 (0.90-1.05) |  |  |  | 0.42 |
| **Respiratory disease** |  |  |  |  |  |  |
| Whole egg^a^ | 1.00 | 1.10 (0.96-1.25) | 1.17 (1.03-1.34) | 1.15 (1.01-1.31) | 1.32 (1.17-1.49) | <0.001 |
| Cholesterol^b^ | 1.00 | 1.00 (0.88-1.14) | 1.03 (0.90-1.17) | 1.03 (0.89-1.19) | 1.16 (1.00-1.36) | 0.02 |
| Egg whites/substitutes^c^ | 1.00 | 0.89 (0.76-1.05) |  |  |  | 0.17 |
| **Alzheimer's disease** |  |  |  |  |  |  |
| Whole egg^a^ | 1.00 | 0.92 (0.70-1.20) | 0.97 (0.74-1.27) | 0.99 (0.76-1.29) | 0.89 (0.67-1.17) | 0.52 |
| Cholesterol^b^ | 1.00 | 1.03 (0.80-1.32) | 0.86 (0.65-1.15) | 0.87 (0.63-1.18) | 0.85 (0.59-1.21) | 0.25 |
| Egg whites/substitutes^c^ | 1.00 | 0.79 (0.57-1.09) |  |  |  | 0.15 |
| **Diabetes** |  |  |  |  |  |  |
| Whole egg^a^ | 1.00 | 0.83 (0.67-1.04) | 1.00 (0.81-1.23) | 1.00 (0.81-1.23) | 1.08 (0.89-1.31) | 0.07 |
| Cholesterol^b^ | 1.00 | 1.00 (0.80-1.25) | 1.00 (0.79-1.26) | 1.09 (0.85-1.39) | 1.17 (0.89-1.53) | 0.14 |
| Egg whites/substitutes^c^ | 1.00 | 1.00 (0.78-1.28) |  |  |  | 0.98 |
|  |  |  |  |  |  |  |
| **Infections** |  |  |  |  |  |  |
| Whole egg^a^ | 1.00 | 1.05 (0.83-1.34) | 0.92 (0.72-1.18) | 0.96 (0.75-1.23) | 0.97 (0.76-1.23) | 0.63 |
| Cholesterol^b^ | 1.00 | 1.01 (0.80-1.28) | 1.02 (0.79-1.31) | 1.02 (0.78-1.34) | 1.11 (0.82-1.51) | 0.46 |
| Egg whites/substitutes^c^ | 1.00 | 0.98 (0.72-1.33) |  |  |  | 0.91 |
| **Kidney disease** |  |  |  |  |  |  |
| Whole egg^a^ | 1.00 | 1.01 (0.77-1.33) | 0.89 (0.67-1.17) | 1.22 (0.94-1.59) | 1.09 (0.84-1.41) | 0.25 |
| Cholesterol^b^ | 1.00 | 0.97 (0.74-1.27) | 1.11 (0.84-1.47) | 0.98 (0.72-1.33) | 1.06 (0.75-1.49) | 0.76 |
| Egg whites/substitutes^c^ | 1.00 | 0.86 (0.62-1.20) |  |  |  | 0.37 |
| **Chronic liver disease** |  |  |  |  |  |  |
| Whole egg^a^ | 1.00 | 1.23 (0.79-1.91) | 1.12 (0.72-1.75) | 1.34 (0.87-2.06) | 1.19 (0.78-1.82) | 0.66 |
| Cholesterol^b^ | 1.00 | 1.41 (0.93-2.13) | 1.29 (0.83-2.02) | 1.37 (0.85-2.20) | 1.38 (0.82-2.33) | 0.47 |
| Egg whites/substitutes^c^ | 1.00 | 0.62 (0.35-1.09) |  |  |  | 0.10 |
| **Other causes** |  |  |  |  |  |  |
| Whole egg^a^ | 1.00 | 0.95 (0.88-1.03) | 1.00 (0.92-1.08) | 0.96 (0.88-1.03) | 0.98 (0.91-1.06) | 0.97 |
| Cholesterol^b^ | 1.00 | 1.01 (0.93-1.09) | 0.97 (0.90-1.06) | 1.00 (0.91-1.09) | 0.98 (0.89-1.09) | 0.73 |
| Egg whites/substitutes^c^ | 1.00 | 0.87 (0.79-0.96) |  |  |  | 0.004 |

Data are multivariable HRs (95% CIs) for whole egg and cholesterol intakes (quintiles) and egg whites/substitutes consumption (consumers vs. non-consumers).

^a^ HRs were adjusted for age, sex, BMI, race, education, marital status, household income, smoking, alcohol, vigorous physical activity, usual activity at work, cholesterol-lowering medications use (yes or no), hypertension, hypercholesteremia, history of heart disease, stroke, diabetes, and cancer at baseline, total energy, egg whites/substitutes and HEI-2015.

^b^ HRs were adjusted for age, sex, BMI, race, education, marital status, household income, smoking, alcohol, vigorous physical activity, usual activity at work, cholesterol-lowering medications use (yes or no), hypertension, hypercholesteremia, history of heart disease, stroke, diabetes, and cancer at baseline, total energy and intakes of saturated fat, polyunsaturated fat, monounsaturated fat, trans fat, animal protein, fiber, and sodium.

^c^ HRs were adjusted for age, sex, BMI, race, education, marital status, household income, smoking, alcohol, vigorous physical activity, usual activity at work, cholesterol-lowering medications use (yes or no), hypertension, hypercholesteremia, history of heart disease, stroke, diabetes, and cancer at baseline, total energy, whole egg and HEI-2015.

# Table U. Sensitivity analyses for the associations of egg and cholesterol consumption with mortality by excluding the initial 5 years of follow-up

|  | **Categories of egg or cholesterol consumption** | | | | |  |
| --- | --- | --- | --- | --- | --- | --- |
|  | **C1** | **C2** | **C3** | **C4** | **C5** | **P for trend** |
| **All-cause** |  |  |  |  |  |  |
| Whole egg^a^ | 1.00 | 1.01 (0.99-1.03) | 1.04 (1.01-1.06) | 1.05 (1.03-1.07) | 1.11 (1.09-1.13) | <0.001 |
| Cholesterol^b^ | 1.00 | 1.00 (0.97-1.02) | 1.01 (0.98-1.03) | 1.04 (1.01-1.06) | 1.10 (1.07-1.13) | <0.001 |
| Egg whites/substitutes^c^ | 1.00 | 0.93 (0.91-0.96) |  |  |  | <0.001 |
| **Cardiovascular disease** |  |  |  |  |  |  |
| Whole egg^a^ | 1.00 | 1.01 (0.97-1.05) | 1.00 (0.96-1.04) | 1.04 (1.00-1.08) | 1.12 (1.08-1.16) | <0.001 |
| Cholesterol^b^ | 1.00 | 0.98 (0.94-1.02) | 0.97 (0.93-1.02) | 1.03 (0.98-1.07) | 1.09 (1.03-1.14) | <0.001 |
| Egg whites/substitutes^c^ | 1.00 | 0.98 (0.93-1.02) |  |  |  | 0.30 |
| **Cancer** |  |  |  |  |  |  |
| Whole egg^a^ | 1.00 | 1.03 (0.99-1.07) | 1.08 (1.04-1.13) | 1.08 (1.04-1.12) | 1.13 (1.09-1.17) | <0.0001 |
| Cholesterol^b^ | 1.00 | 1.02 (0.98-1.06) | 1.06 (1.02-1.10) | 1.09 (1.04-1.13) | 1.16 (1.11-1.22) | <0.0001 |
| Egg whites/substitutes^c^ | 1.00 | 0.94 (0.89-0.98) |  |  |  | 0.007 |
| **Respiratory disease** |  |  |  |  |  |  |
| Whole egg^a^ | 1.00 | 1.18 (1.09-1.28) | 1.23 (1.14-1.34) | 1.26 (1.16-1.37) | 1.46 (1.35-1.58) | <0.001 |
| Cholesterol^b^ | 1.00 | 1.03 (0.95-1.12) | 1.09 (1.00-1.19) | 1.16 (1.06-1.27) | 1.31 (1.19-1.45) | <0.001 |
| Egg whites/substitutes^c^ | 1.00 | 0.87 (0.79-0.97) |  |  |  | 0.01 |
| **Alzheimer's disease** |  |  |  |  |  |  |
| Whole egg^a^ | 1.00 | 0.87 (0.76-1.01) | 0.83 (0.71-0.96) | 0.85 (0.73-0.98) | 0.75 (0.64-0.87) | 0.001 |
| Cholesterol^b^ | 1.00 | 0.96 (0.84-1.10) | 0.78 (0.67-0.91) | 0.75 (0.63-0.90) | 0.72 (0.59-0.88) | <0.001 |
| Egg whites/substitutes^c^ | 1.00 | 0.68 (0.57-0.80) |  |  |  | <0.001 |
| **Diabetes** |  |  |  |  |  |  |
| Whole egg^a^ | 1.00 | 0.96 (0.84-1.10) | 1.03 (0.90-1.17) | 1.05 (0.92-1.20) | 1.17 (1.04-1.31) | <0.001 |
| Cholesterol^b^ | 1.00 | 0.94 (0.82-1.09) | 1.05 (0.91-1.21) | 1.13 (0.97-1.31) | 1.20 (1.02-1.42) | 0.002 |
| Egg whites/substitutes^c^ | 1.00 | 1.13 (0.97-1.32) |  |  |  | 0.13 |
|  |  |  |  |  |  |  |
| **Infections** |  |  |  |  |  |  |
| Whole egg^a^ | 1.00 | 0.99 (0.85-1.14) | 1.00 (0.86-1.15) | 1.00 (0.86-1.16) | 1.01 (0.88-1.17) | 0.76 |
| Cholesterol^b^ | 1.00 | 0.89 (0.76-1.03) | 1.00 (0.86-1.17) | 0.92 (0.77-1.09) | 1.02 (0.84-1.23) | 0.52 |
| Egg whites/substitutes^c^ | 1.00 | 0.95 (0.79-1.14) |  |  |  | 0.56 |
| **Kidney disease** |  |  |  |  |  |  |
| Whole egg^a^ | 1.00 | 1.02 (0.86-1.22) | 1.05 (0.88-1.24) | 1.12 (0.95-1.33) | 1.09 (0.92-1.28) | 0.26 |
| Cholesterol^b^ | 1.00 | 1.17 (0.98-1.39) | 1.06 (0.88-1.28) | 1.08 (0.88-1.32) | 1.19 (0.96-1.49) | 0.25 |
| Egg whites/substitutes^c^ | 1.00 | 1.06 (0.86-1.31) |  |  |  | 0.59 |
| **Chronic liver disease** |  |  |  |  |  |  |
| Whole egg^a^ | 1.00 | 1.09 (0.84-1.42) | 0.98 (0.75-1.28) | 1.06 (0.81-1.37) | 1.04 (0.81-1.34) | 0.94 |
| Cholesterol^b^ | 1.00 | 1.00 (0.78-1.27) | 0.96 (0.73-1.25) | 1.09 (0.82-1.44) | 1.11 (0.81-1.53) | 0.34 |
| Egg whites/substitutes^c^ | 1.00 | 0.70 (0.50-0.98) |  |  |  | 0.04 |
| **Other causes** |  |  |  |  |  |  |
| Whole egg^a^ | 1.00 | 0.95 (0.90-1.00) | 0.98 (0.93-1.03) | 0.96 (0.91-1.01) | 0.99 (0.94-1.04) | 0.57 |
| Cholesterol^b^ | 1.00 | 0.97 (0.92-1.02) | 0.96 (0.91-1.01) | 0.96 (0.91-1.02) | 0.99 (0.93-1.06) | 0.79 |
| Egg whites/substitutes^c^ | 1.00 | 0.88 (0.82-0.93) |  |  |  | <0.001 |

Data are multivariable HRs (95% CIs) for whole egg and cholesterol intakes (quintiles) and egg whites/substitutes consumption (consumers vs. non-consumers).

^a^ HRs were adjusted for age, sex, BMI, race, education, marital status, household income, smoking, alcohol, vigorous physical activity, usual activity at work, hypertension, hypercholesteremia, history of heart disease, stroke, diabetes, and cancer at baseline, total energy, egg whites/substitutes and HEI-2015.

^b^ HRs were adjusted for age, sex, BMI, race, education, marital status, household income, smoking, alcohol, vigorous physical activity, usual activity at work, hypertension, hypercholesteremia, history of heart disease, stroke, diabetes, and cancer at baseline, total energy and intakes of saturated fat, polyunsaturated fat, monounsaturated fat, trans fat, animal protein, fiber, and sodium.

^c^ HRs were adjusted for age, sex, BMI, race, education, marital status, household income, smoking, alcohol, vigorous physical activity, usual activity at work, hypertension, hypercholesteremia, history of heart disease, stroke, diabetes, and cancer at baseline, total energy, whole egg and HEI-2015.

# Table V. Sensitivity analyses for the associations of egg and cholesterol consumption with mortality by excluding participants with CVD, cancer, or diabetes at baseline

|  | **Categories of egg or cholesterol consumption** | | | | |  |
| --- | --- | --- | --- | --- | --- | --- |
|  | **C1** | **C2** | **C3** | **C4** | **C5** | **P for trend** |
| **All-cause** |  |  |  |  |  |  |
| Whole egg^a^ | 1.00 | 0.98 (0.96-1.01) | 1.01 (0.99-1.04) | 1.02 (1.00-1.05) | 1.09 (1.06-1.12) | <0.001 |
| Cholesterol^b^ | 1.00 | 0.98 (0.95-1.00) | 1.00 (0.97-1.03) | 1.03 (1.00-1.06) | 1.10 (1.07-1.14) | <0.001 |
| Egg whites/substitutes^c^ | 1.00 | 0.89 (0.87-0.92) |  |  |  | <0.001 |
| **Cardiovascular disease** |  |  |  |  |  |  |
| Whole egg^a^ | 1.00 | 0.95 (0.90-1.00) | 0.96 (0.91-1.01) | 1.01 (0.96-1.07) | 1.08 (1.03-1.14) | <0.001 |
| Cholesterol^b^ | 1.00 | 0.96 (0.91-1.01) | 0.97 (0.91-1.02) | 1.02 (0.96-1.08) | 1.10 (1.03-1.18) | <0.001 |
| Egg whites/substitutes^c^ | 1.00 | 0.92 (0.86-0.98) |  |  |  | 0.01 |
| **Cancer** |  |  |  |  |  |  |
| Whole egg^a^ | 1.00 | 1.02 (0.97-1.06) | 1.06 (1.02-1.11) | 1.05 (1.01-1.10) | 1.11 (1.06-1.16) | <0.001 |
| Cholesterol^b^ | 1.00 | 0.99 (0.95-1.03) | 1.04 (0.99-1.09) | 1.06 (1.01-1.12) | 1.14 (1.08-1.20) | <0.001 |
| Egg whites/substitutes^c^ | 1.00 | 0.90 (0.85-0.95) |  |  |  | <0.001 |
| **Respiratory disease** |  |  |  |  |  |  |
| Whole egg^a^ | 1.00 | 1.16 (1.05-1.27) | 1.22 (1.11-1.34) | 1.25 (1.14-1.38) | 1.47 (1.35-1.61) | <0.001 |
| Cholesterol^b^ | 1.00 | 1.01 (0.92-1.11) | 1.07 (0.97-1.18) | 1.15 (1.03-1.27) | 1.28 (1.15-1.44) | <0.001 |
| Egg whites/substitutes^c^ | 1.00 | 0.84 (0.74-0.95) |  |  |  | 0.005 |
| **Alzheimer's disease** |  |  |  |  |  |  |
| Whole egg^a^ | 1.00 | 0.81 (0.69-0.96) | 0.79 (0.67-0.94) | 0.76 (0.64-0.91) | 0.73 (0.61-0.87) | 0.005 |
| Cholesterol^b^ | 1.00 | 0.98 (0.83-1.15) | 0.83 (0.69-1.00) | 0.79 (0.64-0.97) | 0.73 (0.58-0.93) | 0.004 |
| Egg whites/substitutes^c^ | 1.00 | 0.72 (0.59-0.88) |  |  |  | 0.002 |
| **Diabetes** |  |  |  |  |  |  |
| Whole egg^a^ | 1.00 | 0.97 (0.73-1.28) | 1.06 (0.80-1.39) | 1.24 (0.95-1.62) | 1.26 (0.97-1.63) | 0.02 |
| Cholesterol^b^ | 1.00 | 1.09 (0.81-1.46) | 1.28 (0.95-1.73) | 1.33 (0.97-1.83) | 1.60 (1.13-2.26) | 0.004 |
| Egg whites/substitutes^c^ | 1.00 | 1.00 (0.71-1.41) |  |  |  | 0.99 |
|  |  |  |  |  |  |  |
| **Infections** |  |  |  |  |  |  |
| Whole egg^a^ | 1.00 | 0.99 (0.83-1.19) | 0.99 (0.83-1.19) | 1.07 (0.89-1.28) | 1.03 (0.86-1.23) | 0.56 |
| Cholesterol^b^ | 1.00 | 0.89 (0.74-1.06) | 1.01 (0.84-1.23) | 0.94 (0.77-1.16) | 1.04 (0.83-1.32) | 0.43 |
| Egg whites/substitutes^c^ | 1.00 | 0.95 (0.76-1.19) |  |  |  | 0.65 |
| **Kidney disease** |  |  |  |  |  |  |
| Whole egg^a^ | 1.00 | 0.82 (0.64-1.06) | 0.91 (0.71-1.17) | 0.95 (0.74-1.21) | 0.96 (0.76-1.22) | 0.59 |
| Cholesterol^b^ | 1.00 | 1.05 (0.82-1.36) | 1.04 (0.79-1.37) | 1.11 (0.83-1.49) | 1.10 (0.80-1.53) | 0.55 |
| Egg whites/substitutes^c^ | 1.00 | 0.88 (0.65-1.19) |  |  |  | 0.42 |
| **Chronic liver disease** |  |  |  |  |  |  |
| Whole egg^a^ | 1.00 | 1.19 (0.89-1.57) | 0.95 (0.71-1.27) | 1.12 (0.84-1.49) | 1.01 (0.76-1.34) | 0.63 |
| Cholesterol^b^ | 1.00 | 1.09 (0.83-1.41) | 0.84 (0.62-1.14) | 1.12 (0.82-1.52) | 1.12 (0.79-1.58) | 0.42 |
| Egg whites/substitutes^c^ | 1.00 | 0.72 (0.49-1.06) |  |  |  | 0.09 |
| **Other causes** |  |  |  |  |  |  |
| Whole egg^a^ | 1.00 | 0.92 (0.87-0.97) | 0.97 (0.92-1.03) | 0.94 (0.89-1.00) | 0.99 (0.93-1.04) | 0.36 |
| Cholesterol^b^ | 1.00 | 0.96 (0.91-1.01) | 0.96 (0.91-1.02) | 0.95 (0.89-1.02) | 1.01 (0.94-1.09) | 0.38 |
| Egg whites/substitutes^c^ | 1.00 | 0.89 (0.83-0.95) |  |  |  | <0.001 |

Data are multivariable HRs (95% CIs) for whole egg and cholesterol intakes (quintiles) and egg whites/substitutes consumption (consumers vs. non-consumers).

^a^ HRs were adjusted for age, sex, BMI, race, education, marital status, household income, smoking, alcohol, vigorous physical activity, usual activity at work, hypertension, hypercholesteremia, history of heart disease, stroke, diabetes, and cancer at baseline, total energy, egg whites/substitutes and HEI-2015.

^b^ HRs were adjusted for age, sex, BMI, race, education, marital status, household income, smoking, alcohol, vigorous physical activity, usual activity at work, hypertension, hypercholesteremia, history of heart disease, stroke, diabetes, and cancer at baseline, total energy and intakes of saturated fat, polyunsaturated fat, monounsaturated fat, trans fat, animal protein, fiber, and sodium.

^c^ HRs were adjusted for age, sex, BMI, race, education, marital status, household income, smoking, alcohol, vigorous physical activity, usual activity at work, hypertension, hypercholesteremia, history of heart disease, stroke, diabetes, and cancer at baseline, total energy, whole egg and HEI-2015.

# Table W. Sensitivity analyses for the associations of egg and cholesterol consumption with mortality by censoring participants at 8-y follow-up

|  | **Categories of egg or cholesterol consumption** | | | | |  |
| --- | --- | --- | --- | --- | --- | --- |
|  | **C1** | **C2** | **C3** | **C4** | **C5** | **P for trend** |
| **All-cause** |  |  |  |  |  |  |
| Whole egg^a^ | 1.00 | 1.05 (1.02-1.08) | 1.10 (1.06-1.13) | 1.14 (1.11-1.18) | 1.21 (1.17-1.24) | <0.001 |
| Cholesterol^b^ | 1.00 | 1.03 (1.00-1.06) | 1.09 (1.05-1.12) | 1.13 (1.09-1.17) | 1.22 (1.17-1.26) | <0.001 |
| Egg whites/substitutes^c^ | 1.00 | 0.94 (0.90-0.98) |  |  |  | 0.001 |
| **Cardiovascular disease** |  |  |  |  |  |  |
| Whole egg^a^ | 1.00 | 0.99 (0.94-1.05) | 1.05 (0.99-1.11) | 1.12 (1.06-1.19) | 1.18 (1.12-1.25) | <0.001 |
| Cholesterol^b^ | 1.00 | 1.05 (0.99-1.11) | 1.08 (1.02-1.14) | 1.16 (1.09-1.23) | 1.21 (1.12-1.29) | <0.001 |
| Egg whites/substitutes^c^ | 1.00 | 1.00 (0.93-1.06) |  |  |  | 0.89 |
| **Cancer** |  |  |  |  |  |  |
| Whole egg^a^ | 1.00 | 1.10 (1.04-1.16) | 1.14 (1.09-1.20) | 1.17 (1.11-1.23) | 1.20 (1.15-1.27) | <0.001 |
| Cholesterol^b^ | 1.00 | 1.04 (0.99-1.10) | 1.11 (1.05-1.17) | 1.10 (1.04-1.17) | 1.20 (1.13-1.28) | <0.001 |
| Egg whites/substitutes^c^ | 1.00 | 0.95 (0.89-1.01) |  |  |  | 0.10 |
| **Respiratory disease** |  |  |  |  |  |  |
| Whole egg^a^ | 1.00 | 1.24 (1.09-1.42) | 1.28 (1.12-1.46) | 1.34 (1.18-1.53) | 1.65 (1.46-1.87) | <0.001 |
| Cholesterol^b^ | 1.00 | 0.99 (0.87-1.12) | 1.05 (0.92-1.20) | 1.19 (1.03-1.36) | 1.31 (1.13-1.53) | <0.001 |
| Egg whites/substitutes^c^ | 1.00 | 0.71 (0.60-0.83) |  |  |  | <0.001 |
| **Alzheimer's disease** |  |  |  |  |  |  |
| Whole egg^a^ | 1.00 | 0.82 (0.51-1.31) | 0.73 (0.45-1.17) | 1.03 (0.66-1.62) | 0.68 (0.42-1.11) | 0.31 |
| Cholesterol^b^ | 1.00 | 0.95 (0.60-1.50) | 1.16 (0.71-1.88) | 0.70 (0.39-1.25) | 0.73 (0.38-1.40) | 0.21 |
| Egg whites/substitutes^c^ | 1.00 | 0.72 (0.42-1.25) |  |  |  | 0.25 |
| **Diabetes** |  |  |  |  |  |  |
| Whole egg^a^ | 1.00 | 1.17 (0.96-1.43) | 1.06 (0.87-1.30) | 1.10 (0.91-1.33) | 1.30 (1.10-1.56) | 0.003 |
| Cholesterol^b^ | 1.00 | 0.92 (0.74-1.14) | 1.14 (0.92-1.42) | 1.20 (0.96-1.50) | 1.42 (1.12-1.81) | <0.001 |
| Egg whites/substitutes^c^ | 1.00 | 1.13 (0.90-1.43) |  |  |  | 0.28 |
|  |  |  |  |  |  |  |
| **Infections** |  |  |  |  |  |  |
| Whole egg^a^ | 1.00 | 1.04 (0.82-1.32) | 1.06 (0.83-1.34) | 1.33 (1.06-1.68) | 1.13 (0.90-1.42) | 0.22 |
| Cholesterol^b^ | 1.00 | 0.87 (0.68-1.10) | 1.02 (0.79-1.30) | 0.98 (0.75-1.29) | 1.17 (0.87-1.56) | 0.10 |
| Egg whites/substitutes^c^ | 1.00 | 1.03 (0.77-1.38) |  |  |  | 0.83 |
| **Kidney disease** |  |  |  |  |  |  |
| Whole egg^a^ | 1.00 | 1.00 (0.72-1.38) | 1.08 (0.79-1.48) | 1.09 (0.80-1.48) | 1.26 (0.94-1.68) | 0.06 |
| Cholesterol^b^ | 1.00 | 0.84 (0.62-1.16) | 0.87 (0.62-1.22) | 0.99 (0.69-1.40) | 1.14 (0.78-1.67) | 0.17 |
| Egg whites/substitutes^c^ | 1.00 | 1.12 (0.77-1.63) |  |  |  | 0.54 |
| **Chronic liver disease** |  |  |  |  |  |  |
| Whole egg^a^ | 1.00 | 1.31 (0.92-1.86) | 1.18 (0.83-1.69) | 1.43 (1.01-2.02) | 1.27 (0.90-1.78) | 0.45 |
| Cholesterol^b^ | 1.00 | 1.16 (0.84-1.61) | 1.05 (0.73-1.50) | 1.51 (1.05-2.16) | 1.39 (0.93-2.09) | 0.07 |
| Egg whites/substitutes^c^ | 1.00 | 0.71 (0.45-1.12) |  |  |  | 0.14 |
| **Other causes** |  |  |  |  |  |  |
| Whole egg^a^ | 1.00 | 0.96 (0.89-1.04) | 1.04 (0.96-1.12) | 1.02 (0.95-1.10) | 1.11 (1.03-1.19) | <0.001 |
| Cholesterol^b^ | 1.00 | 1.00 (0.93-1.08) | 1.07 (0.99-1.16) | 1.11 (1.02-1.21) | 1.20 (1.09-1.33) | <0.001 |
| Egg whites/substitutes^c^ | 1.00 | 0.85 (0.78-0.94) |  |  |  | <0.001 |

Data are multivariable HRs (95% CIs) for whole egg and cholesterol intakes (quintiles) and egg whites/substitutes consumption (consumers vs. non-consumers).

^a^ HRs were adjusted for age, sex, BMI, race, education, marital status, household income, smoking, alcohol, vigorous physical activity, usual activity at work, hypertension, hypercholesteremia, history of heart disease, stroke, diabetes, and cancer at baseline, total energy, egg whites/substitutes and HEI-2015.

^b^ HRs were adjusted for age, sex, BMI, race, education, marital status, household income, smoking, alcohol, vigorous physical activity, usual activity at work, hypertension, hypercholesteremia, history of heart disease, stroke, diabetes, and cancer at baseline, total energy and intakes of saturated fat, polyunsaturated fat, monounsaturated fat, trans fat, animal protein, fiber, and sodium.

^c^ HRs were adjusted for age, sex, BMI, race, education, marital status, household income, smoking, alcohol, vigorous physical activity, usual activity at work, hypertension, hypercholesteremia, history of heart disease, stroke, diabetes, and cancer at baseline, total energy, whole egg and HEI-2015.
