# Supplementary material for: Egg and cholesterol consumption and mortality from cardiovascular and different causes in the United States: A population-based cohort study
Source: PLoS Med. 2021 Feb 9;18(2):e1003508. doi: 10.1371/journal.pmed.1003508 (PMC7872242; doi:10.1371/journal.pmed.1003508)
Supplement: S1 Text — (PDF) [file pmed.1003508.s003.pdf]

NIH-AARP STaRs Proposal Details

Proposal Number: 201903-0001

Investigator: Jingjing Jiao

Originally Submitted: 3/4/2019

Last Updated: 4/5/2019 11:52:14 AM

Contact Information

Email: jingjingjiao@zju.edu.cn

Phone:

Fax:

Institution: Other (Specify)

Address: 866 Yuhangtang Road, Hangzhou, China

Hangzhou, Zhejiang 310058

Project Information

Proposal Title: Dietary Cholesterol and Egg intakes in Relation to Total and Cause-specific Mortality in the NIH-AARP Cohort

Cancer Site /Outcome: MOR

Exposure: Intakes of cholesterol and eggs (g/day)

Geographic Identifiers: Don't know

Timeline

Data Tables: 4/15/2019

Abstract: 4/30/2019

Manuscript Draft: 5/31/2019

Anticipated Submission: 6/30/2019

Proposed Author List

Investigator Name	Investigator Type	Institution	Email
Jingjing Jiao	Investigator	Zhejiang University	jingjingjiao@zju.edu.cn
Yu Zhang	Investigator	Zhejiang University	y_zhang@zju.edu.cn
Xiaoqian Chen	Investigator	Zhejiang University	21713044@zju.edu.cn
Lei Mao	Investigator	Zhejiang University	maolei1108@foxmail.com
Pan Zhuang	Investigator	Zhejiang University	panzhuang@zju.edu.cn

Proposal / Abstract

Background (including justification)
In the past, limiting dietary cholesterol intake to 300 mg/day has been widely recommended for prevention of cardiovascular disease (CVD) (1). However, accumulating evidence showed that ingestion of dietary cholesterol had only modest effects on LDL and HDL cholesterol and little influence on the ratio of LDL to HDL (2). Eggs are a major source of dietary cholesterol, whereas other beneficial nutrients such as unsaturated fats, lecithins and B-vitamins are also present, potentially offsetting any possible increased risk due to the cholesterol content (3). Therefore, due to the lack of effect of dietary cholesterol on blood cholesterol, the 2015 Dietary Guidelines Advisory Committee Report did not carry forward the upper limit for dietary cholesterol and recommended eggs as part of a healthy diet (4). Nonetheless, the effects of dietary cholesterol and eggs on cardiovascular health still remain unclear. A meta-analysis concluded that dietary cholesterol was not associated with the risk of cardiovascular death, coronary artery disease or stroke (5).

However, these reviewed studies had relatively smaller sample sizes or fewer cases, were too low-powered to obtain precise effect estimates, and were unable to examine the associations with mortality from various causes, such as cancer and diabetes. Several lines of evidence suggested positive relationships of dietary cholesterol with breast and pancreatic cancer (6, 7). Previous studies regarding egg consumption have also been inconsistent. Although a previous meta-analysis reported a doseresponse positive association of egg consumption with CVD risk (8), the latest meta-analysis revealed that egg consumption was not associated with increase in CVD or all-cause mortality whereas related to a small reduction in stroke (9). Notably, egg consumption has been associated with an increased risk of diabetes among the general population and CVD comorbidity among diabetic populations (2, 10).
Overall, the scientific evidence for recommendations on dietary cholesterol and eggs is lacking. Therefore, a large-scale study is needed for assessing substantial associations of dietary cholesterol and egg intakes with various causes of death in American populations. Given the large number of participants and death cases in the AAPR cohort, findings from this study will provide the evidence base to inform dietary recommendations on dietary cholesterol and egg consumption for clinicians and policy makers.
Objectives
1. To investigate the associations of dietary cholesterol and egg intakes with total and cause-specific mortality in the NIH-AARP Cohort.
2. To evaluate whether the proposed associations differ among diabetic patients versus non-diabetic participants.
Study population
Participants from the NIH-AARP Cohort with complete dietary data at baseline.
Exclusions
Participants with implausible energy intake (4200 kcal/d for men and 3500 kcal/d for women).
Exposure
Intakes of cholesterol and eggs (g/day)
Outcome
All-cause mortality and cause-specific mortality from different causes
Statistical analysis (including statistical method, covariates, and sensitivity analysis)
Intakes of cholesterol and eggs will be expressed as functions of energy density (g · 2000 kcal-1 · d-1) by the nutrient density method. Cox proportional hazards regression models will be used to estimate hazard ratios (HRs) and 95% confidence intervals (CIs) for mortality according to cholesterol and egg intakes with the lowest quintile (cholesterol) or non-consumers (egg) as the reference group. Established or suspected risk factors for death will be considered as covariates, including age, sex, race, marital status, education, household income, body mass index (BMI), alcohol, smoking, vigorous physical activity, usual activity at work, perceived health condition, history of cancer, heart disease, stroke, and diabetes at baseline, history of hypertension, history of hypercholesterolemia, family history of cancer, cholesterol-lowering medications use, aspirin use, hormones use (for women only), intake of total energy, and consumption of red and processed meats, vegetables, fruits, whole grains, legumes, dairy products, potatoes, and sugar sweetened beverages.
We will also assess the effects of substituting eggs for 1 serving of other common food groups (such as red meats, dairy products and legumes) on risk of death by including continuous variables for both eggs and the other food in the model along with other non-dietary confounders. In a secondary analysis, we will further analyze the associations for whole egg, egg whites and egg substitutes, respectively. Subgroup analyses will also be conducted according to important potential effect modifiers, such as diabetes, history of hypertension and hypercholesterolemia. P values for interactions will be tested by likelihood-ratio test.
Several sensitivity analyses will be performed: (i) further adjusting for a healthy eating index-2015 to see whether findings materially change; (ii) excluding those with heart disease, stroke, cancer and diabetes at baseline; and (iii) excluding the initial 4 years of follow-up.
References
1. Krauss RM, Eckel RH, Howard B, Appel LJ, Daniels SR, Deckelbaum RJ, Erdman JW, Jr., Kris-Etherton P, Goldberg IJ, Kotchen TA, et al. AHA Dietary Guidelines: revision 2000: A statement for healthcare professionals from the Nutrition Committee of the American Heart Association. Circulation 2000;102(18):2284-99.
2. Shin JY, Xun P, Nakamura Y, He K. Egg consumption in relation to risk of cardiovascular disease and diabetes: a systematic review and meta-analysis. The American journal of clinical nutrition 2013;98(1):146-59. doi: 10.3945/ajcn.112.051318.
3. Hu FB, Stampfer MJ, Rimm EB, Manson JE, Ascherio A, Colditz GA, Rosner BA, Spiegelman D, Speizer FE, Sacks FM, et al. A Prospective Study of Egg Consumption and Risk of Cardiovascular Disease in Men and Women. JAMA 1999;281(15):1387-94. doi: 10.1001/jama.281.15.1387.
4. Millen BEea. Scientific Report of the 2015 Dietary Guidelines Advisory Committee. In: Services USDoHaH, ed.: Office of Disease Prevention and Health Promotion, 2015:3.
5. Raman G, Berger S, Jacques PF, Vishwanathan R, Johnson EJ. Dietary cholesterol and cardiovascular disease: a systematic review and meta-analysis. The American Journal of Clinical Nutrition 2015;102(2):276-94. doi: 10.3945/ajcn.114.100305.

6. Li C, Yang L, Zhang D, Jiang W. Systematic review and meta-analysis suggest that dietary cholesterol intake increases risk of breast cancer. Nutrition Research 2016;36(7):627-35. doi: https://doi.org/10.1016/j.nutres.2016.04.009.
7. Wang J, Wang W-J, Zhai L, Zhang D-F. Association of cholesterol with risk of pancreatic cancer: a meta-analysis. World journal of gastroenterology 2015;21(12):3711-9. doi: 10.3748/wjg.v21.i12.3711.
8. Li Y, Zhou C, Zhou X, Li L. Egg consumption and risk of cardiovascular diseases and diabetes: A meta-analysis. Atherosclerosis 2013;229(2):524-30. doi: 10.1016/j.atherosclerosis.2013.04.003.
9. Xu L, Lam TH, Jiang CQ, Zhang WS, Zhu F, Jin YL, Woo J, Cheng KK, Thomas GN. Egg consumption and the risk of cardiovascular disease and all-cause mortality: Guangzhou Biobank Cohort Study and meta-analyses. European Journal of Nutrition 2018. doi: 10.1007/s00394-018-1692-3.
10. Rong Y, Chen L, Zhu T, Song Y, Yu M, Shan Z, Sands A, Hu FB, Liu L. Egg consumption and risk of coronary heart disease and stroke: dose-response meta-analysis of prospective cohort studies. BMJ : British Medical Journal 2013;346:e8539. doi: 10.1136/bmj.e8539.

Additional Comments

Primary Investigator CV

Jingjing Jiao, PhD
Associate professor
Chronic Disease Research Institute
Department of Nutrition and Food Hygiene
School of Public Health
Zhejiang University
Contact Information
Room 901, Scientific Research Building
Zhejiang University School of Medicine
866 Yuhantang Road
Hangzhou, 310058, China
E-mail: jingjingjiao@zju.edu.cn
Research Interests
· Investigation of dietary factors, using nutritional or molecular epidemiologic approaches, in the cause and prevention of cardiovascular disease, cancer, and other chronic diseases in cohort studies
· Investigation of nutrients, such as polyunsaturated fatty acids, in the prevention and control of cardiovascular diseases and other chronic diseases
· Molecular nutrition and nutrigenomic studies on the occurrence, intervention and control of cardiovascular diseases and other chronic diseases
Education Background and Work Experience
· 2015- : Associate professor, Department of Nutrition and Food Hygiene, School of Public Health, Zhejiang University, Hangzhou, Zhejiang, China
· 2012-2014: Lecturer, Department of Nutrition and Food Hygiene, School of Public Health, Zhejiang University, Hangzhou, Zhejiang, China
· 2009-2011: Postdoctoral research fellow, Massachusetts General Hospital and Harvard Medical School, Boston, MA, USA
· 2003-2008: Ph.D. in Food Science, Department of Food Science and Nutrition, College of Biosystems Engineering and Food Science, Zhejiang University, Hangzhou, Zhejiang, China
· 1999-2003: B.S. in Food Science, College of Food Science and Engineering, Shanxi Agriculture University, Jinzhong, Shanxi, China
Academic Committee Membership
2013-Now: Member, Chinese Nutrition Society
2013-Now: Member, Zhejiang Provincial Nutrition Society
Peer-reviewed Publications
1. Zhuang P, Zhang Y, He W, Chen XQ, Chen JN, He LL, Mao L, Wu F, Jiao JJ*. Dietary fats in relation to total and cause-specific mortality in a prospective cohort of 521,120 individuals with 16 years of follow-up. Circulation Research 2019;124(5):757-68.
2. Zhuang P, Cheng LF, Wang J, Zhang Y*, Jiao JJ*. Saturated fatty acid intake is associated with total mortality in a nationwide cohort study. Journal of Nutrition 2019;149(1):68-77.
3. Huang MM, Zhu FH, Jiao JJ, Wang J, Zhang Y*. Exposure to acrylamide disrupts cardiomyocyte interactions during ventricular morphogenesis in zebrafish embryos. Science of the Total Environment 2019;656:1337-45.
4. Zhuang P, Wang WQ, Wang J, Zhang Y*, Jiao JJ*. Polyunsaturated fatty acids intake, omega-6/omega-3 ratio and mortality: findings from two independent nationwide cohorts. Clinical Nutrition 2018 Mar 3, doi:

10.1016/j.clnu.2018.02.019.
5. Huang MM, Zhuang P, Jiao JJ, Wang J, Chen XY, Zhang Y*. Potato consumption is prospectively associated with risk of hypertension: An 11.3-year longitudinal cohort study. Clinical Nutrition 2018 Jul 2, doi: 10.1016/j.clnu.2018.06.973.
6. Zhang Y, Zhuang P, He W, Chen JN, Wang WQ, Freedman ND, Abnet CC, Wang JB*, Jiao JJ*. Association of fish and long-chain omega-3 fatty acids intakes with total and cause-specific mortality: prospective analysis of 421 309 individuals. Journal of Internal Medicine 2018;284:399-417.
7. Zhuang P, Wang WQ, Wang J, Zhang Y*, Jiao JJ*. Current level of fish consumption is associated with mortality in Chinese but not US adults: new findings from two nationwide cohort studies with 14 and 9.8 years of follow-up. Molecular Nutrition & Food Research 2018;62(8):e1700898.
8. Zhuang P, Shou QY, Wang WQ, He LL, Wang J, Chen JN, Zhang Y*, Jiao JJ*. Essential fatty acids linoleic acid and ±-linolenic acid sex-dependently regulate glucose homeostasis in obesity. Molecular Nutrition & Food Research 2018;62(17):e1800448.
9. Huang MM, Jiao JJ, Zhuang P, Chen XY, Wang J, Zhang Y*. Serum polyfluoroalkyl chemicals are associated with risk of cardiovascular diseases in national US population. Environment International 2018;119:37-46.
10. Zhang Y, Huang MM, Zhuang P, Jiao JJ, Chen XY, Wang J, Wu YN*. Exposure to acrylamide and the risk of cardiovascular diseases in the National Health and Nutrition Examination Survey 2003-2006. Environment International 2018;117:154-63.
11. Huang MM, Jiao JJ, Wang J, Chen XY, Zhang Y*. Associations of hemoglobin biomarker levels of acrylamide and all-cause and cardiovascular disease mortality among US adults: National Health and Nutrition Examination Survey 2003-2006. Environmental Pollution 2018;238:852-8.
12. Huang MM, Zhuang P, Jiao JJ, Wang J, Zhang Y*. Association of acrylamide hemoglobin biomarkers with obesity, abdominal obesity and overweight in general US population: NHANES 2003-2006. Science of the Total Environment 2018;631-632:589-96.
13. Huang MM, Jiao JJ, Wang J, Xia ZD, Zhang Y*. Characterization of acrylamide-induced oxidative stress and cardiovascular toxicity in zebrafish embryos. Journal of Hazardous Materials 2018;347:451-60.
14. Huang MM, Jiao JJ, Wang J, Xia ZD, Zhang Y*. Exposure to acrylamide induces cardiac developmental toxicity in zebrafish during cardiogenesis. Environmental Pollution 2018;234:656-66.
15. Zhuang P, Shou QY, Lu YH, Wang GF, Qiu JN, Wang J, He LL, Chen JN, Jiao JJ*, Zhang Y*. Arachidonic acid sex-dependently affects obesity through linking gut microbiota-driven inflammation to hypothalamus-adipose-liver axis. Biochimica Biophysica et Acta Molecular Basis of Disease 2017;1863:2715-26.
16. Jiao JJ, Wei Y, Chen JN, Chen XY, Zhang Y*. Anti-aging and redox state regulation effects of A-type proanthocyanidins-rich cranberry concentrate and its comparison with grape seed extract in mice. Journal of Functional Foods 2017;30:63-73.
17. Chen JN, Wei Y, Chen XY, Jiao JJ*, Zhang Y*. Polyunsaturated fatty acids ameliorate aging via redox-telomere-antioncogene axis. Oncotarget 2017;8(5):7301-14.
18. Zhang Y, Chen JN, Qiu JN, Li YJ, Wang JB, Jiao JJ*. Intakes of fish and PUFAs and mild-to-severe cognitive impairment risks: a dose-response meta-analysis of 21 cohort studies. American Journal of Clinical Nutrition 2016;103(2):330-40.
19. He W, Li QQ, Yang M, Jiao JJ, Ma XG, Zhou YJ, Song AH, Heymsfield SB, Zhang SC, Zhu SK*. Lower BMI cutoffs to define overweight and obesity in China. Obesity 2015;23(3):684-91.
20. Yang M, Lin J, Ma XG, Zhu CN, Wei C, Wang L, Jiao JJ, Zhu SK*. Truncal and leg fat associations with metabolic risk factors among Chinese adults. Asia Pacific Journal of Clinical Nutrition 2016;25(4):798-809.
21. Jiao JJ, Li QQ, Chu JJ, Zeng WJ, Yang M, Zhu SK*. Effect of n-3 PUFA supplementation on cognitive function throughout the life span from infancy to old age: a systematic review and meta-analysis of randomized controlled trials. American Journal of Clinical Nutrition 2014;100(6):1422-36.
22. Fu XH, Song AH, Zhou YJ, Ma XG, Jiao JJ, Yang M, Zhu SK*. Association of regional body fat with metabolic risks in Chinese women. Public Health Nutrition 2014;17(10):2316-24.
Funded Projects
1. Project title: Mechanism of action for effect of n-3 polyunsaturated fatty acids on promoting white-to-beige process of adipose and regulating glucose homeostasis imbalance via adipose-gut microbiota-liver axis
Duration: 2018-2021
Agency and Grant#: National Natural Science Foundation of China (81773419)
Role: PI
2. Project title: Mechanistic studies on amelioration effect of salmon unsaturated fatty acids on insulin resistance based on synergistic regulation of GLUT4 vesicular trafficking and adipose browning
Duration: 2015-2017
Agency and Grant#: National Natural Science Foundation of China (31401659)
Role: PI
3. Project title: Associations of plasma phospholipid fatty acids and body fat composition and metabolism syndrome
Duration: 2015-2016

Agency and Grant#: National Fundamental Funds for the Central Universities of China (2015QNA7014)
Role: PI
4. Project title: Effect of marine n-3 polyunsaturated fatty acids on promotion of the browning of white adipose and regulation of nutrition-gene interaction
Duration: 2014-2016
Agency and Grant#: Zhejiang Natural Science Foundation (LY14C200008)
Role: PI
5. Project title: CMB Collaborating Program (CP) on modifiable chronic disease related risk factors control and health promotion
Duration: 2013-2015
Agency and Grant#: China Medical Board Foundation (12-108)
Role: Co-PI
