# Supplementary material for: Egg and cholesterol consumption and mortality from cardiovascular and different causes in the United States: A population-based cohort study
Source: PLoS Med. 2021 Feb 9;18(2):e1003508. doi: 10.1371/journal.pmed.1003508 (PMC7872242; doi:10.1371/journal.pmed.1003508)
Supplement: Attachment — Submitted filename: Response to reviewers(Plos Med).docx [file pmed.1003508.s004.docx]

**Response to Reviewers**

**Manuscript title:** Egg and cholesterol consumption and mortality from cardiovascular and different causes: population-based cohort study of 521,120 participants

**Authors:** Pan Zhuang, Fei Wu, Lei Mao, Fanghuan Zhu, Yiju Zhang, Xiaoqian Chen, Jingjing Jiao*, Yu Zhang*

We appreciate all the comments raised by the editor, the committee, and the reviewers and have provided point-by-point responses to all concerns.

**Response to Requests from the Editors:**

**Please revisit the data statement. It would appear that** **"No - there are some restrictions on access to data" would be appropriate, as you note restrictions in the subsequent section.**

**Answer**: Done. We have added "No - there are some restrictions on access to data" in the data statement.

**Please** **remove the word "prospective" from the title (we think that the current study is a retrospective analysis of prospectively gathered data).**

**Answer**: Done. We have removed the word "prospective" from the title.

**You may wish to note that the analysis involves a US population. Please revisit the word "nationwide" at line 28.**

**Answer**: We have changed the word "nationwide" into “US”.

**Page 2, line 28:**

This study aimed to examine the associations of eggs and cholesterol intakes with mortality from all-cause, cardiovascular disease (CVD) and other causes in a US population.

**Around line 90 it appears that the cohort comprised people from** **6 states and 2 cities, and we suggest briefly stating this information in the "methods and findings" section of your abstract instead.**

**Answer**: We appreciate this comment and have now included this information in the "methods and findings" section.

**Page 2, lines 30-31:**

Overall 521,120 participants aged 50-71 y were recruited from 6 states and 2 cities in the US between 1995 and 1996 and prospectively followed up until 2012.

**At line 28, we ask you to adopt the passive voice, i.e. "X participants were identified and prospectively followed ..." to avoid the implication that the present analysis is a prospective one.**

**Answer**: Done. We have revised this sentence to "Overall 521,120 participants aged 50-71 y were recruited from 6 states and 2 cities in the US between 1995 and 1996 and prospectively followed up until 2012."

**Page 2, lines 30-31:**

Overall 521,120 participants aged 50-71 y were recruited from 6 states and 2 cities in the US between 1995 and 1996 and prospectively followed up until 2012.

**At line 28 and elsewhere, please substitute "years". We ask you to adapt the wording in your abstract to** **quote the dates of start and end of follow-up.**

**Answer**: Done. We have quoted the dates of start and end of follow-up.

**Page 2, lines 30-31:**

521,120 participants aged 50-71 y were recruited from 6 states and 2 cities in the US between 1995 and 1996 and prospectively followed up until 2012.

**Please adapt the "Methods and findings" subsection of your abstract by adding a new final sentence, summarizing 2-3 of the study's main limitations.**

**Answer**: Done. We have added main limitations in the Methods and findings section.

**Page 3, lines 48-51:**

The study is observational and residual confounding is possible despite extensive adjustment for acknowledged dietary and lifestyle risk factors. Since dietary consumption was assessed from self-report, measurement error is inevitable.

**At line 48, please start the sentence with "In this study ..." or similar.**

**Answer**: Done. We have added “In this study” to this sentence.

**Page 3, lines 53-54:**

In this study, intakes of eggs and cholesterol were associated with higher all-cause, CVD and cancer mortality.

**After the abstract, we will need to ask you to add a new and accessible** **"author summary" section in non-identical prose. You may find it helpful to consult one or two recent research papers in PLOS Medicine to get a sense of the preferred style.**

**Answer**: Done. We have added an "Author summary" section after the abstract.

**Page 5, lines 64-94:**

**Author summary**

**Why was this study done?**

- The 2015-2020 Dietary Guidelines for Americans recommend eating as little dietary cholesterol as possible while consuming a healthy eating pattern, but also state cholesterol is not a nutrient of concern for overconsumption.
- There is limited and inconclusive evidence on the associations of egg or dietary cholesterol intake with all-cause and CVD mortality.
- Associations of egg or dietary cholesterol consumption with mortality from non-cardiovascular causes, including cancer, respiratory disease, diabetes and Alzheimer's disease are largely unknown.

**What did the researchers do and find?**

- We used data of 521,120 participants from the NIH-AARP Diet and Health Study with a median follow-up of 16 years to assess the associations of eggs and cholesterol intakes with all-cause and cause-specific mortality.
- Whole egg and cholesterol intakes are positively associated with risk of total and major cause-specific mortality whereas egg whites/substitutes consumption is inversely associated with mortality.
- Mediation models estimated that the increased mortality associated with whole egg intake was largely influenced by cholesterol intake.
- Replacing half a whole egg with egg whites/substitutes or other less cholesterol-containing protein sources is associated with lower overall mortality and mortality from major causes, including cardiovascular disease, cancer, and respiratory disease.

**What do these findings mean?**

- The current recommendations for egg and dietary cholesterol consumption from the US Dietary Guidelines might lead to increases in cholesterol intake, which could be detrimental to the prevention of premature death.
- Clinicians and policy makers should continue to highlight limiting cholesterol intake in the US dietary recommendations considering our results.
- The US dietary guidelines may recommend replacing whole eggs with egg whites/substitutes or alternative protein sources for facilitating cardiovascular health and long-term survival.

**Early in the methods section of your main text, please state whether** **the current analysis had a protocol or prespecified analysis plan, and if so** **attach the relevant document(s) as a supplementary file, referred to** **in the text. Please highlight analyses that were not prespecified.**

**Answer**: The current analysis did have a protocol/prespecified analysis plan. We have attached the relevant document(s) as a supplementary file and referred to in the text.

**Page 10, lines 179-180:**

The analysis plan was developed prospectively (**S1 Text**) with later adjustments such as linear models and mediation analyses.

**Please refer to the** **attached STROBE checklist early in your methods section ("See S1_STROBE_Checklist" or similar).**

**Answer**: Done. We have referred to the attached STROBE checklist early in our Methods section.

**Page 8, lines 138-140:**

This study is reported as per the Strengthening the Reporting of Observational Studies in Epidemiology (STROBE) Statement (see **S1 STROBE Checklist**).

**At line 389, please remove the word "nationwide".**

**Answer**: Done. We have removed the word "nationwide".

**Please** **remove the information on funding, competing interests and data availability from the end of the main text. In the event of publication, this information will appear in the article metadata via entries in the submission form.**

**Answer**: Done. We have removed the information on funding, competing interests and data availability from the end of the main text.

**In your reference list, please ensure that 6 author names where appropriate are listed rather than 3, followed by "et al.".**

**Answer**: Done. We have revised all the references accordingly.

**In table 1, we suggest** **substituting "ethnicity" for "race".**

**Answer**: Done. We have substituted "ethnicity" for "race".

**Response to Comments from Reviewer #1:**

**The paper by Zhuang and colleagues address an interesting research question, that is the relationship between egg intake and mortality, on which, to date, there is large inconsistency among studies. Moreover, the major novelty of the study is the mediation analysis which attempts to understand the nutrition-related mechanisms through which egg may exert their effect on health. Overall the paper is of interest, however there are some suggestions that may improve the quality of the work.**

**Answer**: We appreciate the positive comment from this reviewer.

1. **Eggs are natural sources not only of cholesterol, but also of saturated fat and protein. I would suggest the authors to test also these nutritional factors as potentially lining egg intake to mortality risk.**

**Answer**: We appreciate this insightful comment. We agree that saturated fat and animal protein could also be mediators for the positive association of eggs with mortality. Therefore, we have also conducted the mediation analysis to estimate the mediation proportion with 95% CI of saturated fat and animal protein intakes for whole eggs (Table H in S1 Data). Our results showed that 16.3%-23.5% of the association between whole eggs and mortality was statistically accounted for by saturated fat, while animal protein only accounted for 1.2-4.6% of mortality associated with whole eggs. The mediation proportions of saturated fat and animal protein were much lower than cholesterol (49.6%-63.2%), indicating cholesterol is the main driver of the harmful association for whole eggs.

# Table H. Results of mediation analysis between whole egg and cholesterol for all-cause, CVD, and cancer mortality

| **Mediator** | **Proportion (%) of effect due to mediation (95% CI)** | **P value** |
| --- | --- | --- |
| **Cholesterol** |  |  |
| All-cause mortality | 63.2 (49.6 to 75.0) | <0.001 |
| CVD mortality | 62.3 (39.5 to 80.7) | <0.001 |
| Cancer mortality | 49.6 (31.9 to 67.4) | <0.001 |
| **Saturated fat** |  |  |
| Total mortality | 23.5 (19.2-28.4) | <0.001 |
| Cardiovascular mortality | 19.7 (13.2-28.3) | <0.001 |
| Cancer mortality | 16.3 (10.9-23.5) | <0.001 |
| **Animal protein** |  |  |
| Total mortality | 4.6 (3.2-6.5) | <0.001 |
| Cardiovascular mortality | 7.5 (5.0-11.1) | <0.001 |
| Cancer mortality | 1.2 (0.2-8.7) | 0.163 |

CI, confidence interval; CVD, cardiovascular disease.

2. **The authors used total dietary cholesterol as a mediator of the egg-mortality relation. Is it correct? Shouldn't they see only the** **cholesterol contained in eggs rather than the** **whole cholesterol content of diet? How can this be disentangled?**

**Answer**: We thank this thoughtful comment. Our mediation analysis was conducted to assess the mediating effect of egg-associated nutrients on the associations of egg consumption with major causes of death. We used total dietary cholesterol in the mediation model and the results could be interpreted as meaning that egg consumption increased one’s whole cholesterol content of diet and such increase further led to a higher risk of mortality. We agree that evaluating the cholesterol contained in eggs may be more precise, however, it is difficult to estimate it in the mediation model because the cholesterol intake from eggs is perfectly linearly correlated with egg consumption. In other words, we could not disentangle the associations for egg-sourced cholesterol from eggs because egg-sourced cholesterol is a perfect marker of egg consumption.

3. **The authors used the Mediate procedure in SAS. Why not relying on a** **counterfactual approach? Please, explain.**

**Answer**: We thank this thoughtful comment. Compared with the standard statistical approach, the counterfactual approach allowed for definitions of direct and indirect effects and for decomposition of a total effect into direct and indirect effects, even in models with interactions and nonlinearities [1]. This is relevant especially when mediation analysis is implemented in social science contexts where, for example, the exposure of interest might interact in its effect on the outcome with the mediator [1]. However, in our study we do not think eggs could interact with cholesterol on mortality risk. Therefore, we used the Mediate procedure in SAS to construct confidence intervals for the proportion by applying the partial likelihood function to two Cox models postulated on the same failure time variable [2], which has been widely used in cohort studies evaluating the mediation effect for mediators on exposures [3-6].

4. **In the mediation analysis, it is not clear to me if** **the proportion of effect explained by intermediate variables is obtained from a COX model including eggs as a categorical or continuous variable. This should be clarified.**

**Answer**: We apologize for missing this. The proportion of effect explained by intermediate variables is obtained from a COX model including eggs as a categorical variable. We have clarified this in the Methods section.

**Page 12, line 215:**

We further conducted the mediation analysis [26] to estimate the mediation proportion with 95% CI of cholesterol, saturated fat and animal protein intakes for whole egg consumption (quintiles) by using the publicly available “mediate” SAS macros…

5. **Also, were the HRs of the main analysis (Table 2) obtained from a COX PH or were those resulting from the mediate macro in SAS?**

**Answer**: The HRs of the main analysis (Table 2) were obtained from a COX PH.

6. **How many individuals are in the** **egg white /substitute consumers-non-consumers? It would be interesting to know. Moreover, egg white consumption may be a marker of socioeconomic status or healthy lifestyle, thus results may be affected by this bias. Please, provide more info on such type of consumers.**

**Answer**: There are 73,550 egg white/substitute consumers and 447,570 non-consumers. We have shown baseline characteristics of egg white/substitute consumers and non-consumers in Table C in S1 Data. Compared with non-consumers, egg white/substitute consumers were older, higher educated, more physically active, more often non-smokers, and had higher income and a healthier diet. Therefore, we carefully adjusted for income and these lifestyle factors in the Cox models. Results from Table E in S1 Data showed that the inverse associations for egg white/substitute with total and cancer mortality were attenuated but remained significant after adjusting for these confounders.

7. **The authors should acknowledge the study design (postal survey) as a major limitation of their work also highlighting main bias deriving from data collected through postal surveys.**

**Answer**: We appreciate this comment. Indeed, measurement errors of egg and cholesterol intakes assessed through postal surveys were inevitable despite using a validated FFQ. However, such errors in a prospective analysis would most often attenuate findings toward the null. We have added this limitation in the Discussion section.

**Page 21, lines 432-433:**

Firstly, measurement errors of egg and cholesterol intakes assessed through postal surveys were inevitable despite using a validated FFQ.

8. **How do authors believe these data can be read and interpreted in light of national dietary guidelines that seem to recommend much higher intake of eggs per week? A discussion on this would be helpful from a public health perspective.**

**Answer**: We thank this comment. The current US Dietary Guidelines recommend eggs as part of a healthy diet [7] and do not carry forward the upper limit intake. This might lead to increases in cholesterol intake, which could be detrimental to prevention of premature death. Our data should be considered for clinicians and policy makers in the development of dietary guidelines and updates. We have added these in the Discussion section.

**Pages 21-22, lines 453-457:**

The 2015-2020 US Dietary Guidelines recommend eggs as part of a healthy diet without an upper limit intake for cholesterol, which might lead to increases in cholesterol intake and thus could be detrimental to the prevention of premature death. Our results should be considered by clinicians and policy makers in updating dietary guidelines for Americans.

9. **Did the study collect blood samples? This would be interesting in light of analyzing which biological mechanisms underlie the association between egg and mortality.**

**Answer**: We appreciate this thoughtful comment. However, blood samples were not collected in the AARP study. It was hypothesized that egg/cholesterol consumption could increase plasma LDL-c and then led to higher risk of CVD. The latest meta-analysis in 2020 summarized 17 RCTs and showed that egg consumption increased LDL-c and LDL-c/HDL-c ratio among healthy populations [8]. Evidence from Mendelian randomization studies and randomized trials of LDL-lowering therapies consistently demonstrate that any mechanism of lowering LDL-c levels decreases the risk of atherosclerotic CVD, which indicated a causal role of LDL-c in atherosclerotic CVD development [9]. Therefore, there is accumulating evidence supporting the hypercholesterolemic effect (high LDL-c) as the biological mechanism for egg/dietary cholesterol on CVD. We have discussed these in the Discussion section.

**Page 18, lines 371-378:**

The latest meta-analysis summarizing results from 17 randomized controlled trials (RCTs) showed that egg consumption increased LDL-c and LDL-c/HDL-c ratio among healthy populations [33]. In addition, evidence from Mendelian randomization studies and RCTs of LDL-lowering therapies consistently demonstrate that any mechanism of lowering LDL-c levels decreases the risk of atherosclerotic CVD, suggesting a causal role of LDL-c in atherosclerotic CVD development [34]. Together, there is accumulating evidence supporting the hypercholesterolemic effect (high LDL-c) as the biological mechanism for egg/dietary cholesterol on CVD.

**Response to Comments from Reviewer #2:**

**This is a very interesting and important study around the hot topic regarding the safety of egg and dietary cholesterol consumption. A large national sample was used and a comprehensive set of analyses was conducted. Compared with previous studies, the current one examined cooking methods, egg whites, and cause-specific mortality from non-CVD non-cancer causes. These are novel aspects. Findings are valuable to contribute to the discussion of health concerns of egg and dietary cholesterol intake, which has been debated intensely lately, particularly after the publication of the JAMA egg paper last year. However, there are a number of issues that need to be addressed to improve the clarity of the paper and to present clearer results. My comments are listed below for consideration.**

**Answer**: We appreciate the positive comment from this reviewer.

1. **Abstract: specify units are per 2000 kcal for estimates. In line 31, I assume there is only one FFQ used, so questionnaires should be questionnaire?**

**Answer**: We appreciate this comment. We have specified the units (per 2000 kcal) for estimates. There is only one FFQ used at baseline. We have also replaced “questionnaires” by “questionnaire”.

**Page 2, lines 31-34:**

Intakes of whole eggs, egg whites/substitutes and cholesterol were assessed by a validated food frequency questionnaire. Cause-specific hazard models considering competing risks were used with the lowest quintiles of energy-adjusted intakes (2,000 kcal per day) as references.

2. **Lines 70-72, the interpretation of these guidelines is incorrect. It is not owing to the lack of evidence.** **There is evidence, but inconclusive or inconsistent. Please review the language in the 2013 AHA/ACC Guideline regarding dietary cholesterol. The background for the 2015-2020 Dietary Guidelines to remove the consumption limit for dietary cholesterol is because the mean consumption among US adults is already below 300 mg/day, so the focus can be placed on overall eating pattern instead of limiting cholesterol intake, although this recommendation is still debatable. Suggest revise the language.**

**Answer**: We appreciate this comment. We agree that there is evidence, but inconclusive or inconsistent for the association of cholesterol with CVD. In this sentence, we mean that there is a lack of evidence to support that notion that dietary cholesterol increases the risk of CVD. We apologize for this confusing statement and have revised the language accordingly.

**Page 7, lines 106-108**:

Owing to the inadequate evidence, the American Heart Association and American College of Cardiology [7,8] and the 2015-2020 US Dietary Guidelines [2,9] have not carried forward the upper limit for dietary cholesterol.

3. **Lines 75-78: Unclear and confusing sentence, please revise.**

**Answer**: We apologize for this confusing description and have revised the sentences accordingly.

**Page 7, lines 111-115:**

With regard to eggs, previous observational studies reported inconsistent results and most meta-analyses concluded non-significant associations with CVD or all-cause mortality among the general population [10-14]. However, these reviewed studies had relatively smaller sample sizes or fewer cases, which may not support robust effect estimates and examine the associations with mortality from various causes.

4. **Lines 84-85: Is quality of eggs a main exposure or amount of egg intake? What does quality of cholesterol intake mean? Did you study quality of cholesterol? This hypothesis does not fit in there at all. Suggest you** **delete it and replace it with the overall objective of your study.**

**Answer**: We apologize for this confusing sentence. We have deleted it and revised this sentence accordingly.

**Page 8, lines 120-122:**

Based on current large number of participants (*n*=521,120) and death cases (*n*=129,328), we hypothesized that egg and cholesterol intakes have significant associations with all-cause, CVD, and other cause-specific mortality.

5. **Line 110-112: The calibration methodology is unclear. Did the calibration help? What would be the associations if uncalibrated data were used?**

**Answer**: FFQs require algorithms that convert reported frequency of consumption and portion size for each food item into intakes and nutrient values [10]. The 1994-1996 USDA Continuing Survey of Food Intakes by Individuals [10] is a database established from a nationally representative sample of noninstitutionalized individuals who resided in all 50 states. We used it to calibrate DHQ data to derive intakes (g/day) for egg/cholesterol intakes and all other foods and nutrients. If data were uncalibrated, we could only analyze the association between egg consumption (in frequency) and mortality (Table N in S1 Data), which was similar to the main results. We have revised this sentence to be clearer.

**Page 9, lines 149-152:**

The 1994-1996 USDA Continuing Survey of Food Intakes by Individuals [17] was used to convert frequency of consumption and portion size for each food item into intakes, including intakes of eggs, other foods and dietary cholesterol.

6. **Based on restricted cubic spline models in Fig B, the associations appeared perfectly linear. I suggest you could** **use continuous exposure variables and avoid using** **quintiles. The quintile approach has many statistical shortcomings and it uses a lot of space while presenting not much additional information. By using** **linear models and 25 grams of eggs and 300 mg of cholesterol per day, you will have space to include** **all primary exposures and mortality outcomes in the main manuscript. This is a major novelty of this study and such important results should not be hidden in the supplement.**

**Answer**: As noted by the reviewer, the associations appeared linear for egg/cholesterol intakes. We agree that using continuous exposure variables might be more appropriate. We have used linear models and half a whole egg (25 g) and 300 mg of cholesterol per day and presented the results in the revised manuscript (see **Figure 1**), which included all primary exposures and mortality outcomes. However, our study used a FFQ to assess the consumption of egg/cholesterol. FFQs are not optimal for measuring an individual’s absolute intake, but rather useful in categorizing individuals on the basis of relative intakes (i.e., into groups with high, intermediate, or low consumption of specific foods or nutrients) and therefore our findings from analyzing quintiles also need to be presented as comparisons of higher versus lower egg/cholesterol consumption [11].

7. **Many models are presented, but the primary model is not specified. To this reviewer, for eggs and egg whites, the primary model can be model 2 + total energy + all food groups. For dietary cholesterol, the primary model can be model 2 + total energy + HEI-2015. Other models are interesting, but these covariates are not confounders. For example, for dietary cholesterol model 3 which adjusted for all major cholesterol containing foods: dietary cholesterol comes from these foods. Adjusting for these foods left little variation for cholesterol. The cholesterol in the model represents cholesterol from unadjusted food sources such as egg-containing baked goods (e.g. cake). The authors need to carefully think about the meaning and interpretation of each model.**

**Answer**: We appreciate the reviewer’s comment. The final models (model 6) were used as primary models. For eggs/egg whites, we used model 2+total energy+HEI-2015 as the primary model (model 6) because HEI-2015 covers almost all important food groups and it was used to adjust for the potentially confounding effect of the overall healthiness of the diet. For dietary cholesterol, the primary model was model 2 + total energy + saturated fat, polyunsaturated fat, monounsaturated fat, trans fat, animal protein, fiber, and sodium (model 6) because cholesterol is a nutrient and often coexists with saturated fat and animal protein. Hence, it is important to adjust for these nutrients when analyzing the associations for cholesterol. In model 3 for cholesterol, we adjusted for cholesterol-containing foods (eggs, red meat, fish, poultry, and dairy products) to test whether the positive associations for cholesterol could be explained by its parent foods. If the positive associations did not persist, then we would infer that the harmful associations could be due to other adverse components of these foods such as saturated fat and animal protein. However, we observed that the positive associations of cholesterol remained strong after adjusting for cholesterol-containing foods, which further validated the statement that cholesterol was a harmful component in cholesterol-containing foods.

8. **It is unclear if Spearman correlation analysis adjusted for energy.**

**Answer**: The Spearman correlation analysis was adjusted for energy. We have added this information in the footnote of Table D.

Footnote of Table D: ^a^ Spearman correlations were calculated using energy-adjusted intakes.

9. **Multiple comparisons are not adjusted. Consider to** **use <0.01 or <0.005 as the threshold to determine significance.**

**Answer**: We appreciate the reviewer’s comment. All statistical tests were based on a priori hypotheses and therefore there was no adjustment for multiple testing. Of note, the P values for the significant associations in our main results were all <0.001, therefore it makes little difference to use <0.01 or <0.005 as the threshold to determine the significance in our study.

10. **Substitution analysis: Suggest** **separating unprocessed red meat and processed red meat. Combine processed red meat with processed poultry to** **create a processed meat group.** **Separate dairy products into low-fat and high-fat dairies if possible. Separate nuts and legumes. These food groups are very different, so combining them into border groups is not a good idea.**

**Answer**: We thank this thoughtful comment. We have separated unprocessed red meat and processed red meat and create a processed meat group. Separating dairy products into low-fat and high-fat dairies has not been done due to the lack of relevant data. We have also separated nuts and legumes (see the new Figure 2).

**Figure 2. Multivariable-adjusted hazard ratios of all-cause and cause-specific mortality by replacing 1 whole egg with equivalent amounts of other protein sources.**

Forest plots show the multivariable HRs of (**A**) total, (**B**) CVD, (**C**) cancer, (**D**) RD, (**E**) AD, and (**F**) diabetes mortality associated with replacing half a whole egg/day with equivalent amount of egg whites/substitutes, poultry, fish, dairy products, or nuts/legumes. HRs were adjusted for age, sex, BMI, race, education, marital status, household income, smoking, alcohol, vigorous physical activity, usual activity at work, hypertension, hypercholesteremia, history of heart disease, stroke, diabetes, and cancer at baseline, total energy intake, and intakes of fruit, vegetables, potatoes, whole grains, refined grains, coffee, and sugar sweetened beverages. Horizontal lines represent 95% CIs. CI, confidence interval;CVD, cardiovascular disease; HR, hazard ratio.

11. **The substitution unit is** **50 grams per day. However, only about 2% of participants** **consumed this amount of eggs or more. For some foods such as nuts and legumes, their consumption amount may be similar to or lower than egg intake. Few people could meet the eligibility for substitution analysis. Suggest changing it to** **25 grams/per day or around median egg intake in the sample. In addition, milk is possibly the largest food group among all dairy products. Do you consider** **people who do not eat eggs (solid food) likely choose to drink milk (fluid) instead?**

**Answer**: We appreciate the reviewer’s comment and agree that only a small number of participants consumed ≥50 grams of eggs per day. We have changed 50 grams/per day into 25 grams/per day (half a whole egg) in the substitution analysis (see the new Figure 2 and the footnote). Although milk may be the largest food group among dairy products, other dairy products consumption like cheese consumption should also be considered. Importantly, milk is also a good food source for protein. Our primary aim for substitution analysis was to hypothetically comparing the health effects between eggs and other protein sources including both solid and fluid sources although people who do not eat eggs might not be likely to choose milk instead.

12. **Cooking method is understudied in the literature. It's interesting that results are largely similar between using fried and non-fried eggs.** **A few associations appeared stronger when using non-fried eggs. You may want to comment this finding in the discussion.**

**Answer**: We appreciate this thoughtful comment. As noted by the reviewer, a few associations such as CVD and diabetes mortality appeared stronger for non-fried eggs compared with fried eggs. We support the hypothesis that this may be due to the type of fat used for frying [12]. For example, plant-sourced oil used for cooking has been linked with lower risk of mortality in a nationwide cohort study [13]. However, we lacked data on type of fat used for frying eggs in this study. We have added these in the Discussion section.

**Page 20, lines 422-426:**

We noted that a few associations such as CVD and diabetes mortality appeared stronger for non-fried eggs compared with fried eggs, which may be due to the type of fat used for frying [24]. For example, plant-sourced cooking oil consumption has been linked with lower risk of mortality in a nationwide cohort study [56].

13. **Please remember to correct relevant places with copy-and-paste (e.g. Table B column head and Table E footnote Model 3). Please double check all tables and figures again.**

**Answer**: We apologize for these mistakes. We have changed “whole egg” into “cholesterol” in the column of Table B and changed “egg whites/substitutes” into “whole eggs” in the footnote regarding model 3 of Table E. Other tables and figures have also been carefully checked.

**Response to Comments from Reviewer #3:**

**This is a large observational study of the association between egg/cholesterol consumption and cause-specific mortality in the US. The major strengths of the analysis are its large sample size, extensive dietary data and prolonged duration of follow-up, resulting in a large number of deaths for analysis. The study finds positive (and broadly similar) associations between both egg and cholesterol consumption and risk of all-cause, vascular and cancer mortality. The authors estimate that a substantial proportion of the associations with egg consumption are mediated by cholesterol and give hypothetical estimates of the effects of substituting eggs/cholesterol intake with other protein sources. Consumption of egg whites/substitutes was associated with lower mortality from several causes when compared with non use. This is a nice study. I have just a few comments the authors may wish to respond to in a revision.**

**Answer**: We appreciate the positive comment from this reviewer.

1. **Lack of specificity of the associations. One would probably have expected** **the associations with egg and cholesterol consumption to be stronger for vascular mortality than for cancer mortality, whereas for cholesterol the opposite is seen (i.e., the association is strongest for cancer). How would the authors respond to the suggestion that this lack of specificity points towards residual confounding as a plausible explanation for at least some of the associations seen? Are there any causes of death the authors could use as '****negative controls' (i.e., causes that are reliably NOT associated with egg/cholesterol consumption?) For example, external causes of death? Is this included in the 'other' category in Figure 1? If such a reliable negative control could be demonstrated then this would give additional reassurance to the** **validity of the main analyses.**

**Answer**: We appreciate this thoughtful comment. As noted by the reviewer, cholesterol consumption had stronger positive association with cancer mortality than CVD mortality. Some evidence could be used to support the harmful effect of dietary cholesterol on cancers. Meta-analyses showed that dietary cholesterol increased the incidence of breast and pancreatic cancers [14, 15]. For putative mechanisms, elevated total and LDL cholesterol levels have been linked with increased levels of pro-inflammatory cytokines, such as interleukin-6 and tumor necrosis factor-α [16]. In animal models, cholesterol may increase the generation of secondary bile acids and enhance cancer incidence [17, 18]. In contrast, meta-analysis did not support a conclusive association between cholesterol intake and CVD outcomes [19]. Therefore, we think it is possible to see that the association with cholesterol consumption is stronger for cancer mortality than for vascular mortality. Nonetheless, we could not rule out the possibility of residual confounding despite the full adjustment of various risk factors.

External causes of death were included in the ‘other’ category. However, the cases of external causes of death were small (*n*<300). In addition, participants with higher egg/cholesterol consumption could have higher risk of incident cardiometabolic diseases/cancers and these patients might be less likely to survive from external causes. Therefore, external causes of death may not be used as reliable ‘negative controls’ in our study. Instead, we think death from infections could be used as a negative control because egg/cholesterol intake is not likely associated with death from infections and the cases are sufficient (*n*=2,569). As shown in Figure 1, egg/cholesterol intake was not associated with infections mortality, which could partly support the validity of our main analyses.

2. **It seems that** **high blood cholesterol was considered a confounder, but surely it is also (at least in part) a mediator of any association? Were the results materially different when analyses did NOT adjust for high cholesterol as a confounder?**

**Answer**: We appreciate this comment. We agree that high blood cholesterol could be a mediator of the positive associations of egg/cholesterol with mortality. The positive associations were slightly weakened after adjusting for high blood cholesterol, which indicated high blood cholesterol might at least in part contribute to the adverse associations for egg/cholesterol. We have mentioned this in the Discussion section.

**Page 18, 368-371:**

We observed that the positive associations were slightly weakened after adjusting for high blood cholesterol, which indicated high blood cholesterol might at least in part contribute to the adverse associations for egg/cholesterol.

3. **Competing risks (i.e., Fine-Gray) analyses were used as the primary method of analysis. Such methods estimate the sub-distribution hazard ratio and provide estimates of how cumulative incidence varies between the groups. They do not, however,** **estimate the cause-specific aetiological association (which is given by the simple Cox model that censors patients who die from an alternative cause at their date of death, i.e., it assumes the hypothetical absence of death from other causes). Since the aetiolgical association is what is of most interest in this paper I would expect the primary analysis to be from a standard Cox model rather than a competing risk model.**

**Answer**: There are 3 different regression approaches to modeling competing risks, including cause-specific proportional hazards model, subdistribution proportional hazards model, and parametric mixture model [20]. We used cause-specific hazard function rather than subdistribution hazard function (Fine-Gray) in our competing risks analyses. It measures the association of an exposure on the corresponding event in which the competing event contributes only by passively removing individuals from the risk set. Compared with Fine-Gray model, the cause-specific hazard model might be more applicable for studying the etiology of diseases [20]. We have stated that we used cause-specific hazard models in the Statistical Analysis section (Page 10, Line 184).

4. **I was a bit surprised so see no discussion of how reported egg/cholesterol consumption related to blood cholesterol, or to how blood cholesterol relates to vascular mortality risk (e.g., by reference to the statin trials and/or Mendelian randomisation studies). Even if this is not possible from this study, could the authors summarise evidence on egg consumption and blood cholesterol?**

**Answer**: We appreciate this comment. The association of egg consumption and serum cholesterol concentrations has been discussed for a long time. The latest meta-analysis published in 2020 summarized 17 RCTs and showed that egg consumption increased LDL-c and LDL-c/HDL-c ratio among healthy populations [8]. Evidence from mendelian randomization studies and randomized trials of LDL-lowering therapies consistently demonstrate that any mechanism of lowering LDL-c levels decreases the risk of atherosclerotic CVD, which indicated a causal role of LDL-c in atherosclerotic CVD development [9]. Therefore, there is accumulating evidence supporting the hypercholesterolemic effect (high LDL-c) as the biological mechanism for egg/dietary cholesterol on CVD. We have discussed these in the Discussion section.

**Page 18, lines 371-378:**

The latest meta-analysis summarizing results from 17 randomized controlled trials (RCTs) showed that egg consumption increased LDL-c and LDL-c/HDL-c ratio among healthy populations [33]. In addition, evidence from mendelian randomization studies and RCTs of LDL-lowering therapies consistently demonstrate that any mechanism of lowering LDL-c levels decreases the risk of atherosclerotic CVD, suggesting a causal role of LDL-c in atherosclerotic CVD development [34]. Together, accumulating evidence supported the hypercholesterolemic effect (high LDL-c) as the biological mechanism for egg/dietary cholesterol on CVD.

**MINOR COMMENTS**

1. **Line 84. I imagine the authors mean 'quantity' of eggs rather than 'quality'?**

**Answer**: We apologized for this and have revised this sentence accordingly.

**Page 8, lines 120-122:**

Based on current large number of participants (*n*=521,120) and death cases (*n*=129,328), we hypothesized that egg and cholesterol intakes have significant associations with all-cause, CVD, and other cause-specific mortality.

2. **The end of the abstract suggests this study arises from within a trial but the paper describes a cohort study.**

**Answer**: In addition to clinical trials, observational studies are also encouraged to be registered at https://clinicaltrials.gov/. We showed this information at the end of the abstract like other observational studies [21, 22] according to the reporting style of *PLoS Medicine*.

3. **Line 182. I know what a covariance is but what is an invariance?**

**Answer**: We apologize for this typo. We have changed “invariances” into “in variances”.

**Page 12, lines 222-224:**

The difference between regression coefficients and in variances and covariances was used to derive the HRs and 95% CIs of the substitution analyses [27].

4. **Table 2. The person-years is repeated in two separate rows.**

**Answer**: We apologized for this and have adjusted the person-years into one row.

5. **The appendix tables could be** **tidied up a bit with more thought given to how they are spaced.**

**Answer**: We thank this comment. We have tidied up appendix tables in S1 Data accordingly.

**Response to Comments from Reviewer #4:**

**Zhuang et al have conducted a longitudinal study on egg and cholesterol consumption and total mortality and mortality from different causes within the frame of the NIH-AARP Diet and Health Study. The paper is well written, the analyses are thorough and the results are robust. I have some comments which the authors may want to take into consideration.**

**Answer**: We appreciate the positive comment from this reviewer.

**1. Did the authors take egg consumption into account when they estimate the HEI-2015? It would be interesting to see the results without doing so.**

**Answer**: We thank this thoughtful comment. The HEI-2015 is composed of 13 components, including total fruit, whole fruit, total vegetables, greens and beans, whole grains, dairy, total protein foods, seafood and plant proteins, fatty acids, refined grains, sodium, added sugars, and saturated fats [23]. It does not include egg as an individual component. Although total protein foods include eggs, this food group is generally meat/fish/poultry-based for Americans [23]. Therefore, excluding eggs from this component makes little difference to the HEI-2015. We have tried excluding eggs when calculating HEI-2015 and adjusted for it and our results remained almost the same. Importantly, the HEI-2015 reveals a pattern of diet quality and represents overall diet quality. We included the HEI-2015 as an independent variable in the model to adjust for the potentially confounding effect of the overall healthiness of the diet. Therefore, we think it is not necessary to exclude eggs from HEI-2015. Other studies also did not exclude the exposures when adjusting for an overall diet quality score [3, 13, 24, 25].

**2. Lines 167-168: They authors may want to impute missing data.**

**Answer**: We thank this comment. Imputing missing data is often used when a high proportion of data are missing (>20%) to maintain statistical power. However, our study sample is large and the proportion of missing data was small (<5%). Therefore, we created indicator variables for missing data (<5%) in each covariate if necessary, like many other large-scale studies [26-28].

**3. It would be interesting if the authors would present main results interim of servings instead of grams.**

**Answer**: We have also evaluated the associations of egg consumption in frequency (<1 egg/month, 1-3 eggs/month, 1-2 eggs/week, 3-4 eggs/week, 5-6 eggs/week, and ≥1 egg/day) with mortality. Please see results in Table N in S1 Data.

**4. As for the replacing methods, did the authors mean to replace one egg for one serving of** **the other foods/food groups?**

**Answer**: In substitution analysis, we evaluated the risk of mortality by hypothetically replacing half an egg/day with equivalent amounts (25 g/day) of the other foods/food groups.

**5. Also for the substitution analyses, it would be interesting to see poultry as replacement but without processed poultry products and also to see the substitution models using read meat and red meat products.**

**Answer**: We appreciate this thoughtful comment. We have separated processed red meat and unprocessed red meat in the substitution analysis (Figure 2). Given that unprocessed fish may also be of interest, we have separated processed white meat and unprocessed white meat (unprocessed poultry + unprocessed fish) in the substitution analysis (Figure 2).

**Figure 2. Multivariable-adjusted hazard ratios of all-cause and cause-specific mortality by replacing 1 whole egg with equivalent amounts of other protein sources.**

Forest plots show the multivariable HRs of (**A**) total, (**B**) CVD, (**C**) cancer, (**D**) RD, (**E**) AD, and (**F**) diabetes mortality associated with replacing half a whole egg/day with equivalent amount of egg whites/substitutes, poultry, fish, dairy products, or nuts/legumes. HRs were adjusted for age, sex, BMI, race, education, marital status, household income, smoking, alcohol, vigorous physical activity, usual activity at work, hypertension, hypercholesteremia, history of heart disease, stroke, diabetes, and cancer at baseline, total energy intake, and intakes of fruit, vegetables, potatoes, whole grains, refined grains, coffee, and sugar sweetened beverages. Horizontal lines represent 95% CIs. CI, confidence interval; CVD, cardiovascular disease; HR, hazard ratio.

**6. Have the authors considered** **using age as underlying time variable?**

**Answer**: Although using age as underlying time variable could have advantages including easier inferences to interpret and simpler final models, the use of standard survival analysis (follow-up duration as time scale) generally produces correct estimates [29]. Importantly, it has been revealed that the adjustment for age at entry is most important in reducing bias of the estimated coefficients regardless of the choice of time scale [30]. When adjustment for age at entry is made, analyses show very little to suggest that there exists any practically meaningful difference in the estimated regression coefficients depending on the choice of time scale [30].

**7. Did the authors have information on** **the type of fat that was used for frying the eggs? It** **may make a difference.**

**Answer**: We appreciate this thoughtful comment. We agree that the type of fat used for frying the eggs may make a difference as different types of fat had divergent effects on human health. For example, plant-sourced oil used for cooking has been linked with lower risk of mortality in a nationwide cohort study [13]. However, we did not have data on this information. We have discussed this in the Discussion section.

**Page 20, lines 422-426:**

We noted that a few associations such as CVD and diabetes mortality appeared stronger for non-fried eggs compared with fried eggs, which may be due to the type of fat used for frying [24]. For example, plant-sourced cooking oil consumption has been linked with lower risk of mortality in a nationwide cohort study [56].

**8. I think that the figure with the interaction analyses may be included in the main text.**

**Answer**: We thank this comment and have included the results from interaction analyses in the main text as Table 4.

**9. As for the discussion, some further discussion on some causes of death such as respiratory diseases are needed since they may be less familiar to the readers.**

**Answer**: We appreciate this comment. Additionally, we detected adverse associations of egg/cholesterol with death from RD. A cross-sectional study reported that serum cholesterol level was positively associated with airway resistance in patients with chronic obstructive pulmonary disease [31]. In a murine model, dietary cholesterol enhanced pulmonary allergic inflammation [32]. We have added these in the Discussion section.

**Page 19, lines 393-397:**

In addition, we detected adverse associations with risk of death from RD. A cross-sectional study reported that serum cholesterol level was positively associated with airway resistance in patients with chronic obstructive pulmonary disease [44]. In a murine model of asthma, dietary cholesterol enhanced pulmonary allergic inflammation [45].

**10. Also, results for the interactions may be further discussed in the discussion.**

**Answer**: We appreciate this comment. The interaction between sex and egg/cholesterol on mortality could be due to sex dimorphisms in cholesterol catabolism and modulation [33]. Although alcohol drinkers are known to have higher HDL-c and lower LDL-c compared with non-drinkers [34], our results showed a more pronounced positive associations of egg/cholesterol with mortality among alcohol drinkers. Smoking has been evidenced to exacerbate the effect of cholesterol on CVD risk [35]. However, we found a slightly stronger positive associations among non-smokers. Future researches are needed to verify and elucidate these observed interactions. We have included more discussions on the interactions in the Discussion section.

**Page 20, lines 416-422:**

The interaction between sex and egg/cholesterol on mortality could be due to sex dimorphisms in cholesterol catabolism and modulation [53]. Although alcohol drinkers are known to have higher HDL-c and lower LDL-c compared with non-drinkers [54], our results showed more pronounced positive associations of egg/cholesterol with mortality among alcohol drinkers. Smoking has been evidenced to exacerbate the effect of cholesterol on CVD risk [55]. However, we found slightly stronger positive associations among non-smokers. Future researches are needed to verify and elucidate these observed interactions.

**11. Table 1: Please, consider removing the decimals in the dietary variables** **from animal protein to sugar-sweetened beverages. Also, please, include a measure of spread for the quantitative variables.**

**Answer**: Done. We have removed the decimals in the dietary variables from animal protein to sugar-sweetened beverages. Also, we have now showed mean (SD) for quantitative variables for Table 1.

**Table 1. Baseline characteristics of participants from the NIH-AARP Diet and Health Study according to whole egg consumption**

|  | **Quintiles of whole egg consumption** | | | | |
| --- | --- | --- | --- | --- | --- |
| **Characteristics** | **Q1** | **Q2** | **Q3** | **Q4** | **Q5** |
| Range (g/2000 kcal/day) | ≤0.9 | 1.0-5.2 | 5.3-10.3 | 10.4-19.0 | ≥19.1 |
| N | 104,224 | 104,224 | 104,224 | 104,224 | 104,224 |
| Age (y) | 62.6 (5.3)^a^ | 61.9 (5.4) | 62.0 (5.4) | 62.2 (5.3) | 62.3 (5.3) |
| Male (%) | 59.2 | 55.7 | 58.7 | 58.3 | 62.0 |
| Ethnicity (%) |  |  |  |  |  |
| Non-Hispanic white | 90.7 | 91.6 | 92.4 | 92.8 | 91.4 |
| Non-Hispanic black | 4.1 | 4.0 | 3.3 | 2.9 | 3.8 |
| Other/missing | 5.2 | 4.4 | 4.2 | 4.3 | 4.8 |
| BMI (kg/m^2^) | 26.4 (4.8) | 26.7 (4.9) | 27.1 (5.0) | 27.2 (5.0) | 28.0 (5.5) |
| Married (%) | 68.8 | 66.8 | 68.9 | 70.4 | 67.1 |
| College graduate or postgraduate (%) | 43.3 | 40.1 | 38.6 | 38.4 | 34.7 |
| Annual household income (USD)^b^ | 55111 (24694) | 54432 (24125) | 54064 (23413) | 54094 (23358) | 52163 (22450) |
| Current smoker (%) | 6.9 | 11.0 | 12.3 | 12.6 | 15.6 |
| Physical activity, ≥5 times/wk (%) | 25.2 | 19.8 | 17.8 | 16.9 | 16.0 |
| Hypertension (%) | 25.7 | 22.0 | 22.6 | 23.3 | 23.5 |
| High cholesterol level (%) | 18.2 | 26.9 | 27.7 | 28.6 | 29.6 |
| Heart disease (%) | 23.2 | 11.6 | 11.7 | 11.6 | 11.9 |
| Stroke (%) | 2.7 | 1.9 | 1.9 | 1.9 | 2.2 |
| Cancer (%) | 8.9 | 8.6 | 8.9 | 9.2 | 9.4 |
| Diabetes (%) | 9.8 | 6.8 | 7.7 | 8.6 | 12.6 |
| Fair or poor health (%) | 14.0 | 11.3 | 11.5 | 12.2 | 15.5 |
| Currently using vitamin supplements (%) | 58.8 | 55.3 | 55.2 | 55.5 | 53.7 |
| Daily use of aspirin (%) | 20.6 | 13.9 | 13.5 | 13.6 | 12.9 |
| Egg whites/substitutes consumer (%) | 66.0 | 1.9 | 1.4 | 0.9 | 0.4 |
| Daily dietary intake^c^ |  |  |  |  |  |
| Total energy (kcal) | 1723.7 (645.5) | 1842.5 (643.4) | 1880.7 (768.0) | 1794.0 (644.7) | 1763.4 (666.6) |
| Alcohol from alcoholic drinks (g) | 9.6 (23.4) | 11.9 (27.3) | 12.6 (27.9) | 11.3 (24.2) | 10.3 (21.3) |
| Whole egg (g) | 0.1 (0.2) | 3.0 (1.2) | 7.6 (1.5) | 14.0 (2.4) | 34.7 (19.8) |
| Cholesterol (mg) | 148.7 (57.1) | 175.1 (55.6) | 204.4 (53.4) | 236.8 (53.1) | 333.3 (103.5) |
| Total fat (% of energy) | 25.9 (7.5) | 29.2 (7.4) | 30.7 (7.0) | 31.6 (6.8) | 33.9 (7.2) |
| Saturated fat (% of energy) | 7.6 (2.6) | 9.1 (2.8) | 9.7 (2.7) | 10.0 (2.7) | 10.7 (2.9) |
| Monounsaturated fat (% of energy) | 9.7 (3.1) | 10.9 (3.0) | 11.6 (2.9) | 12.0 (2.8) | 12.9 (2.9) |
| Polyunsaturated fat (% of energy) | 6.5 (2.2) | 6.8 (2.3) | 7.0 (2.1) | 7.2 (2.1) | 7.5 (2.2) |
| Trans fat (% of energy) | 1.8 (0.8) | 2.0 (0.8) | 2.1 (0.8) | 2.2 (0.8) | 2.3 (0.9) |
| Animal protein (g) | 46 (19) | 45 (16) | 47 (15) | 48 (15) | 50 (16) |
| Fiber (g) | 26 (9) | 22 (8) | 21 (7) | 21 (7) | 19 (7) |
| Sodium (mg) | 3132 (623) | 2965 (597) | 3005 (557) | 3053 (532) | 3105 (546) |
| Red meat (g) | 50 (40) | 65 (41) | 73 (41) | 76 (41) | 81 (44) |
| Poultry (g) | 55 (51) | 44 (42) | 43 (37) | 43 (37) | 40 (37) |
| Fish (g) | 25 (27) | 21 (22) | 21 (20) | 22 (20) | 22 (22) |
| Dairy products (g) | 400 (337) | 397 (337) | 382 (308) | 373 (292) | 344 (286) |
| Nuts/legumes (g) | 18 (22) | 15 (18) | 15 (16) | 14 (15) | 14 (15) |
| Fruit (g) | 507 (357) | 446 (348) | 410 (308) | 393 (282) | 362 (279) |
| Vegetables (g) | 399 (229) | 336 (196) | 324 (176) | 322 (167) | 313 (173) |
| Potatoes (g) | 59 (48) | 56 (44) | 57 (42) | 59 (41) | 59 (43) |
| Whole grains (g) | 39 (27) | 33 (25) | 31 (23) | 31 (22) | 29 (22) |
| Refined grains (g) | 142 (50) | 133 (48) | 132 (45) | 132 (43) | 127 (44) |
| Coffee (g) | 881 (868) | 887 (865) | 938 (862) | 960 (815) | 1065 (923) |
| Sugar-sweetened beverages (g) | 696 (722) | 689 (702) | 655 (649) | 620 (601) | 638 (684) |
| Healthy Eating Index score | 71.2 (8.9) | 68.0 (9.6) | 67.4 (9.3) | 67.2 (9.2) | 65.2 (9.6) |

BMI, body mass index; Q, quintile.

^a^ Values are means (SDs) or percentages unless stated otherwise.

^b^ Household income in 1999.

^c^ Dietary intakes were energy-adjusted using the nutrient density method of 2000 kcal per day unless stated otherwise.

**12. Also in table 1, for the dietary variables, shouldn't the units be per day, e.g. fiber (g/day)? Finally, I congratulate the authors for their manuscript.**

**Answer**: Yes, the units are g/2000 kcal/day for the dietary variables. We have highlighted this in the footnote of table 1 instead of adding “/2000 kcal/day” for each dietary variables, which is redundant. We thank the reviewer for the congratulations and the efforts for making recommendations for our manuscript.

**Table 1 footnote:** ^c^ Dietary intakes were energy-adjusted using the nutrient density method of 2000 kcal per day unless stated otherwise.

**References**

1.	Valeri L, Vanderweele TJ. Mediation analysis allowing for exposure-mediator interactions and causal interpretation: theoretical assumptions and implementation with SAS and SPSS macros. Psychol Methods. 2013;18(2):137-150. doi: 10.1037/a0031034 PMID: 23379553

2.	Lin DY FT, De Gruttola V. Estimating the proportion of treatment effect explained by a surrogate marker. Stat Med. 1997;16:1515-1527. doi: 10.1002/(sici)1097-0258(19970715)16:13<1515::aid-sim572>3.0.co;2-1 PMID: 9249922

3.	Etemadi A, Sinha R, Ward MH, Graubard BI, Inoue-Choi M, Dawsey SM, et al. Mortality from different causes associated with meat, heme iron, nitrates, and nitrites in the NIH-AARP Diet and Health Study: population based cohort study. BMJ. 2017;357:j1957. doi: 10.1136/bmj.j1957 PMID: 28487287

4.	Zhou G, Yang L, Luo C, Liu H, Li P, Cui Y, et al. Low-to-moderate fluoride exposure, relative mitochondrial DNA levels, and dental fluorosis in Chinese children. Environ Int. 2019;127:70-77. doi: 10.1016/j.envint.2019.03.033 PMID: 30909095

5.	Drouin-Chartier JP, Zheng Y, Li Y, Malik V, Pan A, Bhupathiraju SN, et al. Changes in Consumption of Sugary Beverages and Artificially Sweetened Beverages and Subsequent Risk of Type 2 Diabetes: Results From Three Large Prospective U.S. Cohorts of Women and Men. Diabetes Care. 2019;42(12):2181-2189. doi: 10.2337/dc19-0734 PMID: 31582428

6.	Hu Y, Zong G, Liu G, Wang M, Rosner B, Pan A, et al. Smoking Cessation, Weight Change, Type 2 Diabetes, and Mortality. N Engl J Med. 2018;379(7):623-632. doi: 10.1056/NEJMoa1803626 PMID: 30110591

7.	2015-2020 Dietary Guidelines for Americans. 8th ed: U.S. Department of Health and Human Services and U.S. Department of Agriculture; 2015.

8.	Li MY, Chen JH, Chen C, Kang YN. Association between Egg Consumption and Cholesterol Concentration: A Systematic Review and Meta-analysis of Randomized Controlled Trials. Nutrients. 2020;12(7):1995. doi: 10.3390/nu12071995 PMID: 32635569

9.	Ference BA, Ginsberg HN, Graham I, Ray KK, Packard CJ, Bruckert E, et al. Low-density lipoproteins cause atherosclerotic cardiovascular disease. 1. Evidence from genetic, epidemiologic, and clinical studies. A consensus statement from the European Atherosclerosis Society Consensus Panel. Eur Heart J. 2017;38(32):2459-2472. doi: 10.1093/eurheartj/ehx144 PMID: 28444290

10.	Subar AF, Midthune D, Kulldorff M, Brown CC, Thompson FE, Kipnis V, et al. Evaluation of Alternative Approaches to Assign Nutrient Values to Food Groups in Food Frequency Questionnaires. American Journal of Epidemiology. 2000;152(3):279-286. doi: 10.1093/aje/152.3.279 PMID: 10933275

11.	Dehghan M, Mente A, Rangarajan S, Sheridan P, Mohan V, Iqbal R, et al. Association of dairy intake with cardiovascular disease and mortality in 21 countries from five continents (PURE): a prospective cohort study. Lancet. 2018;392(10161):2288-2297. doi: 10.1016/S0140-6736(18)31812-9 PMID: 30217460

12.	Zhuang P, Zhang Y, He W, Chen XQ, Chen JN, He LL, et al. Dietary Fats in Relation to Total and Cause-Specific Mortality in a Prospective Cohort of 521 120 Individuals With 16 Years of Follow-Up. Circulation Research. 2019;124(5):757-768. doi: 10.1161/CIRCRESAHA.118.314038 PMID: 30636521

13.	Wu F, Mao L, Zhuang P, Chen XQ, Jiao JJ, Zhang Y. Plant-sourced cooking oil consumption is associated with lower total mortality in a longitudinal nationwide cohort study. Clinical Nutrition. 2020. doi: 10.1016/j.clnu.2020.03.031 PMID: 32359931

14.	Li CK, Yang L, Zhang DF, Jiang WJ. Systematic review and meta-analysis suggest that dietary cholesterol intake increases risk of breast cancer. Nutrition Research. 2016;36(7):627-635. doi: 10.1016/j.nutres.2016.04.009 PMID: 27333953

15.	Wang J, Wang WJ, Zhai L, Zhang DF. Association of cholesterol with risk of pancreatic cancer: a meta-analysis. World journal of gastroenterology. 2015;21(12):3711-3719. doi: 10.3748/wjg.v21.i12.3711 PMID: 25834340

16.	Haddy N, Sass C, Droesch S, Zaiou M, Siest G, Ponthieux A, et al. IL-6, TNF-α and atherosclerosis risk indicators in a healthy family population: the STANISLAS cohort. Atherosclerosis. 2003;170(2):277-283. doi: 10.1016/S0021-9150(03)00287-9 PMID: 14612208

17.	Kimura Y, Sumiyoshi M. High-fat, high-sucrose, and high-cholesterol diets accelerate tumor growth and metastasis in tumor-bearing mice. Nutrition And Cancer. 2007;59(2):207-216. doi: 10.1080/01635580701499537 PMID: 18001216

18.	Sakaguchi M, Minoura T, Hiramatsu Y, Takada H, Yamamura M, Hioki K, et al. Effects of Dietary Saturated and Unsaturated Fatty Acids on Fecal Bile Acids and Colon Carcinogenesis Induced by Azoxymethane in Rats. Cancer Research. 1986;46(1):61. PMID: 3940210

19.	Raman G, Berger S, Jacques PF, Vishwanathan R, Johnson EJ. Dietary cholesterol and cardiovascular disease: a systematic review and meta-analysis. The American Journal of Clinical Nutrition. 2015;102(2):276-294. doi: 10.3945/ajcn.114.100305 PMID: 26109578

20.	Lau B, Cole SR, Gange SJ. Competing risk regression models for epidemiologic data. American journal of epidemiology. 2009;170(2):244-256. doi: 10.1093/aje/kwp107 PMID: 19494242

21.	Beslay M, Srour B, Mejean C, Alles B, Fiolet T, Debras C, et al. Ultra-processed food intake in association with BMI change and risk of overweight and obesity: A prospective analysis of the French NutriNet-Sante cohort. PLoS Med. 2020;17(8):e1003256. doi: 10.1371/journal.pmed.1003256 PMID: 32853224

22.	Kunzmann AT, Coleman HG, Huang WY, Berndt SI. The association of lifetime alcohol use with mortality and cancer risk in older adults: A cohort study. PLoS Med. 2018;15(6):e1002585. doi: 10.1371/journal.pmed.1002585 PMID: 29920516

23.	Krebs-Smith SM, Pannucci TE, Subar AF, Kirkpatrick SI, Lerman JL, Tooze JA, et al. Update of the Healthy Eating Index: HEI-2015. J Acad Nutr Diet. 2018;118(9):1591-1602. doi: 10.1016/j.jand.2018.05.021 PMID: 30146071

24.	Drouin-Chartier JP, Schwab AL, Chen S, Li Y, Sacks FM, Rosner B, et al. Egg consumption and risk of type 2 diabetes: findings from 3 large US cohort studies of men and women and a systematic review and meta-analysis of prospective cohort studies. Am J Clin Nutr. 2020;112(3):619-630. doi: 10.1093/ajcn/nqaa115 PMID: 32453379

25.	Guasch-Ferré M, Hruby A, Salas-Salvadó J, Martínez-González MA, Sun Q, Willett WC, et al. Olive oil consumption and risk of type 2 diabetes in US women. The American journal of clinical nutrition. 2015;102(2):479-486. doi: 10.3945/ajcn.115.112029 PMID: 26156740

26.	Freedman ND, Park Y, Abnet CC, Hollenbeck AR, Sinha R. Association of coffee drinking with total and cause-specific mortality. N Engl J Med. 2012;366(20):1891-1904. doi: 10.1056/NEJMoa1112010 PMID: 22591295

27.	Ma H, Li X, Sun D, Zhou T, Ley SH, Gustat J, et al. Association of habitual glucosamine use with risk of cardiovascular disease: prospective study in UK Biobank. BMJ. 2019;365:l1628. doi: 10.1136/bmj.l1628 PMID: 31088786

28.	Zheng Y, Li Y, Satija A, Pan A, Sotos-Prieto M, Rimm E, et al. Association of changes in red meat consumption with total and cause specific mortality among US women and men: two prospective cohort studies. BMJ. 2019;365:l2110. doi: 10.1136/bmj.l2110 PMID: 31189526

29.	Lamarca R, Alonso J, Gomez G, Munoz A. Left-truncated data with age as time scale: an alternative for survival analysis in the elderly population. J Gerontol A Biol Sci Med Sci. 1998;53(5):M337-343. doi: 10.1093/gerona/53a.5.m337 PMID: 9754138

30.	Pencina MJ, Larson MG, D'Agostino RB. Choice of time scale and its effect on significance of predictors in longitudinal studies. Stat Med. 2007;26(6):1343-1359. doi: 10.1002/sim.2699 PMID: 16955538

31.	Rafie S, Moitra S, Brashier BB. Association between the Serum Metabolic Profile and Lung Function in Chronic Obstructive Pulmonary Disease. Turk Thorac J. 2018;19(1):13-18. doi: 10.5152/TurkThoracJ.2017.17043 PMID: 29404181

32.	Yeh YF, Huang SL. Dietary Cholesterol Enhances Pulmonary Eosinophilic Inflammation in a Murine Model of Asthma. International Archives of Allergy and Immunology. 2001;125(4):329-334. doi: 10.1159/000053834 PMID: 11574755

33.	Phelps T, Snyder E, Rodriguez E, Child H, Harvey P. The influence of biological sex and sex hormones on bile acid synthesis and cholesterol homeostasis. Biol Sex Differ. 2019;10(1):52-52. doi: 10.1186/s13293-019-0265-3 PMID: 31775872

34.	Wakabayashi I. Difference in sensitivities of blood HDL cholesterol and LDL cholesterol levels to alcohol in middle-aged Japanese men. Alcohol. 2018;67:45-50. doi: 10.1016/j.alcohol.2017.08.011 PMID: 29425958

35.	Nakamura K, Barzi F, Huxley R, Lam TH, Suh I, Woo J, et al. Does cigarette smoking exacerbate the effect of total cholesterol and high-density lipoprotein cholesterol on the risk of cardiovascular diseases? Heart. 2009;95(11):909. doi: 10.1136/hrt.2008.147066 PMID: 19196734
