# Supplementary material for: Egg and cholesterol consumption and mortality from cardiovascular and different causes in the United States: A population-based cohort study
Source: PLoS Med. 2021 Feb 9;18(2):e1003508. doi: 10.1371/journal.pmed.1003508 (PMC7872242; doi:10.1371/journal.pmed.1003508)
Supplement: Attachment — Submitted filename: Response to reviewers (Plos Med)---20201217.docx [file pmed.1003508.s005.docx]

**EDITORIAL REQUESTS:**

**1. At line 23, please make that "... aged 50-71 years ..." and similar.**

**Answer**: Done. We have now revised this sentence accordingly.

**Lines 23-25:**

Overall 521,120 participants (aged 50-71 years, mean age = 62.2 y, 41.2% women, and 91.8% non-Hispanic white) were recruited from 6 states and 2 cities in the US between 1995 and 1996 and prospectively followed up until 2012.

**2. At line 443, please make that "... the** **study's observational nature.".**

**Answer**: Done. We have now revised this sentence accordingly.

**Lines 442-443:**

Lastly, a causal relationship may not be established considering the study's observational nature.

**PRODUCTION REQUESTS:**

**1. Please provide a detailed Financial Disclosure statement. This is published with the article, therefore should be completed in full sentences and contain the exact wording you wish to be published.**

**Answer**: Done. We have now provided a detailed Financial Disclosure statement.

**Disclaimer**

Y.Z. is supported by the National Key Research and Development Program of China (2017YFC1600500). The funders had no role in study design, data collection and analysis, decision to publish, or preparation of the manuscript.

**1. Please clarify all sources of funding (financial or material support) for your study. List the grants (with grant number) or organizations (with url) that supported your study, including funding received from your institution.**

**Answer**: Done. We have clarified all sources of funding (financial or material support) for our study.

**2. State the initials, alongside each funding source, of each author to receive each grant.**

**Answer:** Done. We have stated the initials of each author to receive each grant.

**3. State what role the funders took in the study. If the funders had no role in your study, please state:** **“The funders had no role in study design, data collection and analysis, decision to publish, or preparation of the manuscript.”**

**Answer:** Done. We have stated “The funders had no role in study design, data collection and analysis, decision to publish, or preparation of the manuscript.”.

**4. If any authors received a salary from any of your funders, please state which authors and which funders.**

**If you did not receive any funding for this study, please state: “The authors received no specific funding for this work.”**

**Answer:** The authors received no salary from any of the funders.

**2. Please** **move your disclaimer to the end of the Acknowledgments section of your manuscript, with a line break between the main text of the acknowledgments and the disclaimer statement.**

**Answer:** Done. We have now moved the disclaimer to the end of the Acknowledgments section.

**3. Please name and describe your "S1 Protocol" file as either "****S1 Protocol" or "S1 Text", to avoid confusion. Please note that the file name and the way the file is referred to in the manuscript must be consistent with each other.**

**Answer:** Done. We have now renamed the file name “S1 Protocol” to “S1 Text” to make it consistent with the way the file is referred to in the manuscript.
